# A novel bile salt hydrolase-producing *Ligilactobacillus salivarius* prevents diet-induced obesity via regulation of bile acid metabolism and glucagon-like peptide 1 restoration

**DOI:** 10.1080/19490976.2026.2668127

**Published:** 2026-05-06

**Authors:** Jiayao Lv, Lanqi Zhou, Xiaoshuang Dai, Rikard Landberg, Huicui Meng, Honglei Tian, Shiyi Zhang, Tianqi Liu, Xiaochen Yin, Jiayi Zhang, Xizi Song, Christophe Bonny, Stephanie Blum, Youshen Cao, Jingyao Guo, Wen Peng, Yan Tan, Lin Shi

**Affiliations:** a School of Food Engineering and Nutritional Science, Shaanxi Normal University, Xi'an, Shaanxi, China; b College of Food Science and Technology, Huazhong Agricultural University, Wuhan, China; c Xbiome, Scientific Research Building, Room 907, Tsinghua High-Tech Park, Shenzhen, China; d Department of Life Sciences, Food and Nutrition Science, Chalmers University of Technology, Gothenburg, Sweden; e School of Public Health (Shenzhen), Sun Yat-Sen University, Shenzhen, Guangdong, China; f School of Aerospace Medicine, Fourth Military Medical University, Xi'an, China; g Global Health Institute, School of Public Health, Xi'an Jiaotong University, Xi'an, Shaanxi, China; h Department of Public Health, Qinghai University Medical College, Xining, China; i Department of Public Health Medical College, Nutrition and Health Promotion Center, Qinghai University, Xining, China; j Qinghai Provincial Key Laboratory of Prevention and Control of Glucolipid Metabolic Diseases with Traditional Chinese Medicine, Medical College, Qinghai University, Xining, China

**Keywords:** Obesity, BSH, *Ligilactobacillus salivarius*, GLP-1, UDCA, gut–liver axis

## Abstract

Obesity poses a major global health challenge, necessitating safe and effective therapeutic strategies. Using high-throughput genomic screening for bile salt hydrolase (BSH)-producing strains, we identified *Ligilactobacillus salivarius* (*L. salivarius*) XA1416, a strain isolated from the feces of healthy individuals, which exhibits high BSH activity, strong acid resistance, and efficient colonization in the gastrointestinal tract. Oral administration of *L. salivarius* XA1416 counteracted high-fat diet-induced weight gain in mice, improved glucose homeostasis, and enhanced GLP-1 secretion. The strain modulated the gut microbiota, enriching taxa such as *Bacteroides*, *Alistipes*, and *Faecalibaculum*, and altered bile acid profiles, notably increasing ursodeoxycholic acid (UDCA). Mendelian randomization analysis leveraging large-scale human GWAS data and two cross-sectional cohorts’ data, complemented by *in vitro* fecal microbiota fermentation experiments, collectively supports a key role of UDCA in weight control. The oral administration of UDCA recapitulated the anti-obesity effects and metabolic benefits of XA1416, functioning as an intestinal Farnesoid X receptor antagonist and Takeda G-protein-coupled receptor 5 agonist to stimulate GLP-1 secretion. This mechanism likely involves modulation of the hepatic FXR/SHP/SREBP-1c pathway and concurrent activation of the GLP-1 receptor, contributing to improved metabolic homeostasis. Additionally, we gavaged the mice fed with normal chow or a high-fat diet with GR-7, a specific inhibitor of microbial BSHs, leading to a reduction in UDCA level and GLP-1 production. Using HepG2 cell models and molecular dynamics simulations, we further demonstrated that UDCA directly activates the GLP-1 receptor. Taken together, our findings position *L. salivarius* XA1416 as a promising anti-obesity probiotic, with UDCA serving as a key microbial metabolite that mediates its beneficial metabolic effects.

## Graphical abstract

**Figure:**
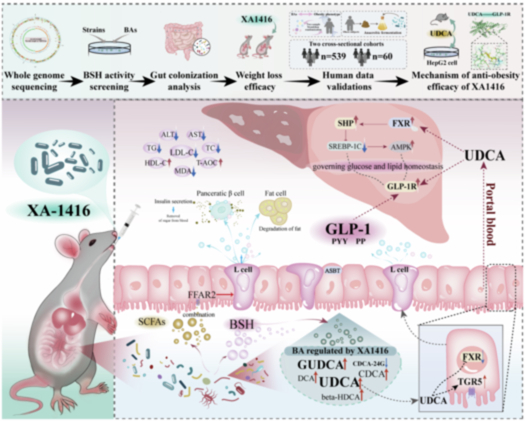

## 1. Introduction

Obesity emerges as a pressing global health crisis, driving the escalating burden of non-communicable diseases and mortality worldwide.^1^^,^^2^ The World Obesity Atlas 2025 reports that 24% of the global population will suffer from obesity by 2035.^3^ While anti-obesity drugs (e.g., orlistat and semaglutide) exist, their costs and adverse side effects are notable,^4^ necessitating the development of more effective therapeutic approaches with fewer adverse effects. Growing evidence highlights the gut microbiota as a key regulator of host metabolism and energy homeostasis,^5^^,^^6^ positioning it as a promising target for obesity intervention.^7^^,^^8^

Probiotics promote host health by restructuring the gut microbiome and reinstating metabolic homeostasis.^9^^,^^10^ Central to this therapeutic capability is the activity of bile salt hydrolase (BSH), a highly conserved microbial enzyme that acts as the fundamental gateway in bile acid (BA) metabolism.^11^ By catalyzing the deconjugation of host-secreted primary BAs, BSH dictates multiple pathophysiological mechanisms linked to obesity mitigation.^12-14^ For instance, deconjugation increases BA hydrophobicity, thereby reducing mixed micelle formation and inducing mild lipid malabsorption that limits energy harvest and adiposity.^15^ Concurrently, the increased fecal excretion of unconjugated BAs disrupts the enterohepatic circulation, which forces the liver to consume systemic cholesterol for *de novo* BA synthesis (e.g., via CYP7A1), thereby ameliorating hepatic steatosis.^16^ Furthermore, BSH may also possess acyltransferase activities, producing a diverse repertoire of microbially conjugated bile acids regulating host metabolic networks.^17^^,^^18^ Abundant in several prominent probiotic genera, including *Lactobacillus*, *Bacteroides*, and *Bifidobacterium*, BSHs thereby represent multifaceted therapeutic targets for the management of metabolic syndrome.

Notably, glucagon-like peptide-1 (GLP-1), an incretin hormone secreted by intestinal L-cells, exerts potent anorexigenic and anti-obesity properties.^19^^,^^20^ Emerging evidence suggests that gut microbiota-modified secondary bile acids may regulate GLP-1 secretion through activation of bile acid-regulated transcription factors, i.e., transmembrane receptor Takeda G-protein-coupled receptor 5 (TGR5) and the nuclear Farnesoid X receptor (FXR) signaling,^21^^,^^22^ thereby inducing GLP-1 secretion. Deciphering the precise mechanisms underlying this probiotic-bile acid-GLP-1 axis could unlock novel therapeutic strategies for obesity intervention.

In the present study, we identified *Ligilactobacillus salivarius* (*L. salivarius*) XA1416, a potent BSH-producing strain, through high-throughput genomic screening. Isolated from the feces of healthy individuals, this strain exhibited strong gastric acid tolerance and efficient intestinal colonization, as demonstrated by both *in vitro* and *in vivo* assessments. *L. salivarius* XA1416 conferred robust protection against diet-induced obesity in mice by modulating bile acid metabolism and enhancing GLP-1 secretion. We further demonstrated that ursodeoxycholic acid (UDCA), a microbial metabolite, was elevated upon *L. salivarius* XA1416 administration, recapitulating the anti-obesity effects and metabolic benefits via a gut–liver axis. By integrating results from Mendelian randomization analysis leveraging large-scale human GWAS data, cross-sectional cohort data, *in vitro* fecal microbiota fermentation experiments, as well as *in vitro* HepG2 cell models and molecular dynamics simulations, our findings collectively establish *L. salivarius* XA1416 as a promising probiotic for obesity prevention, with UDCA serving as a key mediator of its beneficial metabolic effects.

## 2. Methods

### 2.1. Identification of BSH-producing strains through genomic screening technology

The whole genomes of 6 experimental strains, i.e., *Bifidobacterium animalis* (XA-768), *Bifidobacterium adolescentis* (XA-1069), *Ligilactobacillus salivarius* (XA-1416), *Lactiplantibacillus plantarum* (XA-314), *Bifidobacterium longum* (XA-1102), and *Bifidobacterium longum subsp. Suillum* (XA-7822) was sequenced using Nanopore PromethION and Illumina NovaSeq by Novogene, and the generated reads were assembled with SPAdes 3.15.3^23^ (Table S1).

Prodigal (V1.14.5)^24^ was used to predict protein-coding genes in bacterial genomes. Bile acid metabolism genes were identified via two approaches: BLASTp searches against the National Center for Biotechnology Information (NCBI) protein database with top hits with >90% identity maintained, and PLMSearch^25^ for detecting remote homologs of *bsh* genes. PLMAlign^25^ was subsequently employed for structural alignment and scoring (similarity > 0.9, score > 100). Genomes meeting either criterion were retained. BSH enzymatic activity was predicted using Boltz2, and IC₅₀ values were estimated to quantify binding affinity.

BSH activity in the supernatant of culture was measured by the hydrolysis of 1.2 mM conjugated bile acid solution (i.e., glycocholic acid, taurocholic acid, glycochenodeoxycholic acid, taurochenodeoxycholic acid, glycodeoxycholic acid, and taurodeoxycholic acid, all purchased from Sigma) in M9 culture medium (40 g/L glucose, 6 g/L Na₂HPO₄, 3 g/L KH₂PO₄, 0.5 g/L NaCl, 1 g/L NH₄Cl, 0.01 g/L CaCl₂, 0.24 g/L MgSO₄, purchased from Sigma). Bacteria were harvested at the early stationary phase and transferred into conjugated bile acid solutions for subsequent assays. After cultivation for 3 h, the supernatants were collected, and bile acids were analyzed using liquid chromatography‒tandem mass spectrometry (LC‒MS/MS) (Metware, Wuhan, China). Separation was performed on an ExionLC™ AD UPLC system (Sciex, United States) equipped with a Waters ACQUITY UPLC HSS T3 C18 column (100 × 2.1 mm, 1.8 µm) maintained at 40 °C. A 3 µL sample was injected and eluted at a flow rate of 0.35  mL/min using a gradient of (A) ultrapure water with 5 mmol/L ammonium acetate and (B) methanol, both containing 0.01% acetic acid. The gradient started at 5% B, increased to 40% B at 0.5 min, 49% at 1.2 min, 50% at 4.5 min, 75% at 4.5 min, 95% at 10 min, and then returned to 5% B at 12 min for re-equilibration. Detection was carried out on a QTRAP® 6500 +  mass spectrometer (Sciex, Germany) with electrospray ionization in negative mode, using scheduled multiple reaction monitoring. The key parameters included: ion spray voltage of 4500  V, a source temperature of 550 °C, and curtain gas at 35  psi.

BSH activity was quantified as the rate of decrease in conjugated bile acid concentration over the 3 h incubation.

### 2.2. Isolation, gastric acid tolerance assessments, and intestinal colonization

*L. salivarius* XA1416 was initially isolated from healthy human feces and has been recorded by the Center for General Microbiology, China Microbial Strain Preservation and Management Committee, China (CGMCC No. 40307). Healthy stool donors were recruited from a pool of healthy volunteers who successfully completed a stringent donor qualification process, including truthful completion of the screening questionnaire, an in-person interview, all required laboratory tests, and negative results on all gastrointestinal pathogen detection tests.

Specifically, under anaerobic conditions, 29 frozen fecal samples from healthy donors in Shenzhen were equally pooled to prepare a mixed sample. A 0.5 mL aliquot of the mixed sample was transferred into an anaerobic blood culture flask and incubated statically at 37 °C for 3 d. Subsequently, 0.1 mL of the cultured mixture was serially diluted (10-fold gradients) in PBS buffer (Solarbio, Cat# P1020) containing 1 g/L L-cysteine hydrochloride, yielding dilutions from 10^−1^ to 10^−7^. Aliquots (100  μL) of 10^−5^, 10^−6^, and 10^−7^ dilutions were spread onto deoxygenated YCFA solid culture medium (triplicate per dilution) and incubated anaerobically at 37 °C for 3 d. Single colonies were inoculated into deoxygenated BHI liquid medium and cultured under identical conditions. The isolates were numbered and subjected to Gram staining, species identification, physiological characterization, and genomic analysis. Strains exhibiting typical *L. salivarius* features were selected, yielding strain *L. salivarius* XA1416. The number of living cells was determined by counting the number of colony-forming units (CFUs).

To assess gastric acid tolerance, the bacteria (1 × 10^8^ CFU/mL) were cultured at 37 °C in Gifu Anaerobic Medium (GAM) culture medium. The cell density of OD600 was measured at 24 h according to the method described elsewhere.^26^ Then, the bacteria *L. salivarius* XA1416 were exposed to simulated gastric (pH 1.2) and small intestinal (pH 6.8) fluids, with viability assessed at 0, 0.5, 1, 2, and 3 h post-exposure.

**Figure 1.**
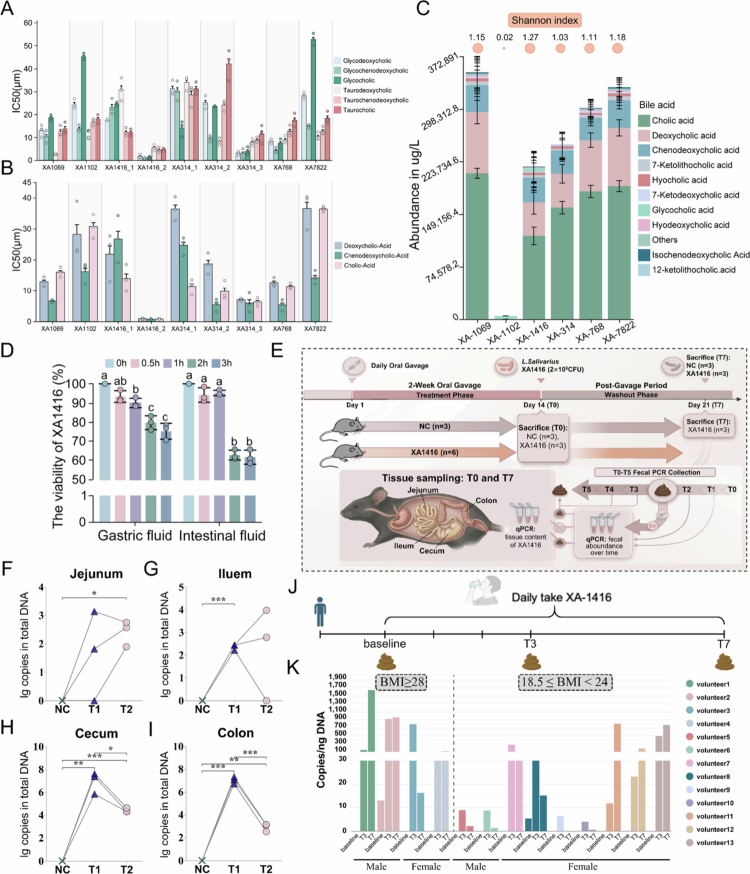
Identification and colonization of *L. salivarius* XA1416. Predicted binding affinity of the *bsh* gene from selected genomes for (A) substrates and (B) products. (C) The distribution and diversity of bile acids were detected in the supernatant from the culture media. The circles on the top represent the Shannon diversity index calculated for each supernatant. (D) Viability of *L. salivarius* XA1416 in simulated gastrointestinal fluids *in vitro*. (E) The *in vivo* experimental design. The cumulative colonization of *L. salivarius* XA1416 in the (F) jejunum, (G) ileum, (H) cecum, and (I) colon of mice. (J) The feces sampling for detecting the presence of *L. salivarius* XA1416. (K) The copies/ng DNA of *L. salivarius* XA1416 in human feces. NC: normal diet. XA1416: administered with 2 × 10^8^CFU/mouse *L. salivarius* XA1416. Differences between multiple groups were assessed by ANOVA, followed by Tukey’s post-hoc test. Significance was indicated by different letters (*p* < 0.05). Student’s t-test was used to evaluate differences between two groups. *p < 0.05; **p < 0.01; ***p < 0.001.

To investigate the intestinal colonization of *L. salivarius* XA1416 *in vivo*, 9 mice were randomly divided into a normal control (NC, *n* = 3) and an *L. salivarius* XA1416-treated group (oral gavage, 2 × 10^8^CFU per day for 2 weeks, *n* = 6, Figure 1E). Fecal samples were collected at 0, 1, 2, 3, 4, and 5 d after the cessation of administration. The jejunum, ileum, cecum, and colon tissues were collected exclusively after the initial 2-week administration and at day 7 post-cessation, followed by immediate storage for downstream analysis.

To further detectits presence in human feces,, a pilot study was conducted in 13 subjects who had not been exposed to the *L. salivarius* XA1416 before. Females (*n* = 9) and males (*n* = 4) comprising 4 overweight (BMI ≥ 28) and 9 normal-weight (18.5 ≤ BMI < 24) individuals were included. All participants provided written informed consent, and the study protocol was approved by the Ethics Committee of Shaanxi Normal University (No. 202416037). Packaged probiotic was provided by Jinhua Yinhe Biotech (Jinhua,China). In brief, after collection of a baseline fecal sample (day 0), participants received a daily oral dose of 2.0 × 10¹⁰ CFU of *L. salivarius* XA1416 for 7 consecutive days. Fecal samples were collected at 3 and 7 d after administration. All samples were immediately stored for analysis.

To quantify intestinal colonization by *L. salivarius* XA1416, we designed strain-specific primers based on whole-genome sequencing data. Briefly, we identified unique genomic sequences by aligning *L. salivarius* XA1416’s genome against the NCBI nucleotide database, followed by primer design using NCBI Primer-BLAST (Table S2). Fecal DNA was extracted using a commercial kit (Stool Genomic DNA Kit, CWBIO), and *L. salivarius* XA1416 abundance was assessed via qPCR analysis.

### 2.3. Animal experiments

To evaluate the anti-obesity potential of *L. salivarius* XA1416, 32 healthy 5-week male C57BL/6J mice were randomly allocated into four groups (*n* = 8 per group) for 11 weeks: (1) normal chow diet (NC), (2) high-fat diet (HFD), (3) HFD supplemented with orlistat (10  mg/kg, administered via oral gavage daily), and (4) HFD supplemented with *L. salivarius* XA1416 (2 × 10^8^ CFU per mouse via oral gavage daily). Body weight and food intake were monitored weekly. Mice were purchased from Charles River, Beijing, China. Animal experiments were conformed to the Guide for the Care and Use of Laboratory Animals, Eighth Edition, ISBN-10: 0-309-15396-4. The protocol has been approved by the Animal Ethics Committee of GnotoBio biotechnology (No. JTAW20240401-2).

To evaluate the anti-obesity effects of UDCA, healthy 5-week-old male C57BL/6J mice were randomly allocated into six groups for 10 weeks: normal control (NC, *n* = 7), HFD (*n* = 7), HFD supplemented with orlistat (10 mg/kg, *n* = 7), HFD supplemented with *L. salivarius* XA1416 (2 × 10^8^ CFU per mouce, *n* = 5), and two UDCA treatment groups (10 mg/kg and 50 mg/kg, *n* = 7 per group). Mice were purchased from the Experimental Animal Center of Shaanxi Normal University (Xi’an, China). Animal experiments have been approved by the Ethics Committee of Shaanxi Normal University (No. 202516078). Body weight and food intake were monitored every 3 d, and water intake was measured weekly. Magnetic resonance imaging was used to assess body fat and lean mass on a 7 T Clinscan system (Bruker, Ettlingen, Germany).

To confirm the roles of microbial BSHs on UDCA production, we gavaged mice with the specific BSH inhibitor Gut Restricted-7 (GR-7) (HY135747, MedChemExpress). Twenty 6-week-old male C57BL/6J mice were randomly divided into four groups (*n* = 5) and treated for 4 weeks as follows: (1) NC supplemented with *L. salivarius* XA1416 (2 × 10^8^ CFU per mouse); (2) NC supplemented with *L. salivarius* XA1416 and GR-7 (10 mg/kg); (3) HFD supplemented with *L. salivarius* XA1416; (4) HFD supplemented with *L. salivarius* XA1416 and GR-7 (10 mg/kg). GR-7 was dissolved in corn oil containing 5% DMSO and administered by oral gavage at a volume of 200  μl per mouse every other day.

During experiments, all mice were maintained under specific pathogen-free conditions in a controlled facility with free access to chow and water (22 ± 2 °C, 60 ± 5% humidity, 12 h light/dark cycle). The ingredients of NC were 40% corn flour, 26% wheat flour, 10% bran, 10% fish meal, 10% bean cake, 2% mineral, 1% coarse grains, and 1% vitamin (purchased from Qianmin Feed Factory, Jiangsu, China). The high-fat diet contained 60% fat, 20% carbohydrates, and 20% protein.

At the end of the intervention period, fecal samples were collected 12 h after the final administration. Following euthanasia, blood, liver, and colon tissues were harvested. Tissues were rinsed in ice-cold phosphate-buffered saline and either snap-frozen in liquid nitrogen and stored at −80 °C or fixed in 4% paraformaldehyde for histopathological analysis. Serum was isolated by centrifugation at 2500 ×  g for 15 min at 4 °C and stored at −80 °C until further analyses.

### 2.4. Biochemical parameters analyses

Fasting blood glucose and an oral glucose tolerance test (OGTT) were measured before the animals were sacrificed. After 12 h of fasting, all animals received an oral gavage of glucose (1.5 g/kg body weight).^27^ Blood glucose levels were measured by tail clipping at 0, 15, 30, 60, 90, and 120 min after glucose administration, and the area under the curve (AUC) was calculated.

Content of total cholesterol (TC), triglyceride (TG), alanine aminotransferase (ALT), aspartate aminotransferase (AST), superoxide dismutase (SOD), high density lipoprotein cholesterol (HDL-C), low density lipoprotein cholesterol (LDL-C), total antioxidant capacity (T-AOC) and malondialdehyde (MDA) in serum were measured using commercial kits (Jiancheng Biotechnology Ltd., Nanjing, China) according to the protocols. Protein expressions of GLP-1, leptin, pancreatic polypeptide (PP), and interleukin 10 (IL-10) in the colon tissues were determined using ELISA (Shanghai Yuanju Biotechnology Center, Shanghai, China).

### 2.5. Histological and immunohistological assessments

The colon and liver tissues (1 × 1 × 0.5  cm) were fixed in 4% paraformaldehyde and embedded in paraffin. Samples underwent deparaffinization with xylene and were rehydrated using a graded series of ethanol from 100% to 70%, followed by rinsing in PBS (pH 7.4). Colon samples were stained with H&E, PAS, and Alcian blue for general morphological analysis using an optical microscope (Olympus, × 200). Lipid accumulation in the liver samples was evaluated using Oil Red O and H&E staining on cryosections. Free fatty acid receptor 2 (FFAR2, also known as GPR43) in the colon and FXR in the liver were analyzed via immunohistochemistry (IHC) for their localization and expression.^28^ In brief, tissue sections were dewaxed, rehydrated, and incubated with primary antibodies (occluding, ZO-1, adenosine 5’-monophosphate (AMP)-activated protein kinase (AMPK), and *p*-AMPK, 1:500) at 4 °C overnight. Subsequently, tissue sections were stained using CY3-conjugated secondary antibodies, and DAPI was used for nuclear visualization using a 3DHISTECH Pannoramic MIDI scanner and analyzed using CaseViewer software.

### 2.6. Assessments of mRNA and protein expressions

RNA extraction and qPCR analysis were performed based on methods previously described.^29^ Briefly, total tissue RNA was extracted with the TRIzol reagent (Mei5 Biotechnology Co., Ltd., Beijing, China) from samples. After purification, the RNA was used to synthesize cDNA with a High-Capacity cDNA Reverse Transcription Kit (Mei5 Biotechnology Co., Ltd., Beijing, China), and qPCR was carried out using their SYBR GREEN Master Mix and was determined on a real-time PCR detection system (Bio-Rad, USA). The GADPH gene was used as the internal control and the relative mRNA level of the gene (folds of control) = 2^−ΔΔCT^. Primer sequences are supplemented in Table S2. Protein expressions were measured according to a previous study.^2^ Total protein was extracted from tissues and quantified with a BCA assay kit (Jiancheng Biotech, Nanjing, China). Proteins were separated by 10% SDS‑PAGE and transferred onto PVDF membranes via wet transfer. After blocking with 5% skim milk in TBST for 2 h at room temperature, the membranes were incubated overnight at 4 °C with primary antibodies, followed by HRP‑conjugated secondary antibodies for 1.5 h at room temperature. Signals were developed using Super‑Signal ECL substrate, captured with a ChemiDoc Imaging System (Bio‑Rad Laboratories, Germany), and analyzed by densitometry with ImageJ software.

### 2.7. 16S rRNA sequencing and short-chain fatty acids assessments

Fecal DNA was extracted from mouse samples using the QIAamp Fast DNA Stool Mini Kit (QIAGEN, Cat. No. 47016) following the manufacturer’s instructions. The hypervariable V4 region of bacterial 16S rRNA was amplified in 50  μL reactions containing 25  μL of 2 × Premix Taq, 1  μL of each primer (10  μM), and 3  μL DNA template (20 ng/μL) under the following thermal cycling conditions: initial denaturation at 94 °C for 5  min; 30 cycles of 94 °C for 30 s, 52 °C for 30 s, and 72 °C for 30 s; final extension at 72 °C for 10  min. PCR products were verified by 1% agarose gel electrophoresis, purified, and subsequently processed for library preparation using the NEBNext® Ultra™ II DNA Library Prep Kit for Illumina®. Library quality was assessed using a Qubit 2.0 Fluorometer, followed by paired-end sequencing (2 × 250 bp) on the Illumina NovaSeq 6000 platform. Raw sequences were processed using cutadapt (v3.4) to remove adapters, primers, and poly-A tails. Quality-filtered reads were analyzed with DADA2 (v1.22) for amplicon sequence variant (ASV) calling and taxonomic classification against the SILVA 138 database. ASVs with a mean abundance of < 0.1% were filtered prior to downstream analysis. Functional prediction was performed using PICRUSt2 (v2.4.1). We also analyzed colonic concentrations of short-chain fatty acids (SCFAs) using gas chromatography−mass spectrometry (Agilent Technologies, Santa Clara, CA, US). Detailed information on the analytical procedure has been published elsewhere.^30^

### 2.8. Quantification of bile acid profiles

Bile acid contents were detected by MetWare (http://www.metware.cn/) using the AB Sciex QTRAP 6500 LC–MS/MS platform.^31-33^ In brief, 20  μL samples were mixed with 60  μL of pre-cooled 50% methanol, vortexed for 5 min, and incubated at -20 °C for 4 h. After centrifugation at 20,000 × g (4 °C, 15 min), 20  μL supernatant was derivatized with 20  μL of 200  mM 3-nitrophenylhydrazine and 20  μL of 120  mM EDC containing 6% pyridine at 25 °C for 30 min. Chromatographic separation was performed on a Waters BEH C18 column (2.1 × 100 mm, 1.7 μm) using a gradient of mobile phase A (water with 0.1% formic acid) and B (methanol with 0.1% formic acid) at 0.35  mL/min (2% B at 0–2 min, 20%–80% B at 2.5–15 min). Raw data were processed with MultiQuant software (SCIEX) for peak integration and quantification. Mass spectrometric detection was conducted on an AB Sciex 6500+  system in the multiple reaction monitoring (MRM) mode. MRM transitions were optimized for individual bile acids, which exhibited high reproducibility, enabling robust quantification of 45 bile acids, including free bile acids and conjugated bile acids (Table S3).

To ensure analytical quality, nine-point calibration curves were established using serial dilutions of standard mixtures. The standard solutions were prepared at concentrations ranging from 0.1 to 1000 ng/mL (specifically: 0.1, 0.2, 0.4, 1, 2, 4, 10, 20, 40, 100, 200, 400, and 1000 ng/mL). All standards were purchased from CNW Technologies and IsoReag (both in Shanghai, China). Individual stock solutions were prepared in methanol at a concentration of 1 mg/mL and stored at −20 °C. Prior to analysis, these stock solutions were diluted with methanol to obtain the required working concentrations. The calibration curves were constructed by plotting the Concentration Ratio (the ratio of the analyte concentration to the internal standard concentration) on the x-axis against the Area Ratio (the ratio of the analyte peak area to the internal standard peak area) on the y-axis. The corresponding linear equations, correlation coefficients, and related parameters for all analyzed substances are summarized in Table S3.

### 2.9. The bidirectional two-sample Mendelian randomization (MR) analyses

We obtained GWAS summary statistics from the Integrative Epidemiology Unit (lEU) and the GWAS catalog online database. Due to the source and nature of the data, no additional ethical review or informed consent was required for this study. Analyses followed STROBE-MR guidelines. We utilized genetic associations for four bile acids as primary exposures: UDCA (met-a-346, 5,477 Europeans, 2,543,395 Single Nucleotide Polymorphisms (SNPs), CDCA (GCST90616309, 2,392 Europeans, 10,448,203 SNPs), GUDCA (GCST90200221, 6,622 Europeans, 15,359,645 SNPs), and TUDCA (GCST90616254, 2,392 Europeans, 10,448,203 SNPs). Outcome data included BMI (ukb-a-248, 336,107 Europeans, 10,894,596 SNPs), fasting glucose (ieu-b-4761, 46,186 Europeans, 2,529,804 SNPs), appetite (PROT-a-1212, 3,301 Europeans, 10,534,735 SNPs), insulin-like grown factor1 (PROT-a-1443, 3,301 Europeans, 10,534,735 SNPs), fasting insulin (EBI-a-GCST005185, including 51,750 Europeans and 2,598,774 SNPs) and weight (ukb-e-21002_CSA, 8,771 South Asian, 9,811,796 SNPs). In the reverse MR analysis, exposures and outcomes were swapped to examine potential bidirectional causal relationships.

This study was conducted in accordance with the quality control steps. First, we selected exposure-related GWAS data and screened SNP loci with genome-wide significance (*p* < 5 × 10^−5^) for pooled aggregation. Second, to avoid linkage disequilibrium (LD) from affecting the results, we performed a clustering process by setting the parameter (r^2^) threshold (r^2^ < 0.001 and region width = 10,000 kb) to assess LD among SNPs to ensure independence. Instrument strength was evaluated using the F-statistic (F > 10) to minimize weak instrument bias in causal effect estimation.

MR analysis was conducted using the two-sample MR (R package, v0.4.20). The inverse-variance weighted (IVW) method served as the primary analysis to estimate causal effects, with odds ratios (ORs) and 95% CIs reported. Sensitivity analyses included MR-Egger, weighted median, and mode-based methods to verify robustness. Heterogeneity was assessed using Cochran’s Q test, applying random-effects IVW when present. Pleiotropy was evaluated via the MR-Egger intercept. Leave-one-out analysis identified influential SNPs, which were excluded in subsequent validation.

### 2.10. *In vitro* fecal microbiota fermentation experiments

Fresh fecal samples were collected from eight donors who were newly diagnosed, treatment-naïve type 2 diabetes patients with BMI > 28, who maintained habitual Chinese dietary patterns and had not used antibiotics within the preceding 3 months. All donors were recruited from the Second Affiliated Hospital of Xi’an Jiaotong University. Informed consent was obtained from all eligible participants prior to sample collection, and all of the procedures were approved by the Ethics Committee of Xi’an Jiaotong University Health Science Center (2017-446). Using their microbiota allowed us to accurately evaluate the restorative capacity of *L. salivarius* XA1416 on an impaired gut microbiome. Anaerobic fermentation was performed following established protocols.^34^^,^^35^ Briefly, fecal samples were delivered within 1 h of defecation and immediately transferred and processed. In the workbench, fresh fecal samples were suspended in PBS (1:5, w/v), homogenized, and centrifuged. The resulting supernatants were handled in an anaerobic chamber (Punmicro, IPAN A610), aliquoted into 5-mL cryotubes, combined with sterile glycerol (1:1, v/v), and preserved at −80 °C.

To evaluate the regulatory effects of *L. salivarius* XA1416 on the microbiota of obese patients, we compared obese microbiota (OB group) and OB + *L. salivarius* XA1416 (1:9). All strains were activated in Gifu anaerobic medium (GAM), with the strictly anaerobic *L. salivarius* XA1416 subcultured thrice in an anaerobic workstation (37 °C, 10% H₂/10% CO₂/80% N₂) to ensure viability. For fecal microbiota resuscitation, 1 mL of −80 °C stock (preserved in 50% glycerol) was mixed with 9 mL pre-reduced GAM and incubated anaerobically at 37 °C. At 24 h, quadruplicate samples per group were collected, immediately centrifuged (4 °C, 6000 × g, 5 min), and processed. Filtered supernatants were aliquoted into RNase-free tubes, sealed, and stored at −80 °C for short-chain fatty acids and bile acid analysis.

### 2.11. The cross-sectional assessments on associations between UDCA and obesity phenotypes

Associations between fecal levels of UDCA with body weight and its related indices were assessed cross-sectionally in two populations: one included college students aged ranged from 18 to 28 (female = 30, male = 30), whose fecal samples were collected during January and June 2024. The study received approval from the Ethics Committee of Shaanxi Normal University (202416036). Another one consisted of participants belonging to the subset of a community-based open cohort study involving two Tibetan communities in the Golmud suburbs. Individuals included in this analysis had complete information on sociodemographic variables, dietary intake, lifestyle behaviors, anthropometric indices, and biochemical parameters, together with high-quality serum metabolomic and metagenomic profiles (*n* = 539).^36-38^ Details of geographical features, background of the communities, anthropometric and biochemical measurements of participants have been previously described. The study adhered to the principles of the Declaration of Helsinki and received ethical approval from the Ethics Committee of the Medical College, Qinghai University (2017-34, 2021-15). Among the population, obesity was defined as BMI ≥ 28 kg/m^2^ based on the Chinese criteria. Overweight was defined as 24  kg/m^2^ ≤ BMI ≤ 28 kg/m^2^. Central obesity was defined as a waist circumference ≥ 90 cm for men and a waist circumference ≥ 80 cm for women based on the International Diabetes Federation criteria. Body fat composition was measured using Inbody 270.

The fecal metabolomes of both studies were assessed by Majorbio Bio-Pharm Technology Co. Ltd. (Shanghai, China).^39^ The LC–MS/MS analysis of samples after extraction was conducted on a Thermo UHPLC-Q Exactive HF-Xsystem equipped with an ACQUITY HSS T3 column (100 mm × 2.1 mm i.d., 1.8 μm; Waters, USA). The mass spectrometric data were collected using a Thermo UHPLC-Q Exactive HF-X Mass Spectrometer equipped with an electrospray ionization source. Data acquisition was performed with the Data Dependent Acquisition mode. The pretreatment of LC/MS raw data was performed by Progenesis QI (Waters Corporation, Milford, USA), and a three-dimensional data matrix in CSV format was exported. Metabolites were identified by matching m/z and MS spectra to an in-house library of Majorbio Database, HMDB (http://www.hmdb.ca/), and Metlin (https://metlin.scripps.edu/).

### 2.12. Assessments on the direct effects of UDCA on HepG2 cells

HepG2 cells were maintained in DMEM supplemented with 10% FBS at 37 °C under 5% CO₂. For experiments, cells were seeded at 2 × 10⁵ cells per well and treated with or without 30 mmol L^−1^ glucose and various concentrations of UDCA (50–200 μmol L^−1^) for 12 h. Cell viability was assessed using the CCK-8 assay following incubation with 10  μL reagent per well for 2 h, absorbance was measured at 450 nm.

For IHC, cells were fixed in 4% paraformaldehyde, blocked with BSA, and incubated sequentially with primary antibodies (against AMPK and *p*-AMPK) and corresponding secondary antibodies. Nuclei were counterstained with DAPI. Slides were scanned using a 3DHISTECH Pannoramic MIDI scanner and analyzed with CaseViewer software. GLP-1 expression and localization were evaluated via IHC. Lipid accumulation was examined by Oil Red O staining after fixing cells with 10% formaldehyde. Stained lipids were imaged under an inverted microscope and quantified spectrophotometrically at 510 nm after isopropanol extraction. Total RNA was isolated using Trizol reagent, and cDNA was synthesized from 2.0  μg RNA. RT-qPCR was performed using an ABI 7300 system to quantify expression of FXR, SHP, cholesterol 7-alpha hydroxylase (CYP7A1), sterol regulatory element binding protein-1 (SREBP-1c), peroxisome proliferator-activated receptor alpha (PPARα), AMPK, and glucose-regulated protein 78 (GRP78). Primers were provided by Beijing Tsingke Biotech Co., Ltd (China). Additionally, intracellular cyclic AMP (cAMP) levels in HepG2 cells were quantified using a competitive ELISA kit (Jiangsu Meimian Industrial Co., Ltd., Jiangsu, China) following the manufacturer’s instructions, with concentrations normalized to total protein content.

### 2.13. Molecular dynamics simulation analysis

The initial binding modes of UDCA to both human and mouse GLP-1R were predicted via molecular docking using AutoDock Vina, following standard preparation of the respective proteins (hydrogen addition) and the ligand (Gasteiger charge assignment and energy minimization). Molecular dynamics simulations of the docked UDCA-GLP-1R complexes (both human and mouse variants) were performed using GROMACS v2022.03^40^ with the AMBER99SB-ILDN force field. Ligand topologies were generated using the General AMBER Force Field (GAFF) via AmberTools22, incorporating Restrained Electrostatic Potential (RESP) charges derived from quantum mechanical calculations in Gaussian 16 W and processed following established wavefunction analysis protocols.^41^^,^^42^ Each complex was solvated in a cubic box using the TIP3P water model with a 1.2 nm margin and neutralized to a physiological state with 0.154 M NaCl. Stepwise equilibration was conducted for each system: a 100 ps NVT ensemble at 300 K regulated by the V-rescale thermostat, followed by a 100 ps NPT ensemble at 1.0 bar using the Berendsen barostat. Finally, a 200 ns production MD simulation was carried out for each complex. During the simulations, the LINCS algorithm was applied to constrain hydrogen bonds. Electrostatic interactions were computed using the Particle Mesh Ewald method for long-range effects and a 1.0 nm cutoff for short-range interactions.

Post-simulation trajectory analyses, including root mean square deviation (RMSD), root mean square fluctuation (RMSF), radius of gyration (Rg), solvent-accessible surface area (SASA), and hydrogen bond occupancy, were performed. To evaluate conformational stability, Gibbs free energy landscapes (FEL) were constructed using the g_sham tool. Additionally, the quantitative binding free energies of the complexes were estimated using the MM/PBSA method via MMPBSA.py v16.0.

### 2.14. Surface plasmon resonance (SPR) analysis

To experimentally validate the direct binding affinity between UDCA and GLP-1R, SPR experiments were performed using a Biacore 8 K system (Cytiva) equipped with a CM5 sensor chip. The sensor surface was activated by injecting a freshly prepared 1:1 mixture of 400  mM *N*-ethyl-N’-(3-dimethylaminopropyl) carbodiimide (EDC) and 100  mM *N*-hydroxysuccinimide (NHS) at a flow rate of 10 μL/min for 420 s. Recombinant mouse GLP-1R protein (Catalog No. HY-P72206, MedChemExpress) was diluted in 10 mM sodium acetate buffer (pH 4.5) to a concentration of 20 μg/mL and then immobilized onto the sample channel (Fc2) at 10 μL/min, achieving an immobilization level of approximately 12,600 response units (RU). The reference channel (Fc1) was left untreated as a control. Following immobilization, unreacted active esters on the surface were blocked with 1 M ethanolamine hydrochloride (pH 8.5) for 420 s at 10 μL/min. For kinetic analysis, the analyte UDCA was serially diluted in the running buffer (1 × PBS containing 1% DMSO and 0.005% Tween-20) to generate 7 concentrations ranging from 0.39 to 25.00  μM. Each concentration was injected sequentially over both channels at a flow rate of 20 μL/min, with an association phase of 100 s and a dissociation phase of 180 s. The sensor chip was regenerated after each cycle. All binding data were processed and fitted to a 1:1 Langmuir binding model using the Biacore Insight Evaluation Software (Cytiva).

### 2.15. Statistical analysis

Statistics were performed using R (version 4.2.2). The continuous variables were expressed as means  ±  standard deviation (SD). ANOVA and Tukey’s post-hoc test were used to determine differences among multiple groups, and Student’s t-test was applied to evaluate 2-group differences in biochemical indicators, genes, and protein expressions. A *p*-value < 0.05 was considered statistically significant. We applied the Bray-Curtis-based principal coordinate analysis (PCoA, R package “vegan” and “ggplot2”), and principal component analysis (PCA, R package “FactoMineR” and “factoextra”) to assess the differences in gut microbiome and metabolites among groups. Changing patterns in gut microbiota or ileum contents’ responses to different interventions were assessed using the fuzzy C-means clustering (R package “ClusterGVis”).^43^^,^^44^

## 3. Results

### 3.1. *L. salivarius* has high BSH production capacity

We included 6 representative bacterial genomes from our in-house library, encompassing clinically relevant probiotic species. These were *Bifidobacterium animalis* XA-768, *Bifidobacterium adolescentis* XA-1069, *Ligilactobacillus salivarius* XA-1416, *Lactiplantibacillus plantarum* XA-314, *Bifidobacterium longum* XA-1102, and *Bifidobacterium longum subsp. Suillum* XA-7822.

*In silico* analysis initially identified *Lactiplantibacillus plantarum* XA-314 as harboring the highest bsh gene copy number, followed by *L. salivarius* XA1416 (Tables S3 and S4). We further examined key genes involved in bile acid metabolism, including the *Bai operon* and those encoding 3α*-HSDH*, 3β*-HSDH*, 7α*-HSDH*, 7β*-HSDH*, and 12α*-HSDH.*^45^ Notably, XA-314 harbored *baiE*, *baiA2*, 3β-*HSDH*, and 7α-*HSDH*, while *L. salivarius* XA1416 exclusively contained *baiA2* and 7α-*HSDH*, which were absent in other strains (Table S4). These genes were uniquely identified through structure-based protein-language model prediction.

To determine the potent candidate, we evaluated their enzymatic binding capacities and phenotypic metabolic diversity. Affinity predictions revealed that one specific *bsh* copy of XA-1416-2 (Plas1_91) possessed the absolute highest binding affinity among all assessed copies from the six strains, especially for glycine-conjugated bile salt substrates as well as for bile acid products (Figure 1A-B). This copy has 98% similarity to the *bsh* gene of *L. salivarius* XA-1416 based on the AA sequence (Table S5). Meanwhile, one copy of XA-314-3 (Contig_13_chromosome_98) also showed very high affinity with a similar binding preference as XA-1416-2. This copy shared 99% similarity to the *bsh* gene of *Lactiplantibacillus plantarum* based on the AA sequence (Table S5). Moreover, *in vitro* assays confirmed robust BSH activity in *L. salivarius* XA1416, particularly in deconjugating tauro-bile salts (Figure S1A-C).

Crucially, to assess the ultimate functional outcome of these BSH activities, we performed metabolomic profiling, which demonstrated that *L. salivarius* XA1416 produced the most diverse bile acid pool among tested strains, as reflected by the highest Shannon index (Figure 1C). These findings underscore the exceptional bile acid metabolism capacity of *L. salivarius* XA1416, suggesting profound implications for modulating intestinal homeostasis.

Collectively, integrated *in silico* and *in vitro* screening identified *L. salivarius XA1416* as exhibiting the highest BSH activity, prompting its selection for further characterization. *L. salivarius* XA1416 was consequently chosen for further analysis.

### 3.2. *L. salivarius* exhibits robust acid tolerance and colonization capacity

To function as an effective probiotic, the gastrointestinal tolerance and sustained intestinal colonization of *L. salivarius* XA1416 were assessed. *In vitro* analysis demonstrated high gastric acid resistance of this strain, with 93.3% viability retention after 0.5 h in simulated gastric fluid (pH 2.5) and > 75% survival over 2 h (Figure 1D). The strain also displayed notable intestinal fluid tolerance, maintaining 60% viability after 3 h in the simulated intestinal fluid.

*In vivo* colonization investigations (Figure 1E) revealed a rapid accumulation of *L. salivarius* XA1416 in the mouse intestine after oral administration. After 2-week gavage, the strain persisted in feces for up to 5 d before gradually declining (Figure S1D). To further elucidate the spatial distribution and persistence in the gut, we quantified its absolute copy numbers in key segments, i.e., jejunum, ileum, cecum, and colon. Specifically, *L. salivarius* XA1416 exhibited a highly specific spatial colonization, robustly localizing in the lower gastrointestinal tract, following the 2-week gavage. The highest accumulation was clearly observed in the cecum (Figure 1H, with a total of 2.15 × 10^7 copies in DNA) and colon (Figure 1I, with a total of 1.26 × 10^7 copies in DNA). In contrast, its levels in the jejunum and ileum were nearly undetectable (Figure 1F, G). At 7 d post-cessation, its absolute abundance in all segments declined to near-baseline levels. These findings indicate that *L. salivarius* XA1416 could successfully colonize in the distal gut with the primary anatomical sites^46^ where microbial BSHs function and where GLP-1-secreting L-cells are located.^47^ Moreover, its subsequent clearance by the host’s natural colonization resistance suggests a high safety profile, minimizing the risk of long-term disruption to the native microbiome.^48^^,^^49^ Additionally, we evaluated its presence in human feces after consumption, (Figure 1J). *L. salivarius* XA1416 showed consistent accumulation, particularly in overweight individuals (Figure 1K). In normal-weight subjects, its detectability in feces displayed notable inter-individual variability.

### 3.3. *L. salivarius* outperforms orlistat in weight control and GLP-1 promotion

**Figure 2.**
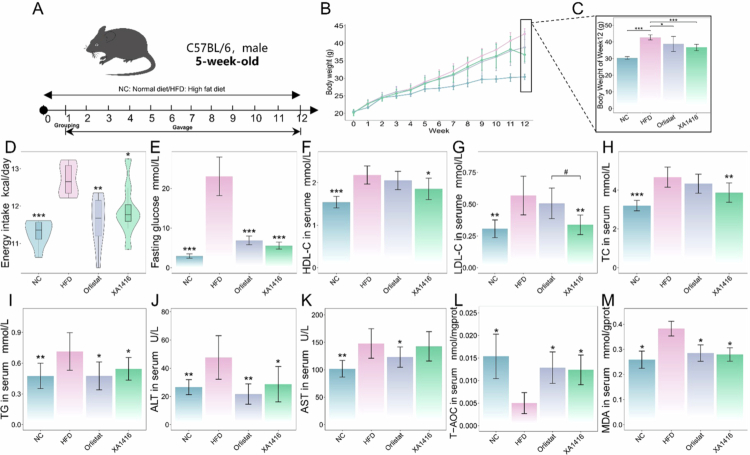
Anti-obesity of *L. salivarius* XA1416. (A) Animal study design. (B) Body weight changes among interventions. (C) Final body weight. (D) Energy intake. (E) Fasting glucose. The levels of (F) high-density lipid cholesterol (HDL-C), (G) low-density lipid cholesterol (LDL-C), (H) total cholesterol (TC), (I) triglycerides (TG), (J) alanine aminotransferase (ALT), (K) aspartate aminotransferase (AST), (L) total antioxidant capacity (T-AOC) and (J) malondialdehyde (MDA) in serum. NC: a normal control group (*n* = 8), HFD: a high-fat diet group (*n* = 8), Orlistat: a group administered with orlistat by gavage (10 mg/kg, *n* = 8), *L. salivarius* XA1416: a group administered with *L. salivarius* XA1416 by gavage (2 × 10^8^ CFU/mouse, *n* = 8). Student’s t-test to evaluate differences between two groups. *p < 0.05; **p < 0.01; ***p < 0.001 vs. HFD group, ^#^*p* < 0.05 (*L. salivarius* XA1416 vs. Orlistat).

We investigated the anti-obesity effect of *L. salivarius* XA1416 on mice fed on a high-fat diet (Figure 2A). Compared to NC, HFD-fed mice showed a 40.4% increase in body weight (Figure 2B–C), driven by elevated energy intake (Figure 2D) and subsequent elevation of fasting blood glucose (Figure 2E). HFD also exhibited higher serum levels of LDL-C, TC, TG, ALT, AST, and MDA (Figure 2H–K, M), along with reduced HDL-C and T-AOC levels (Figure 2F, L). Notably, *L. salivarius* XA1416 effectively attenuated HFD-induced abnormal weight gain by 16.2%, comparable to the anti-obesity of orlistat, and significantly ameliorated these metabolic abnormalities, demonstrating efficacy comparable to orlistat in modulating adiposity and improving glycemic and lipid profiles.

**Figure 3.**
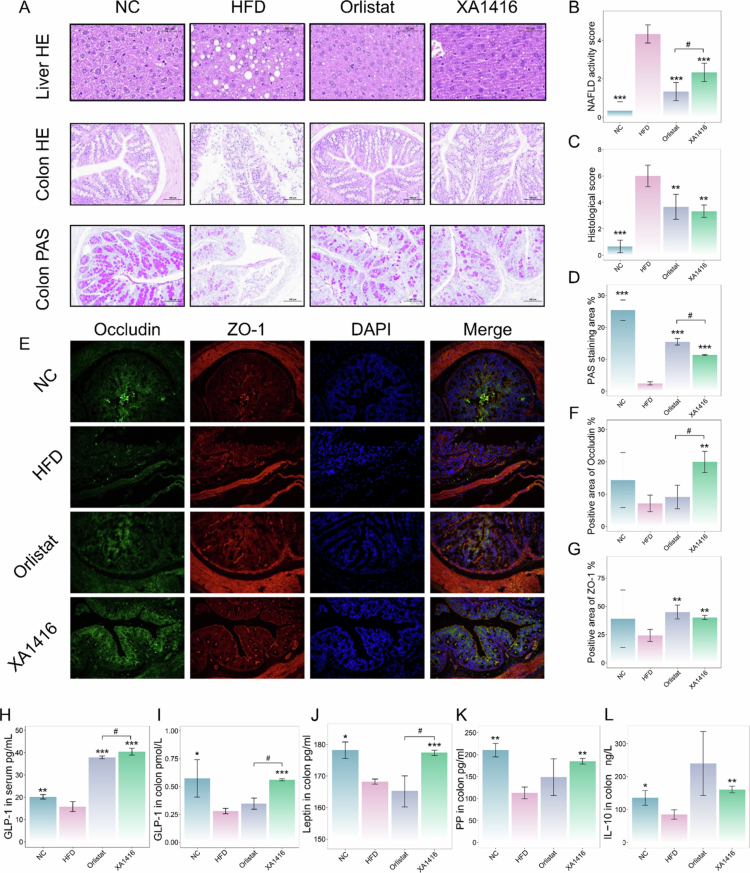
Alleviating effects of *L. salivarius* XA1416 on liver and intestinal damage of mice fed on a high-fat diet. (A) Histological sections of liver tissue and colon tissue. (B) The NAFLD activity score of the liver tissue. (C) Histological score of the colon tissue. (D) Quantitative analysis of PAS staining area (%) in the colon. (E–G) The fluorescent images of ZO-1 (red) and Occludin (green). Scale bar, 100 μm. *n* = 4 mice per group. *L. salivarius* XA1416 promoted the secretion of gut hormones (H–I) GLP-1, (J) leptin, (K) PP, and (L) the anti-inflammatory cytokine IL-10. NC: a normal control group (*n* = 8), HFD: a high-fat diet group (*n* = 8), Orlistat: a group administered with orlistat by gavage (10  mg/kg, *n* = 8), *L. salivarius* XA1416: a group administered with *L. salivarius* XA1416 by gavage (2 × 10^8^ CFU/mouse, *n* = 8). Student’s t-test to evaluate differences between two groups. *p < 0.05; **p < 0.01; ***p < 0.001 vs. HFD group, ^#^*p* < 0.05 (*L. salivarius* XA1416. vs. Orlistat).

H&E staining revealed the restorative effects of *L. salivarius* XA1416 and orlistat on HFD-induced abnormal lipid accumulation, inflammatory infiltration, colonic mucosal damage, and disrupted crypt structures in the liver (Figure 3A-D). PAS staining revealed the serious damage of goblet cells in the colon; however, after being orally treated with *L. salivarius* XA1416 or orlistat, these trends were significantly reversed. Histological analysis showed that *L. salivarius* XA1416 demonstrated superior efficacy in HFD-induced colon damage than orlistat (Figure 3E-F). Moreover, immunofluorescence staining indicated that *L. salivarius* XA1416 increased colonic tight junction proteins. Compared to NC, colonic expressions of occludin and ZO-1 were significantly reduced in HFD. Both *L. salivarius* XA1416 and orlistat restored their expression to varying degrees, with *L. salivarius* XA1416 showing a stronger effect in upregulating occludin (Figure 3G-I).

Most notably, serum concentration of GLP-1 was significantly lower in HFD mice relative to NC (Figure 3J). This reduction was strongly reversed by administration with *L. salivarius* XA1416 (*p* = 4.45712E-09). Colonic concentrations of key gut hormones, GLP-1, leptin, and PP, were markedly reduced in HFD mice by 51%, 5%, and 49%, respectively, compared to the NC group. *L. salivarius* XA1416 administration more effectively restored these hormonal levels than orlistat, increasing GLP-1 by 49%, fully normalizing leptin concentrations (100% recovery), and elevating PP by 35% (Figure 3K–M). In addition, HFD mice exhibited a significant suppression of the anti-inflammatory cytokine IL-10 (*p* = 0.03056198 vs. NC), which was markedly upregulated upon *L. salivarius* XA1416 (*p* = 0.001648433, Figure 3N).

### 3.4. *L. salivarius* restores the disorder of bile acid metabolism in mice fed on HFD

**Figure 4.**
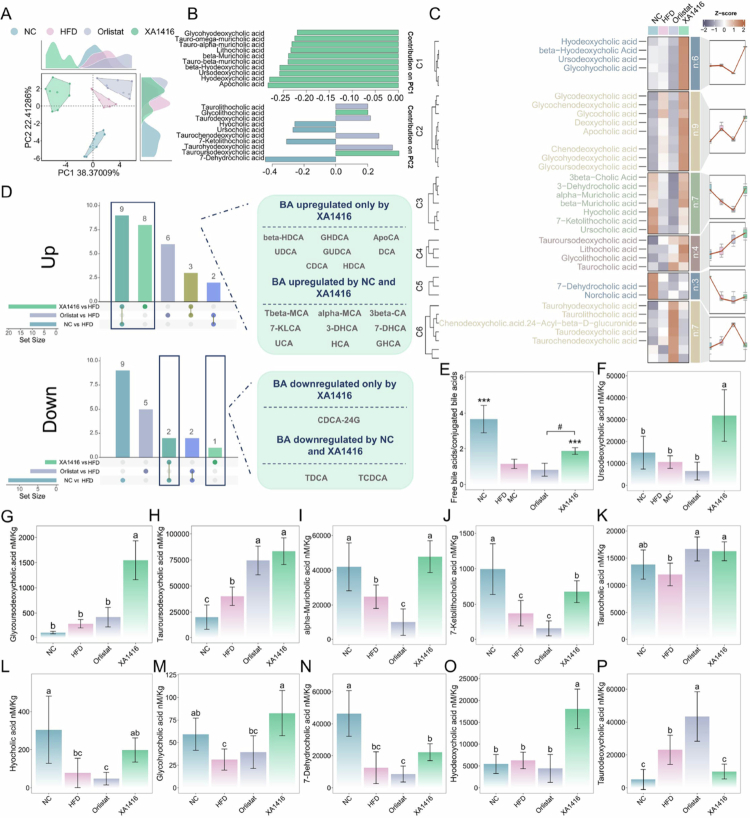
Effects of *L. salivarius* XA1416 on bile acid profiles in the ileum. (A, B) Differences in bile acid profiles illustrated by PCA analysis. (C) Distinct effects of *L. salivarius* XA1416 on ileum contents’ metabolites derived by using the fuzzy C-means clustering algorithm. *n* = 6 mice per group. (D) Upset Venn plots illustrating the differential bile acid profiles of mice with various intervention modalities. (E) Ratio of free bile acids to conjugated bile acids. The levels of (F) ursodeoxycholic acid, (G) glycoursodeoxycholic acid, (H) tauroursodeoxycholic acid, (I) alpha-Muricholic acid, (J) 7-Ketolithocholic acid, (K) taurocholic.acid, (L) hyocholic acid, (M) glycohyodeoxycholic acid, (N) 7-Dehydrocholic acid, (O) hyodeoxycholic acid, and (P) taurodeoxycholic acid. Differences between multiple groups were assessed by ANOVA, followed by Tukey’s post hoc test. Significance was indicated by different letters (*p* < 0.05). Student’s t-test was used to evaluate differences between two groups. *p < 0.05; **p < 0.01; ***p < 0.001 vs. HFD group.

We conducted targeted LC-MS metabolomics to assess bile acid profiles (*n* = 45) in the ileal contents of mice across interventions. PCA analysis on bile acids revealed clear segregation among groups (Figure 4A, B). *L. salivarius* XA1416 exhibited a distinct clustering pattern along PC1, which was predominantly associated with a reduction in apocholic acid (ApoCA) relative to HFD. This shift corresponded to significant alterations in the levels of ApoCA, hyodeoxycholic acid (HDCA), ursodeoxycholic acid (UDCA), and glycohyodeoxycholic acid (GHDCA). Separation along PC2 was primarily driven by the NC group, characterized by elevated concentrations of 7-dehydrocholic acid (7-DHCA), 7-ketolithocholic acid (7-KLCA), ursocholic acid (UCA), and hyocholic acid (HCA). In contrast, tauroursodeoxycholic acid (TUDCA) and taurohyodeoxycholic acid (THDCA) were notably enriched in both *L. salivarius* XA1416 and Orlistatorlistat groups.

To characterize treatment-specific effects on bile acid profiles, we applied fuzzy C-means clustering, revealing six distinct patterns of bile acids (Figure 4C, Table S6). Specifically, bile acids in cluster 3 (*n* = 7) and 5 (*n* = 3) were remarkably suppressed by HFD compared with NC, whereas *L. salivarius* XA1416 not only restored bile acid levels belonging to cluster 3 but also uniquely elevated those in clusters 1 and 2, particularly UDCA, chenodeoxycholic acid (CDCA), glycohyocholic acid (GHCA), and glycoursodeoxycholic acid (GUDCA). Notably, although orlistat failed to restore HFD-induced disturbances as effectively as *L. salivarius* XA1416, it distinctly elevated bile acids in cluster 6 (*n* = 7), particularly THDCA, taurocholic acid (TCA), taurodeoxycholic acid (TDCA), and taurochenodeoxycholic acid (TCDCA).

When assessing individually (Figure 4D), comparative analysis showed that compared to HFD, *L. salivarius* XA1416 uniquely upregulated 8 bile acids (*β*-HDCA, GHDCA, ApoCA, UDCA, GUDCA, deoxycholic acid (DCA), CDCA, and HDCA), while sharing 9 elevated bile acids with NC and 3 with Orlistat. Both *L. salivarius* XA1416 and NC downregulated TDCA and TCDCA relative to HFD, with *L. salivarius* XA1416 specifically modulating CDCA-24G.

BSH activity mediates the deconjugation of conjugated bile acids in the intestine.^50^^,^^51^ Consistent with this, we found that the free-to-conjugated bile acid ratio, a known indicator of BSH activity, was significantly reduced in the HFD group compared to NC (*p* < 0.001). *L. salivarius* XA1416 restored this ratio (*p* < 0.001; Figure 4E) and markedly increased the abundance of bile acids implicated in weight control, hepatointestinal protection, and GLP-1 secretion (Figure 4F–P), in particular, UDCA (increased by 233.0%), GUDCA (increased by 451.3%), and TUDCA (increased by 107.4%). In contrast, *L. salivarius* XA1416 exclusively reduced TDCA.

### 3.5. *L. salivarius* modulates the fecal microbiome

**Figure 5.**
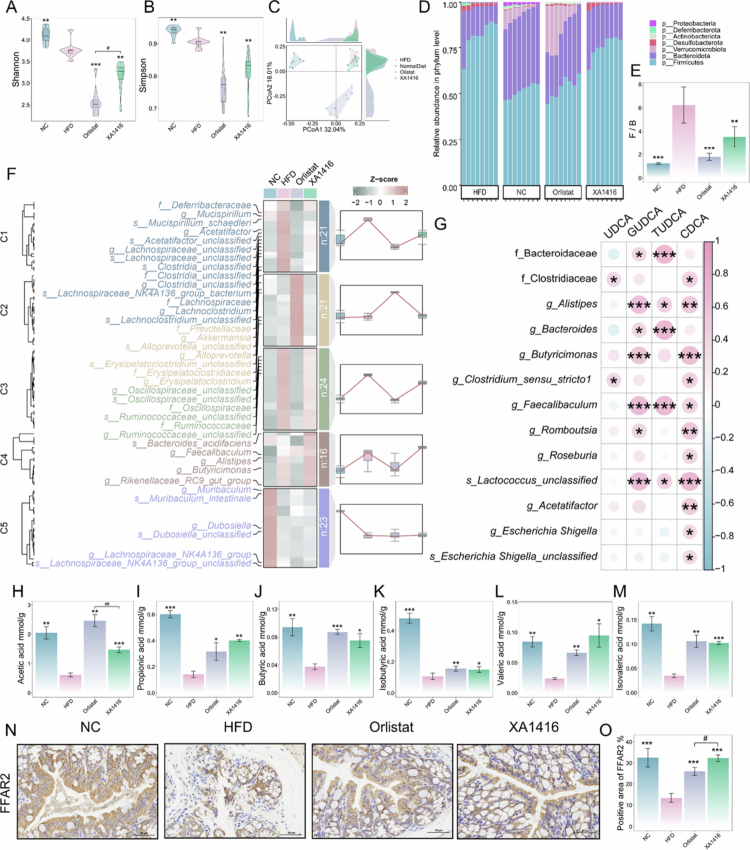
Effects of *L. salivarius* XA1416 on gut microbiota compositions. *α*-Diversity was presented as the (A) Shannon and (B) Simpson indices. (C) PCoA analysis (D) Percent of community abundance at the phylum level. (E) Ratio of Firmicutes to Bacteroidetes. (F) Distinct effects of *L. salivarius* XA1416 on gut microbiota disorders in HFD mice (including family level, genus level, and species level) were derived by using the fuzzy C-means clustering algorithm. *n* = 8 mice per group. (G) Correlation analysis of key bile acids with the gut microbiota regulated by *L. salivarius* XA1416. The levels of (H) acetic acid, (I) propionic acid, (J) butyric acid, (K) isobutyric acid, (L) valeric acid, and (M) isovaleric acid. (N) IHC images of FFAR2. Scale bar, 100 μm. *n* = 4 mice per group. (O) Positive area of FFAR2. Student’s t-test was used to evaluate differences between two groups. *p < 0.05; **p < 0.01; ***p < 0.001 vs. HFD group, ^#^*p* < 0.05 (*L. salivarius* XA1416. vs. Orlistat).

To characterize gut microbiota composition across experimental groups, we performed 16S rRNA sequencing on fecal samples. Compared with NC, HFD showed a slight reduction in *α*-diversity (Shannon and Simpson indices), whereas *L. salivarius* XA1416 intervention significantly decreased both indices (Figure 5A, B). To determine whether this reduction in diversity reflected shifts in pathogenic or commensal bacteria, we analyzed microbial composition in depth. While PCoA revealed distinct clustering among the NC, Orlistat, and HFD groups, *L. salivarius* XA1416 did not significantly alter overall microbiota structure compared to HFD at the community level (Figure 5C). However, phylum-level analysis demonstrated compositional changes. Although Bacteroidetes and Firmicutes dominated all groups, HFD exhibited a significantly higher Firmicutes-to-Bacteroidetes ratio than NC (*p* < 0.001). This obesity associated gut dysbiosis was attenuated by both *L. salivarius* XA1416 and orlistat (*p* < 0.001 and *p* < 0.01, respectively; Figure 5D, E).

Clustering gut microbiota revealed 5 distinct patterns of response to the treatments (Figure 5F, Table S7). Specifically, compared with the NC, HFD increased abundances of 45 taxa grouped within cluster 1 and 3, e.g., f_Deferribacteraceae, *g_Mucispirillum*, *g_Acetatifactor*, *g_Lachnoclostridium*, f_Oscillospiraceae, and f_Ruminococcaceae. Conversely, HFD reduced several bacteria grouped in clusters 2 and 5, including f_Prevotallaceae, *g_Akkermansia*, *g_Alloprevotella*, *g_Erysipelatoclostridium*, *g_Muribaculum*, *g_Dubosiella*, and *g_Lachnospiraceae_*NK4A136 (Figure S1A–G). *L. salivarius* XA1416 alleviated HFD-induced gut dysbacteriosis, leading to a microbiota composition resembling that of the NC group. Additionally, *L. salivarius* XA1416 exhibited a better enhancement effect on the gut microbiota, particularly when compared to HFD. This effect was most pronounced in bacteria taxa associated with cluster 4, including *g_Faecalibaculum*, *g_Alistipes*, and *g_Butyricimonas* (Figure S1H–J).

Strong links were demonstrated between *L. salivarius* XA1416-modulated bacterial taxa and bile acids (Figure 5G, S1K–P). For instance, *g_Bacteroides*, *g_Alistipes*, *g_Faecalibaculum,* and *s_Lactococcus_unclassified* were significantly positively correlated with GUDCA, CDCA, and GHDCA. Especially noteworthy is the markedly positive correlation between f_Clostridiaceae, *g_Clostridium_sensu_stricto1*, CDCA, and UDCA observed in these mice (*p* < 0.01).

Additionally, elevated levels of SCFAs are implicated as key contributors to the maintenance of gut homeostasis (symbiosis), in contrast to the dysbiotic state.^52^ As expected, HFD reduced fecal concentrations of acetic, butyric, propionic, isobutyric, valeric, and isovaleric acids compared to NC (Figure 5H–M). Both *L. salivarius* XA1416 and Orlistat restored SCFAs production. Moreover, *L. salivarius* XA1416 enhanced colonic expression of FFAR2, a G protein-coupled receptor activated by microbial-derived SCFAs such as butyrate. These findings suggest that *L. salivarius* XA1416 augments SCFA-mediated signaling pathways, potentially reinforcing intestinal symbiosis and metabolic health.

### 3.6. Clinical implications of *L. salivarius*-enriched UDCA on obesity prevention

Given that *L. salivarius* XA1416 altered the bile acids implicated in metabolic regulation, particularly increasing the abundance of UDCA, CDCA (the precursor of UDCA), and its products GUDCA and TUDCA, we employed two-sample MR analysis and *in vitro* fecal microbiota fermentation to infer causality, complemented by two cross-sectional study analyses to explore associations and support our findings.

**Figure 6.**
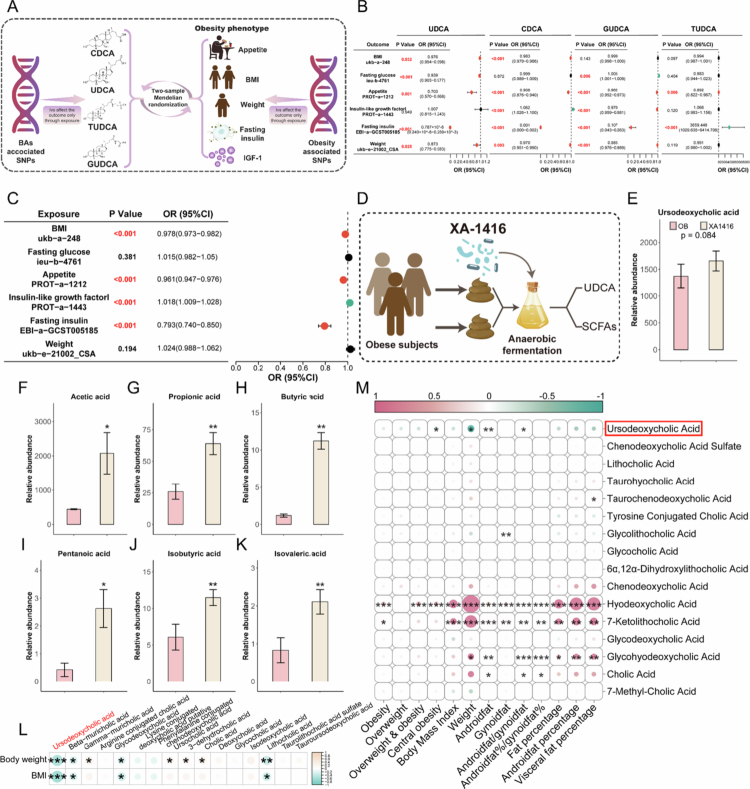
The key role of UDCA is involved in the *L. salivarius* XA1416’s anti-obesity effects. (A) Schematic representation of the two-sample Mendelian randomization analysis. (B, C) Causal associations between UDCA and obesity phenotypes. (D) The *in vitro* fecal microbiota fermentation study design. (E) The abundance of UDCA in XA1416-modulated fecal fermentation broth. The levels of (F) acetic acid, (G) propionic acid, (H) butyric acid, (I) isobutyric acid, (J) valeric acid, and (K) isovaleric acid. (L) Analysis of fecal bile acids in relation to body weight and BMI in a cross-sectional cohort (*n* = 60). (M) Analysis of the correlation between 16 BAs and obesity-related indicators in a cross-sectional cohort (*n* = 539).

Bidirectional two-sample MR analyses established causal relationships between four bile acids, i.e., UDCA, CDCA, GUDCA, and TUDCA, that were exclusively enriched by *L. salivarius* XA1416 and obesity-related metabolic traits (Figure 6A). Genetically predicted levels of UDCA were inversely associated with elevated body mass index (BMI) (*β* = −0.025, 95% CI: [0.954–0.998], *p* = 0.032), fasting insulin (*β* = −14.055, 95% CI: [2.389 × 10^-9-0.000259], *p* = 2.013 × 10^-6), appetite (*β* = −0.352, 95% CI: [0.570–0.868], *p* = 0.001), and body weight (*β* = −0.136, 95% CI: [0.775–0.983], *p* = 0.025). CDCA, GUDCA, and TUDCA showed directionally consistent but weaker effects on appetite, weight, and fasting glucose relative to UDCA. With the exception of GUDCA, all bile acids were positively associated with insulin-like growth factor-1 (IGF-1), a hormone implicated in metabolic regulation^53^ (Figure 6B, Table S8-9). Reverse MR analyses further supported a causal effect of higher obesity-related traits on reduced bile acid levels, with the most pronounced and consistent influence observed for UDCA (Figure 6C, Table S10, 11).

To further validate the functional impact of *L. salivarius* XA1416 on UDCA, we conducted *in vitro* fermentation experiments, revealing that *L. salivarius* XA1416 could directly upregulate UDCA (*p* = 0.084) in the fecal microbiota of obese people with BMI > 28 (Figure 6D-E). Moreover, *L. salivarius* XA1416 also caused higher concentrations of SCFAs in the same fecal samples (Figure 6F–K). Furthermore, in a cross-sectional cohort of 60 participants, we observed significant inverse associations between several bile acids, including UDCA, beta-muricholic acid, GDCA, and lithocholic acid, and both BMI and body weight (Figure 6L). This finding was further supported and generalized in a larger cross-sectional cohort (*n* = 539). We profiled 16 fecal bile acids in this population and assessed their associations with obesity and its indicators (Figure 6M). Among several microbiota-derived secondary bile acids, UDCA exhibited robust inverse associations with body weight, abdominal fat content, and the prevalence of central obesity, after adjusting for lifestyle confounders including age, gender, education, smoking status, and altitude level. downstream metabolite targeted and enriched by *L. salivarius* XA1416.

Collectively, these results from both *in vitro* fermentation and human studies establish UDCA-mediated signaling as the principal mechanism through which *L. salivarius* XA1416 exerts its metabolic benefits, corroborating our findings from animal models.

### 3.7. *L. salivarius-*enriched UDCA prevents weight gain while enhancing GLP-1

**Figure 7.**
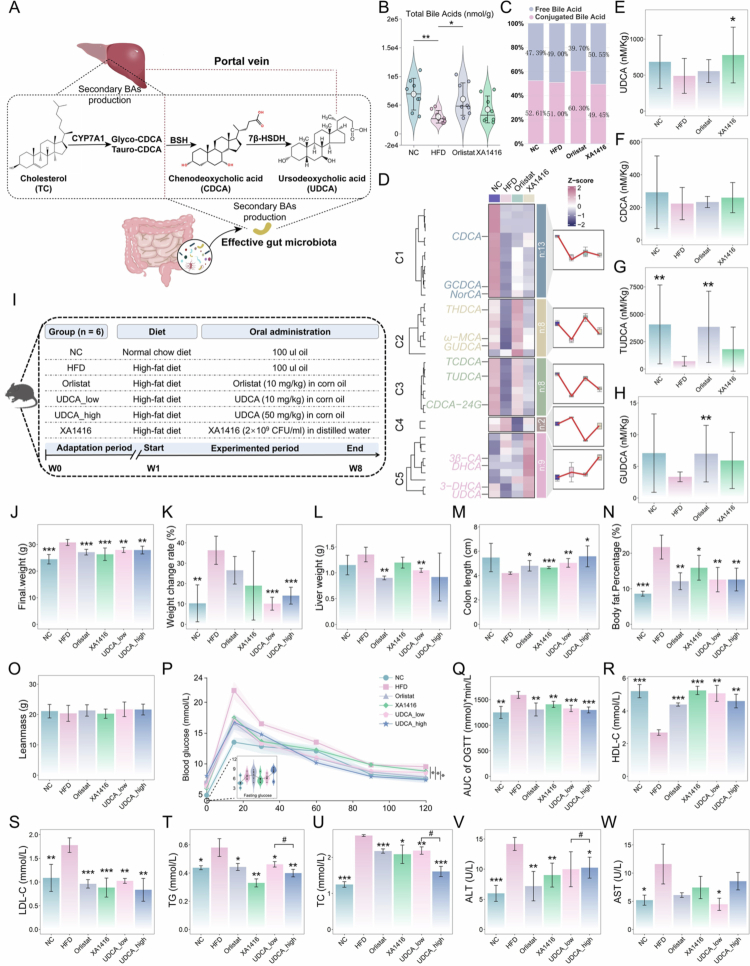
Anti-obesity effects of UDCA on high-fat diet-induced adiposity and associated metabolic disorders. (A) Schematic diagram of UDCA origin. (B) Total hepatic bile acid concentrations. (C) Proportions of free and conjugated hepatic bile acids. (D) Distinct effects of *L. salivarius* XA1416 on liver BAs derived by using the fuzzy C-means clustering algorithm. *n* = 8 mice per group. Hepatic concentrations of (E) CDCA, and the core secondary effector molecules (F) UDCA, (G) TUDCA, and (H) GUDCA. (I) The grouping and flowchart of the mice experiment. Differences in (J) body weight, (K) weight change rate, (L) liver weight, (M) colon length, (N) body fat percentage, (O) leanmass, (P) oral glucose tolerance test and (Q) the area under the curve, (R) high-density lipid cholesterol (HDL-C), (S) low-density lipid cholesterol (LDL-C), (T) total triglycerides (TG), (U) total cholesterol (TC), (V) alanine aminotransferase (ALT), and (W) aspartate aminotransferase (AST) in serum between groups. NC (normal diet, *n* = 7), HFD (high-fat diet, *n* = 7), Orlistat (HFD and oral gavage of Orlistat (2  mg/mL in corn oil), *n* = 7), *L. salivarius* XA1416 (HFD and oral gavage of 2 × 10^8^ CFU/mouce XA1416, *n* = 5), UDCA_low (HFD and oral gavage of UDCA (2 mg/mL in corn oil), *n* = 7), UDCA_high (HFD and oral gavage of UDCA(10 mg/mL in corn oil), *n* = 7). Student’s t-test was used to evaluate differences between two groups. *p < 0.05; **p < 0.01; ***p < 0.001, ^#^*p* < 0.05 (UDCA_low.vs.UDCA_high).

Clinical implications of *L. salivarius*-enriched UDCA on obesity prevention prompted us to further investigate its anti-obesity potential via a gut-liver axis in mouse models. UDCA, an intestinal FXR antagonist, is a secondary bile acid whose biosynthesis relies on gut microbiota. It is synthesized from CDCA via microbial 7β-hydroxysteroid dehydrogenase. UDCA biosynthesis begins with the hepatic conversion of cholesterol to CDCA, mediated by CYP7A1. In the gut, bacterial BSHs (especially from strains with high BSH activity) deconjugate CDCA, which is then transformed into UDCA via microbial 7β-epimerization. The newly formed UDCA is subsequently reabsorbed into the enterohepatic circulation to help maintain bile acid homeostasis (Figure 7A).

To investigate the impact on hepatic bile acid metabolism, we performed targeted quantification of 40 bile acid species in liver tissues from mice in the NC, HFD, Orlistat, and XA1416 groups. Compared with the NC group, HFD feeding significantly disrupted both the total hepatic bile acid pool and the balance between conjugated and free bile acids. *L. salivarius* XA1416 reversed these alterations (Figure 7B-C). To gain a more comprehensive view, we generated a trend clustering model based on the 40 quantified bile acids, which revealed five distinct patterns in response to treatments (Figure 7D; Table S12). Bile acids in clusters 1 (*n* = 13), 2 (*n* = 8), and 3 (*n* = 8) were markedly suppressed by HFD relative to NC. In contrast, XA1416 uniquely elevated several species, including UDCA (Figure E, 59.30% increase vs. HFD, *p* = 0.049) and its primary precursor chenodeoxycholic acid (CDCA, Figure 7F, XA1416 vs HFD, *p* = 0.228), as well as 3β-CA, dehydrocholic acid (DHCA), and 3-DHCA. Moreover, TUDCA and GUDCA also showed trends toward enrichment in the liver, although the differences did not reach statistical significance (Figure 7G-H, *p* = 0.086 and *p* = 0.073, respectively). These results collectively indicate that *L. salivarius* XA1416 exerts a profound effect on bile acid profiles, and UDCA might be the primary effector mediating the anti-obesity effects of XA1416.

To determine the anti-obesity capacity of UDCA, we administered low (10 mg/kg) and high (50 mg/kg) doses to HFD-fed mice for 8 weeks (Figure 7I). The Food and Drug Administration (FDA)-approved human dose for chronic liver diseases is 13–15 mg/kg/day, which has a well-established safety profile. Given that mice typically require higher mg/kg doses owing to faster metabolic rates, a 50 mg/kg dose was, therefore, also included to ensure efficacy.

Here, we found that a daily oral dose of 2 × 10^8^ CFU per mouse achieved UDCA concentrations comparable to those attained with low-dose pharmacological UDCA (10 mg/kg per day). UDCA treatment effectively attenuated HFD-induced weight gain, reducing both body weight and liver weight (Figure 7J–L), while preventing colon shortening (Figure 7M). UDCA, *L. salivarius* XA1416, and orlistat reversed the HFD-induced increases in body fat percentage, with UDCA showing superior efficacy (UDCA_low reduced by 42.12% and UDCA_high reduced by 42.04%, Figure 7N). The lean mass remained unchanged across all groups (Figure 7O). This finding shows that the body fat-reduction efficacy of UDCA and *L. salivarius* XA1416.

**Figure 8.**
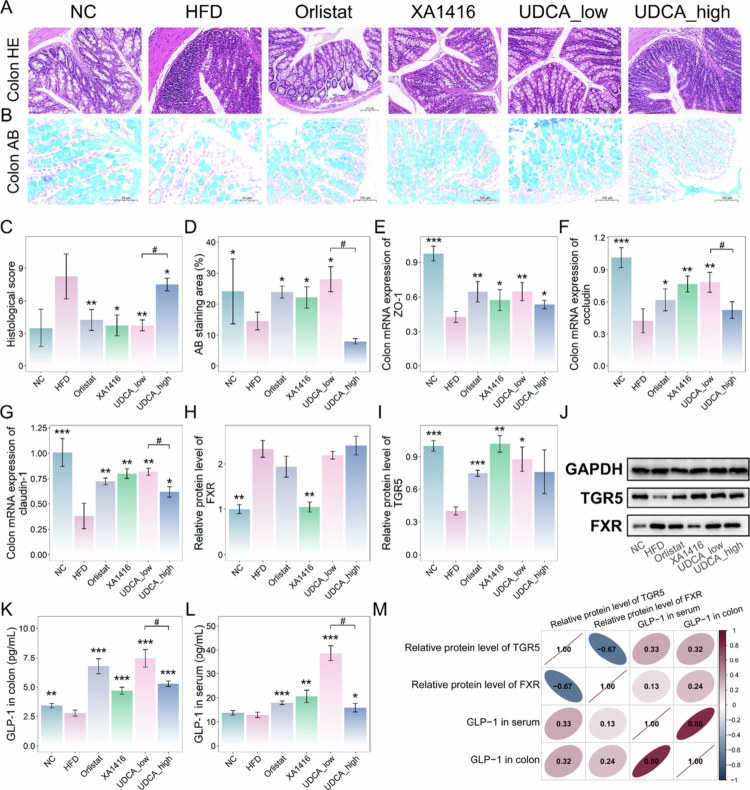
Alleviating effects of UDCA on colon injury and its modulating effects on TGR5 and FXR signaling. (A–B) Images of colon tissue’s H&E and Alcian blue staining. Scale bar, 100 μm. *n* = 4 mice per group. (C) Histological score. (D) AB staining area. Relative mRNA expressions of (E) ZO-1, (F) occluding, and (G) claudin-1 in the colonic tissue. Protein expressions of (H) FXR and (I) TGR5. (J) Western blot analysis of TGR5 and FXR in the colonic tissue. (K–L) The levels of GLP-1 in the colon and serum. *n* = 4 mice per group. (M) Correlation analysis of FXR, TGR5, and GLP-1. Student’s t-test was used to evaluate differences between two groups. *p < 0.05; **p < 0.01; ***p < 0.001, ^#^*p* < 0.05 (UDCA_low.vs.UDCA_high).

Moreover, UDCA, *L. salivarius* XA1416, and Orlistat improved glucose tolerance (Figure 7P, Q) and HDL-C in HFD-fed mice (Figure 7R). Concurrently, we observed significant reductions in LDL-C, TG, TC, and liver enzymes ALT and AST (Figure 7S–W), indicating comprehensive metabolic benefits. Meanwhile, histological analysis revealed that Orlistat, *L. salivarius* XA1416, and UDCA attenuated HFD-induced colonic inflammation and mucus layer depletion in HFD mice (Figure 8A–D). HFD markedly downregulated intestinal barrier genes (i.e., ZO-1, occludin, and claudin-1) compared to NC (Figure 8E–G). Notably, orlistat, *L. salivarius* XA1416, and UDCA treatments restored HFD-impaired colonic histology, mitigating inflammation and improving intestinal permeability, with low-dose UDCA demonstrating optimal therapeutic effects. By contrast, high-dose UDCA failed to induce significant body weight reduction, meanwhile, exacerbated mucosal damage, as evidenced by the expression of tight junction proteins.

Importantly, emerging evidence shows that mechanistic links between bile acids and metabolic homeostasis may involve two major bile acid-sensing receptors, FXR and TGR5,^54^ which also influence secretion of GLP-1 and glucose homeostasis.^55^^,^^56^ Here, to investigate UDCA’s role, we assessed mRNA and protein expression of these receptors in the colon and measured GLP-1 levels in the colon and serum. Specifically, HFD increased TGR5 while suppressing FXR levels (Figure 8H-J) and GLP-1 (Figure 8K-M). Treatment with UDCA, XA1416, or orlistat restored FXR, TGR5, and GLP-1 expression. The low-dose UDCA exhibited the most pronounced effect, significantly elevating GLP-1 by 168.4% and 199.9% in colon and serum, respectively, relative to HFD mice (*p* = 5.64714E-05, *p* = 1.41722E-05).

### 3.8. UDCA promotes bile acid balance and hepatic lipid homeostasis

Given the established role of the gut-liver axis in coordinating bile acid homeostasis and lipid metabolism, we next investigated whether *L. salivarius* XA1416-enriched UDCA influences hepatic FXR/SHP signaling to maintain metabolic balance. As a key regulator of bile acid synthesis and cholesterol catabolism, hepatic FXR activation modulates downstream pathways critical for lipid homeostasis. We therefore examined the effects of UDCA treatment on hepatic gene expressions and metabolic regulators in HFD-fed mice.

**Figure 9.**
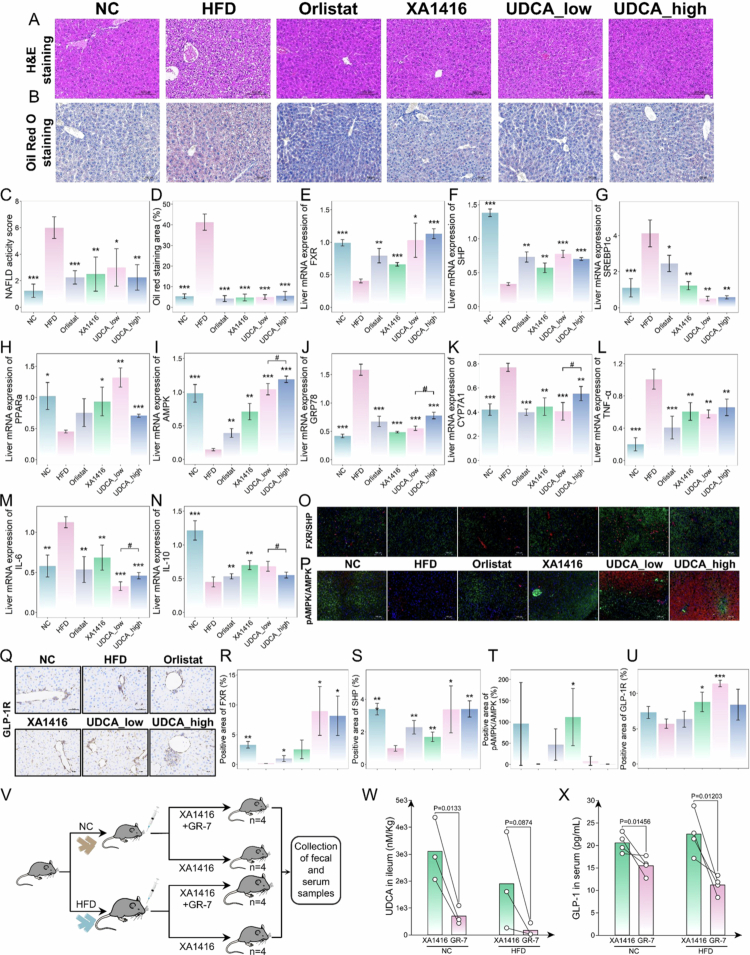
Alleviating effects of UDCA on hepatic functions through expressing BSH. Images of liver tissue (A) H&E and (B) Oil Red O staining. Scale bar, 100 μm. *n* = 4 mice per group. (C) The NAFLD activity score (NAS) of the liver tissue. (D) Oil Red O-stained area. The mRNA levels of (E) FXR, (F) SHP, (G) SREBP-1c, (H) PPARα, (I) AMPK, (J) GRP78, (K) CYP7A1, (L) TNF-*α*, (M) IL-6, and (N) IL-10. *n* = 4 mice per group. (O, P) Representative fluorescent images of SHP/FXR and AMPK/p-AMPK. (Q) IHC images of GLP-1R. Scale bar, 100 μm. *n* = 4 mice per group. (R–U) Positive area of FXR, SHP, AMPK/p-AMPK and GLP-1R. (V) The flowchart of the BSH inhibitor treatment experiment. (W) Concentration of UDCA in the ileum. *n* = 3 mice per group. (X) Concentration of GLP-1 in the serum. *n* = 4 mice per group. Student’s t-test to evaluate differences between two groups. *p < 0.05; **p < 0.01; ***p < 0.001, ^#^*p* < 0.05 (UDCA_low vs. UDCA_high).

Histopathological results reveal that all UDCA, *L. salivarius* XA1416, and orlistat treatments protected HFD-induced abnormal accumulations in lipid droplets and fat vacuoles of liver samples, with a lower nonalcoholic fatty liver disease (NAFLD) score than HFD (Figure 9A-D). As a clinically used FXR agonist in primary biliary cholangitis, UDCA potently activates hepatic FXR, thereby inducing bile acid synthesis enzymes and accelerating cholesterol conversion into bile acids, which ultimately reduces serum cholesterol levels. Therefore, we next explored whether UDCA treatment activates hepatic FXR and how FXR affects liver pathology and the underlying mechanism linking the two organs (the intestine and liver). Our result showed that UDCA, *L. salivarius* XA1416 and orlistat dramatically increased the hepatic mRNA expression of FXR (Figure 9E), SHP (Figure 9F), PPARα (Figure 9H), AMPK (Figure 9I) and IL-10 (Figure 9M), while inhibiting SREBP-1c (Figure 9G), GRP78 (Figure 9J), CYP7A1 (Figure 9K), TNF-*α* (Figure 9L) and IL-6 (Figure 9M) in mice fed on HFD, in line with the immunofluorescence results (Figure 9O, P, R–T). Notably, HFD downregulated GLP-1R, a G protein-coupled receptor critical for hepatic glucose and lipid metabolism, whereas a robust elevation was observed in UDCA_low, *L. salivarius* XA1416, and Orlistat groups (Figure 9Q, U). Collectively, UDCA demonstrated superior efficacy to *L. salivarius* XA1416 and Orlistat in activating hepatic FXR/SHP signaling, thereby improving lipid metabolism.

Furthermore, we found that with administration of the specific BSH inhibitor GR-7, *L. salivarius* XA1416 failed to increase fecal concentration of UDCA as well as serum GLP-1 (Figure 9W and X), consistently confirming that *L. salivarius* XA1416 exerts its anti-obesity effects by producing UDCA through BSH-mediated mechanisms.

### 3.9. Hepatic protection effect of UDCA *in* HepG2 cell models

**Figure 10.**
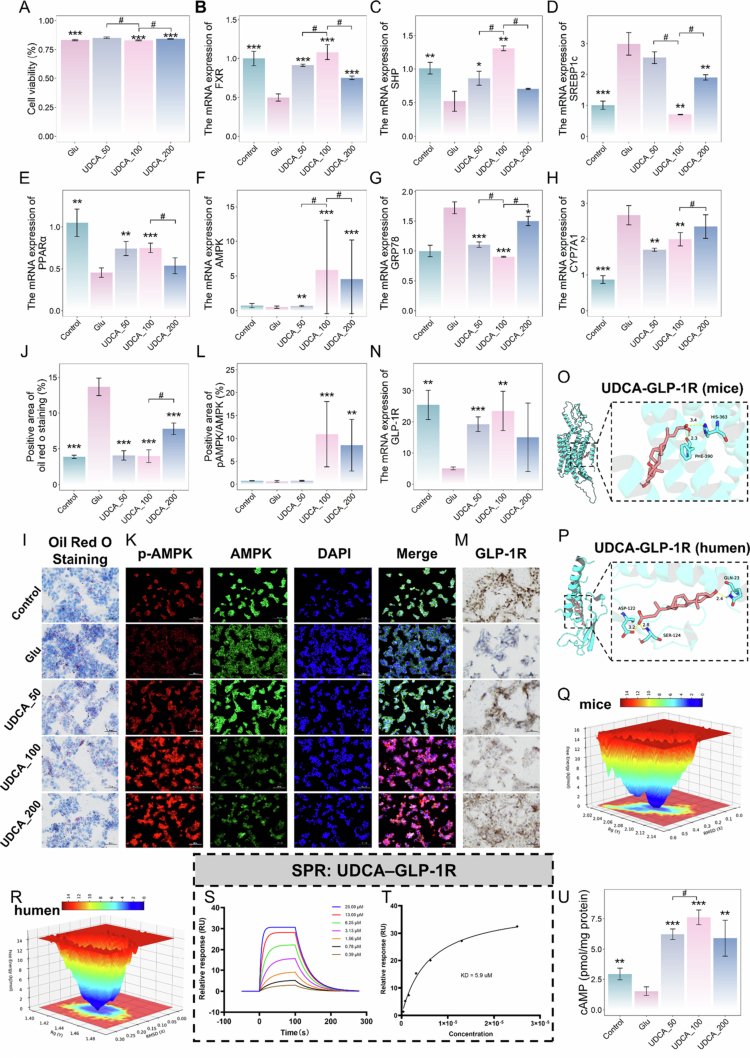
UDCA reverses abnormalities in bile acid metabolism and dyslipidemia in glucose-treated HepG2 cells. (A) The viability of HepG2 cells treated with high glucose after different interventions. The mRNA levels of (B) FXR, (C) SHP, (D) SREBP-1c, (E) PPARα, (F) AMPK, (G) GRP78, and (H) CYP7A1. (I) Oil Red O-stained slices of HepG2 cells are shown. Scale bar, 50 μm. *n* = 4 mice per group. (J) Quantitative analyses of Oil Red O-stained slices of HepG2 cells are shown. (K) Representative fluorescent images of AMPK/p-AMPK. Scale bar, 100 μm. *n* = 4 mice per group. (L) Positive area of AMPK/p-AMPK. (M) Immunofluorescence staining of GLP-1R in HepG2 cells. Scale bar, 50 μm. *n* = 4 mice per group. (N) Positive area of GLP-1R. Molecular docking results of the (O) mouse and (P) human complexes over 200  ns MD simulations. Gibbs free energy of the (Q) mice and (R) humans. (S) Surface plasmon resonance (SPR) kinetic sensorgrams of UDCA binding to immobilized GLP-1R. (T) Steady-state affinity fitting curve. (U) Concentration of cAMP in HepG2 cells. *n* = 4 mice per group. Student’s t-test is used to evaluate differences between two groups. *p < 0.05; **p < 0.01; ***p < 0.001, ^#^*p* < 0.05 (UDCA_50. vs. UDCA_100; UDCA_200. vs. UDCA_100).

To further substantiate the dose-dependent effects of UDCA, we treated high glucose-exposed HepG2 cells with UDCA at concentrations of 50, 100, and 200  μM. High glucose significantly inhibited the viability of HepG2 cells (Figure 10A), while treatment with UDCA demonstrated a significant mitigation of the viability. The Oil Red O staining results consistently demonstrated the efficacy of UDCA in mitigating high glucose-induced abnormal lipid accumulation (Figure 10I–J). As expected, the mRNA expression of FXR, along with its downstream targets SHP, was upregulated under the UDCA intervention (Figure 10B–C), consequently inhibiting the mRNA expression of the downstream cytokine CYP7A1, SREBP-1c, and GRP78 (Figure 10D, G-H), while promoting the mRNA expression of PPARα and enhancing AMPK phosphorylation (Figure 10E, F, K-L).

Notably, IHC staining revealed significantly reduced GLP-1R protein levels in glucose-treated HepG2 cells (Glu) compared to Control (*p* = 0.003006194), while UDCA treatment effectively restored GLP-1R expression (Figure 10M-N). UDCA_100 exhibited the most pronounced effect in regulating indicators involved in bile acid metabolism and glucolipid metabolism, further confirming its superior anti-obesity potential over the other two doses.

### 3.10. Functional activation of GLP-1R by UDCA

The GLP-1 receptor, a member of the G protein-coupled receptor (GPCR) family,^57^ exhibits specific affinity for GLP-1 and plays a central role in hepatic glucolipid metabolism. Herein, i*n vivo* and cell experiments consistently indicated that UDCA not only enhances GLP-1 secretion but also upregulates hepatic GLP-1R expression. To explore whether UDCA may exert direct agonistic activity on GLP-1R, we employed molecular dynamics simulations to evaluate the structural binding potential between UDCA and this receptor.

Specifically, robust spontaneous interactions between UDCA and both mouse and human GLP-1R were observed. For the mouse GLP-1R, the binding energy was calculated as -8.417 kcal/mol. The 2D interaction profile indicates that UDCA is primarily accommodated within a deep hydrophobic pocket, engaging in extensive hydrophobic contacts with residues including Leu7, Leu8, Leu10, Ala11, Leu14, Ile147, Ile357, Pro358, Phe390, Phe393, and Met397. These interactions are further reinforced by van der Waals forces with adjacent polar residues such as Ser389, Thr391, His363, and Gln394 (Figure 10O). Docking of UDCA with human GLP-1R yielded a binding energy of −7.822 kcal/mol. UDCA forms key hydrogen bonds with the side chain of Ser124 and the backbone of Asp122. These polar contacts are further stabilized by a network of hydrophobic interactions involving Ile29, Ala30, Val33, Trp91, and Leu123, as well as electrostatic and van der Waals contacts with surrounding charged residues (Figure 10P).

To evaluate the dynamic stability of the GLP-1R–UDCA complexes, 200 ns molecular dynamics simulations were conducted. Both mouse and human complexes achieved stable trajectories, as indicated by consistently low RMSD for protein backbones (mouse: ~0.35–0.45 nm; human: ~0.20–0.25 nm) and ligands (mouse: ~0.15–0.20 nm; human: ~0.05–0.20 nm), suggesting firm binding without significant displacement inside the binding pocket (Figure S3C–D). Structural compactness, reflected by Rg (mouse: ~2.05–2.10 nm; human: ~1.45 nm) and SASA (mouse: 155–175 nm²; human: 68–74 nm²), was maintained throughout (Figure S3E–H). DSSP confirmed the integrity of both receptors, with stable *α*-helical content (mouse: ~200–220 residues; human: ~15–25 residues) (Figure S3I–J). RMSF values, indicating residue flexibility, were mostly below 0.4 nm for the mouse complex, with higher flexibility observed only at the terminal and loop regions, and mostly below 0.3 nm for the human complex (Figure S3K-L). Intermolecular hydrogen bonds between UDCA and the receptors fluctuated between 0 and 4 for mouse, and 0 to 3 for human, supporting stable binding interactions (Figure S3M-*N*).

To visualize conformational stability, two-dimensional Gibbs free energy landscapes (FEL) were constructed. The mouse complex exhibited a single deep energy well centered at RMSD 0.25–0.35 nm and Rg 2.06–2.10 nm, indicating a dominant stable conformation (Figure 10Q). Similarly, the human complex showed its global energy minimum at RMSD 0.10–0.15 nm and Rg 1.43–1.45 nm (Figure 10R). MM-PBSA calculations further confirmed strong binding, with total free energies of –18.0 kcal/mol (mouse) and –17.0 kcal/mol (human). In both cases, van der Waals forces (–44.0 and –38.0  kcal/mol, respectively) were the primary contributors, supported by electrostatic and non-polar solvation terms (Figure S3O–P).

To substantiate the direct physical interaction predicted by our *in-silico* models, we subsequently performed a SPR assay. Real-time kinetic analysis demonstrated a robust, dose-dependent binding response upon the injection of varying concentrations of UDCA (0.39 to 25.00  μM) to the immobilized GLP-1R protein. Fitting the sensorgrams to a 1:1 Langmuir binding model yielded excellent fitted curves (Figure 10S) with Ka of 6.47 × 10³ 1/Ms and Kd of 2.87 × 10^−2^ 1/s. Consistent with the kinetic evaluation, steady-state affinity analysis further corroborated this strong interaction, producing an equilibrium dissociation constant (KD) of 5.9  μM (Figure 10T).

Furthermore, GLP-1R is a classical GPCR that couples to Gαs, leading to the activation of adenylate cyclase and the subsequent production of intracellular cAMP. Consistent with our *in-silico* binding data, UDCA significantly and dose-dependently increased intracellular cAMP levels in HepG2 cells compared to the Glucose group (Figure 10U, *p* < 0.01). Collectively, these findings provide compelling evidence that UDCA acts not merely as a structural binder, but as a functional agonist of GLP-1R, triggering the downstream signaling cascades that influence hepatic homeostasis.

## 4. Discussion

**Figure 11.**
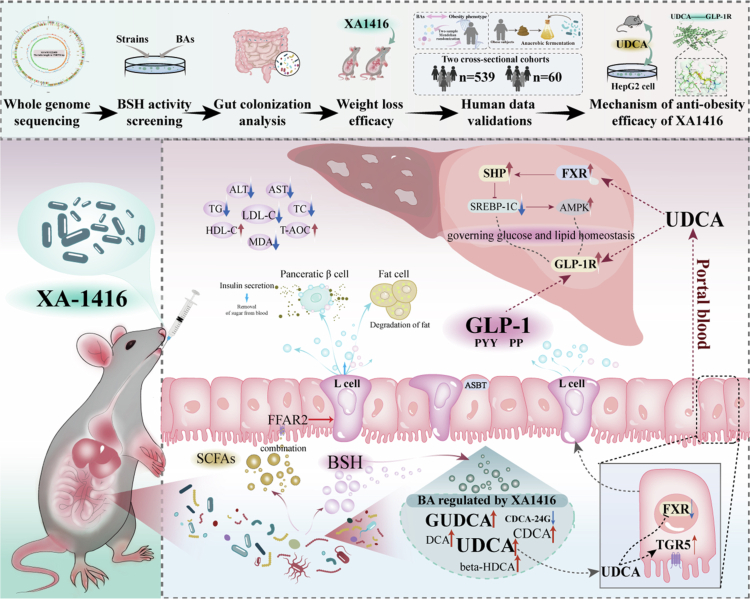
Summary of the bile acids-GLP-1 mediated mechanism underlying the anti-obesity effect of *L. salivarius* XA1416.

We identified *L. salivarius* XA1416, a novel probiotic strain isolated from healthy human feces that exhibits high BSH activity, robust gastric acid resistance, and efficient gut colonization, supporting its potential for long-term obesity management. Compared to orlistat, *L. salivarius* XA1416 demonstrated comparable efficacy in reducing obesity, while more effectively enhancing GLP-1 secretion and restoring metabolic homeostasis. Such benefits were mediated specifically by UDCA, a microbiota-derived bile acid enriched by *L. salivarius* XA1416. UDCA increased intestinal and circulating GLP-1, while concurrently activating the hepatic GLP-1R to improve lipid metabolism and bile acid homeostasis. Associations between UDCA and improved obesity phenotypes were demonstrated by using MR analysis of human GWAS data, two cross-sectional studies, and *in vitro* fecal fermentation models. Using HepG2 cell models and molecular dynamics simulations, we provided novel information on the direct activating effect of UDCA on GLP-1R. Our study demonstrates *L. salivarius* XA1416 as an effective anti-obesity probiotic, with UDCA as a key microbial metabolite mediating its metabolic benefits. Findings also highlight BSH-targeted probiotics therapy as a viable strategy for combating obesity and metabolic diseases (Figure 11).

A high-fat diet is a primary driver of increased obesity, elevating the risk of metabolic disorders.^58-60^ Emerging evidence underscores the endocrine function of bile acids as critical regulators of metabolic homeostasis, mediated through receptors such as FXR and TGR5,^21^^,^^61^^,^^62^ affirming the therapeutic potential of targeting bile acid signaling in the development of novel anti-obesity strategies. Notably, microbial BSH activity plays a fundamental role in bile acid metabolism by regulating the deconjugation of conjugated bile acids, fundamentally reshaping the host bile acid pool.^17^^,^^18^ This biochemical transformation underscores the metabolic influence of BSH-producing genera, i.e., *Bacteroides, Clostridium, Lactobacillus,* and *Bifidobacterium*, in metabolic regulation and obesity through bile acid signaling. For instance, elevated abundances of BSH-active *L. murinus* and *L. reuteri* have been correlated with decreased body weight and improved glucose regulation in humans.^13^ *L. plantarum* strains (Lp91 and Lp21) exhibited potent BSH activity, exhibiting a bile acid amino conjugate glycocholate, enabling efficient deconjugation of glycocholate, along with cholesterol assimilation and co-precipitation *in vitro.*^63^ Guo et al. revealed that symbiotic probiotics, specifically *Lactobacillus* and *Bifidobacterium* strains with high BSH activity, modulated bile acid metabolism and alleviated HFD-induced metabolic disorders.^64^

Here, leveraging genomic screening technology, we uncovered a BSH-producing probiotic *L. salivarius* XA1416 with pronounced gastric acid tolerance and intestinal colonization that potently stimulates intestinal GLP-1 secretion, demonstrating anti-obesity efficacy comparable to orlistat, with strong potential for clinical translation. While prior studies have documented the anti-inflammatory and gut barrier-strengthening properties of *L. salivarius,*^65^^,^^66^ its specific role in modulating obesity and related metabolic syndromes has remained poorly understood. Our work now delineates a novel microbial metabolite-GLP-1 mechanism through which *L. salivarius* XA1416 exerts anti-obesity effects, thereby advancing the mechanistic landscape of probiotic therapeutics and positioning this strain as a promising candidate for obesity interventions.

Specifically, *L. salivarius* XA1416 reshaped gut microbiota, marked by a selective enrichment of key commensal genera including *Clostridium, Bacteroidaceae, Alistipes, Butyricmonas,* and *Faecalibaculum,* alongside the reinforcement of intestinal barrier integrity, remarkable increases in intestinal CDCA, UDCA, GUDCA, and TUDCA, and a significant elevation in intestinal and circulating GLP-1 levels. Our *in vivo* spatial tracking revealed that *L. salivarius* XA1416 preferentially and robustly colonizes the distal gut in mice, specifically the cecum and colon. This specific anatomical engraftment provides a profound physiological advantage for metabolic regulation. Enteroendocrine L-cells are widely distributed along the gastrointestinal tract, but stereological and immunohistochemical studies confirm that their absolute density and total cell numbers peak dramatically in the distal colon and rectum. By localizing precisely where L-cell density is highest,^67^ our strain holds great potential to ensure its secreted microbial metabolites (specifically UDCA and SCFAs) achieve maximal local concentrations in the immediate vicinity of their target receptors. This anatomical synergy allows UDCA to potently engage basolateral TGR5 and FXR, while SCFAs concurrently activate free fatty acid receptors (e.g., FFAR2), which maximizes intestinal GLP-1 secretion. Importantly, MR analysis using large-scale human GWAS data provided robust genetic evidence supporting a causal role of elevated UDCA in reducing obesity risk. This approach, leveraging genetic variants as instrumental variables, minimizes confounding and reverse causation, thereby extending and complementing findings from animal models. Despite CDCA’s favorable effects on BMI and body weight in MR analysis, substantial evidence indicates that CDCA may cause adverse effects, including hepatotoxicity and diarrhea, thereby limiting its clinical utility.^68^ In contrast, UDCA has an established safety profile as an FDA-approved drug for cholestatic liver diseases.

Furthermore, we observed an inverse correlation between fecal UDCA levels and body mass index in a human cross-sectional cohort. To directly probe microbial metabolic activity, we used an *in vitro* fecal fermentation experiment inoculated with obese donor microbiota, which demonstrated that *L. salivarius* XA1416 effectively promotes microbial production of UDCA and SCFAs. This model enables direct assessment of bacterial function, underscoring the specific and direct metabolic influence of *L. salivarius* XA1416. Together, these integrated human genetic, clinical, and mechanistic data establish UDCA as a key mediator of the anti-obesity effects of *L. salivarius* XA1416.

Previous studies demonstrate that UDCA ameliorated hepatic steatosis and enhanced insulin sensitivity of obese mice.^69^^,^^70^ Moving beyond its conventional applications, this study reveals that UDCA enriched by *L. salivarius* XA1416 recapitulated the anti-obesity effects and metabolic benefits of this strain, in particular, enhancing intestinal and circulating GLP-1. As an endogenous FXR antagonist, UDCA potently stimulates GLP-1 secretion by alleviating FXR-mediated suppression of glycolysis and ChREBP-driven proglucagon gene transcription in enteroendocrine L-cells.^55^^,^^56^^,^^71^^,^^72^ Meanwhile, UDCA functions as a potent agonist of TGR5, a receptor highly expressed on the basolateral membrane of enteroendocrine L-cells. TGR5 activation has been shown to elevate cAMP, enhancing mitochondrial oxidative phosphorylation, while inhibiting ATP-sensitive K⁺ channels, leading to membrane depolarization and subsequent activation of voltage-gated calcium channels. Calcium influx then triggers the exocytosis of secretory granules containing GLP-1, thereby promoting hormone secretion.^71^^,^^73^^,^^74^ This coordinated signaling through nuclear and membrane bile acid receptors synergistically promotes GLP-1 production and secretion, proving UDCA as a multifaceted modulator of enteroendocrine function.

Most interestingly, we noted the great potential of UDCA in activating hepatic GLP-1 receptors, which, to our knowledge, has not been previously elucidated. GLP-1R is functionally expressed in hepatocytes, where it orchestrates key pathways governing glucose and lipid homeostasis. Hepatic GLP-1R activation enhances insulin sensitivity, suppresses de novo lipogenesis, and inhibits fatty acid uptake, key mechanisms implicated in obesity and metabolic disorders.^75-77^ GLP-1R agonists, such as liraglutide, semaglutide, and beinaglutide, have received approval from both the FDA and the National Medical Products Administration of China for treating obesity and are widely and increasingly used. Here, we observed a notable activation of hepatic GLP-1R by UDCA, initiating a suppression of SHP/SREBP-1c signaling. This leads to downregulation of lipogenic genes (e.g., GRP78, CYP7A1) and upregulation of PPARα and AMPK, collectively improving lipid metabolism and bile acid homeostasis. Interestingly, the subsequent molecular dynamics simulation analysis provided a dynamic and atomic-level perspective, confirming a strong and robust theoretical binding capacity between UDCA and GLP-1R. This computational approach quantified the interaction’s stability over time and calculated the binding free energy using the MM/PBSA method. The highly negative binding free energy value provided conclusive thermodynamic evidence for the affinity of UDCA, supporting it as a promising candidate for modulating GLP-1R activity, which offers a novel mechanistic perspective for targeting obesity and associated metabolic diseases. To definitively validate this direct physical interaction, we utilized SPR, which provided real-time biophysical evidence of UDCA binding directly to the GLP-1R with an equilibrium dissociation constant of 5.9  μM. Because physical occupancy alone does not guarantee functional agonism, we further evaluated intracellular signaling. The robust, dose-dependent elevation of intracellular cAMP observed in UDCA-treated HepG2 cells conclusively proves that UDCA acts as a functional agonist of the Gαs -coupled GLP-1R. This cAMP surge triggers downstream cascades that ultimately propel mitochondrial fatty acid *β*-oxidation and improve hepatic lipid homeostasis.^78^

Beyond the UDCA–GLP-1 axis, we noticed that *L. salivarius* XA1416 significantly restored SCFAs production, thereby activating FFAR2–GLP-1 signaling and ameliorating HFD-induced metabolic dysfunction. Acetate and propionate, key SCFAs elevated by XA1416, are known to activate FFAR2 on intestinal L-cells, directly promoting GLP-1 secretion and linking microbial fermentation to host metabolic homeostasis.^79^ Butyrate has also been shown to enhance hepatic sensitivity to GLP-1 through FFAR2-mediated upregulation of its receptor, and its conversion to sodium butyrate reinforces this effect,^80^ thereby amplifying GLP-1-dependent regulation of bile acid and lipid metabolism, in line with our findings. Concurrently, colonic SCFAs lower luminal pH, modulating the activity of the gut microbial enzyme 7β-HSDH, which catalyzes the conversion of CDCA to UDCA. UDCA, in turn, together with *L. salivarius* XA1416, selectively enriches SCFA-producing bacteria such as Oscillospiraceae and Ruminococcaceae,^81^ establishing a positive feedback loop that sustains SCFAs production and consolidates a metabolically favorable microenvironment, which could propagate anti-obesity effects of *L. salivarius* XA1416.

A key strength of this study is the discovery and mechanistic elucidation of *L. salivarius* XA1416 as a novel probiotic strain that stably colonizes the gut and is capable of promoting GLP-1 secretion and ameliorating obesity via a gut-liver crosstalk, identifying UDCA as a mediator of the effect. *L. salivarius* XA1416 exhibits anti-obesity efficacy comparable to orlistat, yet without inducing common adverse effects often observed for orlistat, such as steatorrhea or fecal incontinence.^82^ Critically, *L. salivarius* XA1416 elevated UDCA, an FDA-approved drug with a well-established safety profile for the treatment of chronic liver conditions in humans(13-15 mg/kg per day).^83^ Here, we found that a daily oral dose of 2 × 10^9^ CFU per mouse achieved UDCA concentrations comparable to those attained with low-dose pharmacological UDCA (10 mg/kg per day). These results further support that *L. salivarius* XA1416 is a safe, sustainable, and potentially long-term alternative to conventional drug regimens for the management of obesity and associated metabolic disorders.

In conclusion, through high-throughput genomic screening, we identified *L. salivarius* XA1416, a novel anti-obesity strain exhibiting high BSH activity, acid resistance, and efficient gut colonization. This strain confers robust protection against diet-induced obesity by modulating bile acid metabolism and enhancing GLP-1 secretion. We further demonstrate that *L. salivarius* XA1416-elevated UDCA recapitulated these anti-obesity and metabolic benefits through a gut-liver axis. Our study suggests *L. salivarius* XA1416 as a translatable probiotic candidate for obesity mitigation, with UDCA as a key effector molecule mediating its therapeutic benefits.

## Supplementary Material

Supplementary Material source data 1.xlsx

Supplementary Material source data 2.xlsx

Supplementary Material Supplemental material_XA1416_20260320LJY.docx

## Data Availability

All data relevant to the study were included in the article or uploaded as supplementary information. The accession number for the 16S rRNA sequencing dataset of the animal study reported in this manuscript is NCBl BioProject (PRINA1072268), along with the whole-genome sequencing data of the strain (PRJNA1359115). For any additional information needed to reanalyze the reported data, please contact the lead authors: Lin Shi (linshi198808@snnu.edu.cn), Yan Tan (yant@xbiome.com), and Wen Peng (wen.peng2014@foxmail.com). GWAS data of bile acids and obesity phenotypes are provided as source data files (source data 1 and 2) accompanying this paper.
