# Supplementary material for: A novel bile salt hydrolase-producing Ligilactobacillus salivarius prevents diet-induced obesity via regulation of bile acid metabolism and glucagon-like peptide 1 restoration
Source: Gut Microbes. 2026 May 6;18(1):2668127. doi: 10.1080/19490976.2026.2668127 (PMC13166280; doi:10.1080/19490976.2026.2668127)
Supplement: Supplementary Material — Supplemental material_XA1416_20260320LJY.docx [file KGMI_A_2668127_SM6078.docx]

**A novel bile salt hydrolase -producing *Ligilactobacillus salivarius* prevents diet-induced obesity via regulation of bile acid metabolism and glucagon-like peptide 1** **restoration**

Jiayao Lv ^a, #^, Lanqi Zhou ^b, #^, Xiaoshuang Dai ^c, #^, Rikard Landberg ^d^, Huicui Meng ^e^, Honglei Tian ^a^, Shiyi Zhang ^a^, Tianqi Liu ^a^, Xiaochen Yin ^c^, Jiayi Zhang ^a^, Xizi Song ^c^, Christophe Bonny ^c^, Stephanie Blum ^c^, Yousen Cao ^f^, Jingyao Guo ^g^, Wen Peng ^h, i, j, *^, Yan Tan ^c, *^, Lin Shi ^a, *^

^a^ School of Food Engineering and Nutritional Science, Shaanxi Normal University, Xi'an, Shaanxi, 710119, China

^b^ College of food science and technology, Huazhong Agricultural University, Wuhan, 430070, China

^c^ Xbiome, Scientific Research Building, Room 907, Tsinghua High-Tech Park, Shenzhen, 518000, China

^d^ Department of Life Sciences, Food and Nutrition Science, Chalmers University of Technology, Gothenburg, Sweden

^e^ School of Public Health (Shenzhen), Sun Yat-Sen University, Shenzhen, Guangdong, 518107, China

^f^ School of Aerospace Medicine, Fourth Military Medical University, Xi'an 710032, China

^g^ Global Health Institute, School of Public Health, Xi'an Jiaotong University, Xi'an, Shaanxi, 710061, China

^h^ Department of Public Health, Qinghai University Medical College, No. 16 Kunlun Rd, Xining, 810008, China

^i^ Nutrition and Health Promotion Center, Department of Public Health Medical College, Qinghai University, No. 16 Kunlun Rd, Xining, 810008, China

^j^ Qinghai Provincial Key Laboratory of Prevention and Control of Glucolipid Metabolic Diseases with Traditional Chinese Medicine, Medical College, Qinghai University, No. 16 Kunlun Rd, Xining, 810008, China

* Corresponding authors:

Lin Shi, PhD, School of Food Engineering and Nutritional Science, Shaanxi Normal University, Xi’an, Shaanxi 710062, China.

Email: [linshi198808@snnu.edu.cn](mailto:linshi198808@snnu.edu.cn)

ORCID ID: https://orcid.org/0000-0001-9709-3394

Yan Tan, PhD, Xbiome, Scientific Research Building, Room 907, Tsinghua High-Tech Park, Shenzhen, China.

Email: yant@xbiome.com

Wen, Peng MD, MPH, Nutrition and Health Promotion Center, Department of Public Health, Medical College, Qinghai University, Xining, Qinghai 810008, China, No. 16 Kunlun Road, Xining, 810000, China Email: wen.peng2014@foxmail.com

ORCID ID: https://orcid.org/0000-0002-7939-676X

# These authors contributed equally to this work.

**Table of contents**

[Supplemental Tables 1](#_Toc214498966)

[**Table S1.** General genomie properties of 6 experimental strains. 1](#_Toc214498967)

[**Table S2.** All primers used in this study. 2](#_Toc214498968)

[**Table S3.** Linear equation and correlation coefficients of the stndard curves for the bile acid. 1](#_Toc214498969)

[**Table S4.** The distribution of bile acid metabolism genes among the microbial genomes screened. 7](#_Toc214498970)

[**Table S5.** BLASTp and PLMsearch results for bild acid metabolism genes among the selected genomes. 8](#_Toc214498971)

[**Table S6.** Detail information on the 5 distinct clusters of ileal content bile acid metabolome. 10](#_Toc214498972)

[**Table S7.** Detail information on the 6 distinct clusters of gut microbiomes. 12](#_Toc214498973)

[**Table S8.** The estimation of causal effects of bile acids associated with obesity phenotype as outcome. 16](#_Toc214498974)

[**Table S9.** Cochran Q statistic of bile acids to obesity phenotype. 18](#_Toc214498975)

[**Table S10.** The estimation of causal effects of obesity phenotype associated with bile acids as outcome. 21](#_Toc214498976)

[**Table S11.** Cochran Q statistic of obesity phenotype to bile acids. 23](#_Toc214498977)

[**Table S12.** Binding Free Energy of the Complex Calculated by MM/PBSA. 27](#_Toc214498978)

[**Figure S1.** *In-vitro* measurement of BSH activity among the selected microbes. 28](#_Toc214498979)

[**Figure S2.** Relative abundances of microbiotas. 29](#_Toc214498980)

[**Figure S3.** Analysis of molecular docking. 30](#_Toc214498981)

# Supplemental Tables

**Table S1.** General genomie properties of 6 experimental strains.

| **Features** | **XA314** | **XA768** | **XA1069** | **XA1102** | **XA1416** | **XA7822** |
| --- | --- | --- | --- | --- | --- | --- |
| **Genome Size (bp)** | 3,523,401 | 1,935,607 | 2,203,272 | 2,531,254 | 1,942,197 | 2,388,159 |
| **Gene Number** | 3,417 | 1,566 | 1,805 | 2,117 | 1,831 | 2,006 |
| **Total Gene Length (bp)** | 2,961,672 | 1,685,184 | 1,934,706 | 2,198,571 | 1,706,649 | 2,093,379 |
| **Average Gene Length (bp)** | 866.75 | 1076.11 | 1071.86 | 1038.53 | 932.09 | 1043.56 |
| **GC Content (%)** | 44.07% | 60.49% | 59.54% | 59.79% | 33.04% | 60.06% |
| **Gene Density (genes/kb)** | 0.97 | 0.81 | 0.82 | 0.84 | 0.94 | 0.84 |
| **Gene/Genome (%)** | 84.06% | 87.06% | 87.81% | 86.86% | 87.87% | 87.66% |

**Table S2.** All primers used in this study.

| **Genes** | **Forward 5’-3’** | **Reverse 5’-3’** |
| --- | --- | --- |
| **ZO-1** | **GCCGCTAAGAGCACAGCAA** | **TCCCCACTCTGAAAATGAGGA** |
| **Occludin** | **TTGAAAGTCCACCTCCTTACAGA** | **CCGGATAAAAAGAGTACGCTGG** |
| **Claudin-1** | **GGGGACAACATCGTGACCG** | **AGGAGTCGAAGACTTTGCACT** |
| **FXR** | **GCTTGATGTGCTACAAAAGCTG** | **CGTGGTGATGGTTGAATGTCC** |
| **SHP** | **TGGGTCCCAAGGAGTATGC** | **GCTCCAAGACTTCACACAGTG** |
| **CYP7A1** | **GGGATTGCTGTGGTAGTGAGC** | **GGTATGGAATCAACCCGTTGTC** |
| **TNF** | **CCCTCACACTCACAAACCAC** | **ACAAGGTACAACCCATCGGC** |
| **IL-6** | **TGATGGATGCTACCAAACTGGA** | **GTGACTCCAGCTTATCTCTTGG** |
| **IL-10** | **CTGCTATGCTGCCTGCTCTTACTG** | **AGCCGCATCCTGAGGGTCTTC** |
| **GRP78** | **ACTTGGGGACCACCTATTCCT** | **ATCGCCAATCAGACGCTCC** |
| **PPARa** | **AGAGCCCCATCTGTCCTCTC** | **ACTGGTAGTCTGCAAAACCAAA** |
| **AMPK** | **TCTCTATGCTTTGCTGTGTGGAACC** | **GAGGTGGTAGGCGACGGCTAG** |
| **SREBP1c** | **CAGGTGCAGACGGTACAGG** | **CGACCCTTACTGGCACTTGAA** |
| **GADPH** | **GAAGGTGAAGGTCGGAGTCAAC** | **CAGAGTTAAAAGCAGCCCTGGT** |
| ***Ligilactobacillus Salivarius* XA1416** | **GAAACGATGGGAGGATGA** | **TGATAAACTCTGGGCACT** |

**Table S3.** Linear equation and correlation coefficients of the stndard curves for the bile acid.

| Index | Compounds | Class | Q1 (Da) | Q3 (Da) | Molecular Weight | Ionization model | Retaion time | Equation | r | LLOQ | ULOQ | Standard 1 | Standard 2 | Standard 3 |
| --- | --- | --- | --- | --- | --- | --- | --- | --- | --- | --- | --- | --- | --- | --- |
| CDCA | Chenodeoxycholic acid | BAs | 391.3 | 391.3 | 392.292664 | [M-H]- | 10.12 | y = 0.00649 x + 0.00328 | 0.99987 | 20 | 1000 | 11511.52000 | 26368.60000 | 24030.60000 |
| DCA | Deoxycholic acid | BAs | 391.3 | 345.3 | 392.292664 | [M-H]- | 10.25 | y = 0.00153 x + 3.14566e-4 | 0.99988 | 1 | 400 | 15810.28000 | 48232.00000 | 49618.40000 |
| 3-oxo-DCA | 3-oxodeoxycholic acid | BAs | 389.3 | 389.3 | 390.277008 | [M-H]- | 10.27 | y = 0.05352 x + 0.00112 | 0.99966 | 1 | 400 | 20.32540 | 178.63720 | 175.07300 |
| LCA | Lithocholic acid | BAs | 375.3 | 375.3 | 376.29776 | [M-H]- | 11.15 | y = 0.01501 x + 0.03170 | 0.99796 | 2 | 400 | 1.50487 | 9.05770 | 10.90756 |
| DLCA | Dehydrolithocholic acid | BAs | 373.3 | 373.3 | 374.282104 | [M-H]- | 11.23 | y = 0.07427 x + 0.00293 | 0.99791 | 10 | 400 | 0.09702 | 0.11823 | 0.09645 |
| βGCA | 3β-Glycocholic Acid | BAs | 464.309 | 464.309 | 465.309039 | [M-H]- | 1.39 | y = 0.08519 x + 0.00209 | 0.99961 | 4 | 100 | 55.96520 | 13.06088 | 12.54580 |
| GHDCA | Glycohyodeoxycholic Acid | BAs | 448.314 | 448.314 | 449.314124 | [M-H]- | 2.56 | y = 0.18375 x - 2.85820e-4 | 0.99976 | 4 | 100 | 27.23220 | 10.56030 | 11.06182 |
| CA-3S | Cholic Acid 3 Sulfate Sodium Salt | BAs | 487.244 | 487.244 | 488.24439 | [M-H]- | 3.42 | y = 0.00896 x + 8.28904e-4 | 0.99803 | 2 | 100 | 0.50042 | 1.97363 | 2.02826 |
| UCA | Ursocholic acid | BAs | 407.4 | 343.4 | 408.287567 | [M-H]- | 3.49 | y = 8.19076e-4 x - 2.68049e-5 | 0.99946 | 20 | 1000 | 7.32216 | 40.37560 | 38.91120 |
| 3β-CA | 3β-Cholic Acid | BAs | 407.4 | 343.4 | 408.287567 | [M-H]- | 5.06 | y = 0.01477 x + 0.00370 | 0.99802 | 20 | 1000 | 18.84178 | 39.12580 | 43.50820 |
| NCA | norcholic acid | BAs | 393.3 | 329.4 | 394.271912 | [M-H]- | 5.34 | y = 8.57726e-4 x - 4.81557e-5 | 0.99915 | 4 | 1000 | 1.29658 | 1.42551 | 1.01072 |
| 7-KDCA | 7-Ketodeoxycholic acid | BAs | 405.3 | 405.3 | 406.271912 | [M-H]- | 5.75 | y = 0.00337 x - 3.95454e-4 | 0.99949 | 2 | 400 | 56.24400 | 595.54000 | 627.38600 |
| 12-oxo-CDCA | 12-Oxochenodeoxycholic acid | BAs | 405.3 | 405.3 | 406.271912 | [M-H]- | 5.91 | y = 0.00516 x + 0.00126 | 0.99833 | 1 | 400 | 7.53662 | 108.03600 | 107.94620 |
| CDCA-3S | chenodeoxycholic acid3-sulfate disodium salt | BAs | 471.249 | 471.249 | 472.249475 | [M-H]- | 6.68 | y = 0.01600 x - 9.67096e-4 | 0.99887 | 4 | 100 | 3.71834 | 2.46300 | 2.67792 |
| HCA | hyocholic acid | BAs | 407.4 | 407.4 | 408.287567 | [M-H]- | 7.37 | y = 0.00377 x - 2.31425e-4 | 0.99869 | 10 | 400 | 28.29840 | 860.46400 | 902.91200 |
| MDCA | murideoxycholic acid | BAs | 391.3 | 391.3 | 392.292664 | [M-H]- | 8.01 | y = 0.01031 x + 3.58406e-5 | 0.99758 | 0.2 | 400 | 1.65268 | 10.23976 | 10.03804 |
| 3-oxo-CA | 3-Oxocholic acid | BAs | 405.3 | 289.4 | 406.271912 | [M-H]- | 8.16 | y = 0.04088 x + 0.00136 | 0.99831 | 2 | 400 | 0.25293 | 3.84958 | 3.77112 |
| CA | cholic acid | BAs | 407.4 | 343.4 | 408.287567 | [M-H]- | 8.4 | y = 8.30407e-4 x + 3.52329e-4 | 0.99722 | 1 | 400 | 19576.26000 | 102689.80000 | 99688.60000 |
| 3β-HDCA | β-Hyodeoxycholic Acid | BAs | 391.3 | 391.3 | 392.292664 | [M-H]- | 8.52 | y = 0.01189 x - 4.72480e-4 | 0.99983 | 4 | 400 | 2.84820 | 4.48738 | 3.98676 |
| UDCA | Ursodeoxycholic acid | BAs | 391.3 | 391.3 | 392.292664 | [M-H]- | 8.74 | y = 0.01869 x + 0.00250 | 0.9998 | 10 | 400 | 2.26738 | 69.28860 | 73.86900 |
| HDCA | Hyodeoxycholic acid | BAs | 391.3 | 391.3 | 392.292664 | [M-H]- | 8.85 | y = 0.00462 x + 1.11939e-4 | 0.99961 | 2 | 400 | 6.83046 | 558.57200 | 608.37000 |
| 6-ketoLCA | 5-β-Cholanic Acid-3α-ol-6-one | BAs | 389.277 | 389.277 | 390.27701 | [M-H]- | 9.17 | y = 0.01035 x + 7.29575e-4 | 0.99938 | 4 | 100 | 0.72289 | 37.39580 | 36.86600 |
| isoCDCA | Isochenodeoxycholic Acid | BAs | 391.293 | 391.293 | 392.29266 | [M-H]- | 9.39 | y = 0.00963 x + 9.11127e-4 | 0.99893 | 4 | 100 | 6.04892 | 412.10600 | 423.24800 |
| 3β-DCA | 3β-deoxycholic acid | BAs | 391.3 | 391.3 | 392.292664 | [M-H]- | 9.46 | y = 0.00692 x + 0.00177 | 0.99419 | 0.4 | 400 | 5.31064 | 102.00140 | 98.00340 |
| 7-KLCA | 7-ketolithocholic acid | BAs | 389.3 | 389.3 | 390.277008 | [M-H]- | 9.48 | y = 0.00654 x + 0.00123 | 0.9981 | 40 | 1000 | 36.60500 | 1739.81800 | 1780.61800 |
| 23-DCA | Nor-Deoxycholic Acid | BAs | 377.3 | 377.3 | 378.277008 | [M-H]- | 9.56 | y = 0.00854 x + 1.29342e-4 | 0.99643 | 4 | 400 | 0.99781 | 2.17384 | 1.82200 |
| 6,7-DKLCA | 6,7-diketolithocholic acid | BAs | 403.3 | 347.4 | 404.256287 | [M-H]- | 9.61 | y = 0.00207 x + 7.81362e-5 | 0.99925 | 4 | 1000 | 0.22722 | 11.18720 | 11.72130 |
| 12-KLCA | 12-ketolithocholic acid | BAs | 389.3 | 389.3 | 390.277008 | [M-H]- | 9.63 | y = 0.01114 x + 0.00103 | 0.99954 | 4 | 400 | 25.26760 | 187.20520 | 183.29560 |
| GHCA | Glycohyocholic acid | Glyco | 464.3 | 74 | 465.309052 | [M-H]- | 1.85 | y = 0.00142 x + 1.05881e-4 | 0.99763 | 1 | 400 | 40.04200 | 13.18920 | 11.30978 |
| GUDCA | Glycoursodeoxycholic acid | Glyco | 448.3 | 73.8 | 449.314117 | [M-H]- | 2.56 | y = 0.02384 x - 4.23311e-4 | 0.9994 | 4 | 400 | 27.13500 | 8.37256 | 9.16572 |
| GCA | Glycocholic acid | Glyco | 464.3 | 74 | 465.309052 | [M-H]- | 2.65 | y = 0.01263 x + 0.00521 | 0.99966 | 10 | 400 | 33003.20000 | 15141.14000 | 16329.98000 |
| GCDCA | Glycochenodeoxycholic acid | Glyco | 448.3 | 74 | 449.314117 | [M-H]- | 5.72 | y = 0.01215 x + 0.00433 | 0.99876 | 20 | 400 | 178233.60000 | 59909.20000 | 62868.60000 |
| GDCA | Glycodeoxycholic acid | Glyco | 448.3 | 73.8 | 449.314117 | [M-H]- | 6.52 | y = 0.01322 x + 5.47028e-4 | 0.99792 | 20 | 400 | 176814.80000 | 54387.80000 | 58089.40000 |
| GLCA | Glycolithocholic acid | Glyco | 432.3 | 74 | 433.319214 | [M-H]- | 9.45 | y = 0.00248 x + 0.00425 | 0.99944 | 20 | 1000 | 13.72604 | 2.80518 | 3.29648 |
| TCA-3S | Taurocholic Acid 3 sulfate sodium salt | Tauro | 594.248 | 594.248 | 595.248489 | [M-H]- | 0.96 | y = 0.42868 x + 0.00509 | 0.99975 | 40 | 1000 | 1.37216 | 10.05288 | 8.07548 |
| Tβ-MCA | Tauro-β-muricholic acid | Tauro | 514.3 | 79.8 | 515.291687 | [M-H]- | 0.96 | y = 0.03238 x - 0.00354 | 0.9944 | 4 | 400 | 13.45446 | 11.22536 | 11.26898 |
| Tω-MCA | Tauro-ω-muricholic Acid sodium salt | Tauro | 514.292 | 514.292 | 515.291674 | [M-H]- | 1.19 | y = 0.09611 x + 0.00398 | 0.99669 | 4 | 100 | 876.31600 | 496.59800 | 408.96600 |
| TDHCA | Taurodehydrocholic acid | Tauro | 508.3 | 79.9 | 509.24472 | [M-H]- | 1.22 | y = 9.88231e-4 x - 4.76889e-4 | 0.99873 | 20 | 400 | 58.95940 | 22.51380 | 23.90320 |
| Tα-MCA | Tauro-α-muricholicAcid sodium salt | Tauro | 514.292 | 514.292 | 515.291674 | [M-H]- | 1.24 | y = 0.19393 x + 0.01859 | 0.99714 | 4 | 100 | 145.43120 | 85.27220 | 72.53880 |
| THCA | Taurohyocholic acid | Tauro | 514.3 | 79.8 | 515.291687 | [M-H]- | 1.57 | y = 0.02373 x + 9.94845e-4 | 0.99802 | 10 | 1000 | 2161.10000 | 993.19600 | 912.99200 |
| THDCA | Taurohyodeoxycholic Acid (sodium salt) | Tauro | 498.297 | 498.297 | 499.296759 | [M-H]- | 2.09 | y = 0.01070 x - 3.55609e-4 | 0.99754 | 4 | 100 | 2449.32000 | 758.64800 | 743.02000 |
| TUDCA | Tauroursodeoxycholic acid | Tauro | 498.3 | 79.8 | 499.296753 | [M-H]- | 2.1 | y = 0.01088 x + 1.52629e-4 | 0.9997 | 4 | 400 | 2444.52000 | 493.90000 | 489.21000 |
| TCA | Taurocholic acid | Tauro | 514.3 | 79.8 | 515.291687 | [M-H]- | 2.26 | y = 0.02039 x + 0.00713 | 0.99636 | 0.4 | 400 | 88337.20000 | 55809.20000 | 46954.80000 |
| TCDCA | Taurochenodeoxycholic acid | Tauro | 498.3 | 79.8 | 499.296753 | [M-H]- | 4.71 | y = 0.00494 x + 3.34080e-4 | 0.99898 | 10 | 1000 | 260156.00000 | 64899.80000 | 60384.60000 |
| TDCA | Taurodeoxycholic acid | Tauro | 498.3 | 79.8 | 499.296753 | [M-H]- | 5.48 | y = 9.97144e-4 x - 2.63129e-4 | 0.99641 | 10 | 400 | 216418.00000 | 71780.60000 | 78094.80000 |
| TLCA | taurolithocholic acid | Tauro | 482.3 | 80 | 483.301849 | [M-H]- | 8.68 | y = 0.04481 x - 0.00528 | 0.9991 | 10 | 1000 | 15.23990 | 4.50008 | 3.59268 |

**Table S4.** The distribution of bile acid metabolism genes among the microbial genomes screened.

| **Genome** | **bsh** | **baiB** | **baiCD** | **baiE** | **baiA2** | **baiF** | **baiG** | **baiH** | **baiI** | **3 alpha HSDH** | **3 beta HSDH** | **7 alpha HSDH** | **7 beta HSDH** | **12 alpha HSDH** |
| --- | --- | --- | --- | --- | --- | --- | --- | --- | --- | --- | --- | --- | --- | --- |
| **XA768** | 1 | 0 | 0 | 0 | 0 | 0 | 0 | 0 | 0 | 0 | 0 | 0 | 0 | 0 |
| **XA1069** | 1 | 0 | 0 | 0 | 0 | 0 | 0 | 0 | 0 | 0 | 0 | 0 | 0 | 0 |
| **XA1416** | 2 | 0 | 0 | 0 | 1 | 0 | 0 | 0 | 0 | 0 | 0 | 1 | 0 | 0 |
| **XA314** | 3 | 0 | 0 | 1 | 1 | 0 | 0 | 0 | 0 | 0 | 1 | 1 | 0 | 0 |
| **XA1102** | 1 | 0 | 0 | 0 | 0 | 0 | 0 | 0 | 0 | 0 | 0 | 0 | 0 | 0 |
| **XA7822** | 1 | 0 | 0 | 0 | 0 | 0 | 0 | 0 | 0 | 0 | 0 | 0 | 0 | 0 |

***XA768: Bifidobacterium animalis; XA1069: Bifidobacterium adolescentis; XA1416:** *Ligilactobacillus salivarius***; XA314:** *Lactiplantibacillus plantarum***; XA1102:** *Bifidobacterium longum***; XA7822:** *Bifidobacterium longum subsp. suillum*

**Table S5.** BLASTp and PLMsearch results for bild acid metabolism genes among the selected genomes.

| **Genome** | **Species** | **Gene** | **N** | **BLASTp** | | | | **PLM search** | | | |
| --- | --- | --- | --- | --- | --- | --- | --- | --- | --- | --- | --- |
|  |  |  |  | **Reference** | **Reference Taxonomy** | **Identity** | **AlignLength(aa)** | **UniProt** | **Similarity** | **AlignScore** | **Reference Taxonomy** |
| **bsh** | | | | | | | | | | | |
| XA1069 | *Bifidobacterium adolescentis* | Chr1_926 | 1 | AAX86039.1 | Bifidobacterium adolescentis | 98.734 | 316 | P0DXD2 | 0.999800026 | 371.344696 | Bifidobacterium longum |
| XA1102 | *Bifidobacterium longum* | Chr1_847 | 1 | sp\|P0DXD2.1\|CBH_BIFL2 | Bifidobacterium longum | 100 | 317 | P0DXD2 | 1 | 373.1599121 | Bifidobacterium longum |
| XA1416 | *Ligilactobacillus salivarius* | Plas1_91 | 2 | AKI05275.1 | Ligilactobacillus salivarius | 98.457 | 324 | P54965 | 0.999199986 | 365.6496887 | Clostridium perfringens |
| XA1416 | *Ligilactobacillus salivarius* | Chr1_530 | 2 | AFP87506.1 | Ligilactobacillus salivarius | 97.846 | 325 | P0DXD2 | 0.996999979 | 354.0718994 | Bifidobacterium longum |
| XA314 | *Lactiplantibacillus plantarum* | Contig_13_chromosome_98 | 3 | AOZ56968.1 | Lactiplantibacillus plantarum | 99.685 | 317 | P54965 | 0.588013605 | NA | Clostridium perfringens |
| XA314 | *Lactiplantibacillus plantarum* | Contig_5_chromosome_168 | 3 | WPJ73070.1 | Lactiplantibacillus plantarum | 100 | 324 | Q06115 | 1 | 379.9623108 | Lactiplantibacillus plantarum |
| XA314 | *Lactiplantibacillus plantarum* | Contig_5_chromosome_23 | 3 | AOZ56969.1 | Lactiplantibacillus plantarum | 100 | 328 | P0DXD2 | 0.998000026 | 358.618103 | Bifidobacterium longum |
| XA768 | *Bifidobacterium animalis* | Contig_10_chromosome_51 | 1 | EHN18026.1 | Bifidobacterium animalis | 100 | 314 | Q9KK62 | 0.998700023 | 361.1538391 | Bifidobacterium longum |
| XA7822 | *Bifidobacterium longum* | Contig_1_chromosome_307 | 1 | sp\|P0DXD2.1\|CBH_BIFL2 | Bifidobacterium longum | 100 | 317 | P0DXD2 | 1 | 373.16 | Bifidobacterium longum |
| **baiE** | | | | | | | | | | | |
| XA314 | *Lactiplantibacillus plantarum* | Contig_16_chromosome_26 | 1 | WP_003642396.1 | Lactiplantibacillus plantarum | 100.00% | 147 | P19412 | 0.995000005 | 369.5834045 | Clostridium scindens |
| **baiA2** | | | | | | | | | | | |
| XA1416 | *Ligilactobacillus salivarius* | Contig_3_chromosome_80 | 1 | WP_003699078.1 | Ligilactobacillus | 100.00% | 275 | P19337 | 0.996599972 | 377.7898865 | Clostridium scindens |
| XA314 | *Lactiplantibacillus plantarum* | Contig_5_chromosome_136 | 1 | WP_069137243.1 | Lactiplantibacillus plantarum | 100.00% | 279 | P19337 | 0.996800005 | 376.7210693 | Clostridium scindens |
| **3beta HSDH** | | | | | | | | | | | |
| XA314 | *Lactiplantibacillus plantarum* | Contig_5_chromosome_136 | 1 | WP_069137243.1 | Lactiplantibacillus plantarum | 100.00% | 279 | A7AZH2 | 0.996500015 | 373.983551 | Mediterraneibacter gnavus |
| **7alpha HSDH** | | | | | | | | | | | |
| XA1416 | *Ligilactobacillus salivarius* | Contig_3_chromosome_80 | 1 | WP_003699078.1 | Ligilactobacillus | 100.00% | 275 | G9FRD7 | 0.997699976 | 378.219696 | Clostridium sardiniense |
| XA314 | *Lactiplantibacillus plantarum* | Contig_5_chromosome_136 | 1 | WP_069137243.1 | Lactiplantibacillus plantarum | 100.00% | 279 | G9FRD7 | 0.997699976 | 376.7202759 | Clostridium sardiniense |

**Table S6.** Detail information on the 5 distinct clusters of ileal content bile acid metabolome reflecting distinct effects of XA1416 and Orlistat on ileum contents metabolite profiles in dietary-induced obesity mice derived by using the fuzzy C-means algorithm incorporated with clustering in time-serial data method. Data are mean ± standard deviation (SD). Significant differences between means were determined by ANOVA. NC: normal diet. HFD: high-fat diet. Orlistat: administered with orlistat by gavage (10 mg/kg, n=8), XA1416: administered with XA1416 by gavage (2×10^8^ CFU/mouse, n=8).

| Name | HFD | NC | Orlistat | XA1416 | ANOVA p |
| --- | --- | --- | --- | --- | --- |
| Cluster1 | | | | | |
| Hyodeoxycholic acid | 6189.544 ± 2031.52 b | 5452.917 ± 2169.066 b | 4261.043 ± 4040.892 b | 18084.518 ± 4510.596 a | 1.71E-08 |
| Ursodeoxycholic acid | 10327.821 ± 3623.982 b | 13038.432 ± 8856.947 b | 5164.282 ± 4882.125 b | 31893.427 ± 11787.79 a | 2.75E-06 |
| beta-Hyodeoxycholic Acid | 10354.541 ± 3736.599 b | 13116.221 ± 8958.886 b | 5162.673 ± 4893.157 b | 31694.497 ± 11549.508 a | 2.89371E-06 |
| Tauro-alpha-muricholic acid | 6902.331 ± 2332.957 b | 17353.702 ± 9294.947 b | 9662.889 ± 3214.578 b | 33928.575 ± 14465.531 a | 9.45E-06 |
| Tauro-omega-muricholic acid | 6542.052 ± 2227.324 b | 16706.784 ± 9213.638 b | 9642.692 ± 3143.59 b | 31554.479 ± 13284.714 a | 1.47E-05 |
| Glycohyocholic acid | 30.505 ± 12.393 b | 52.171 ± 24.482 b | 35.288 ± 21.756 b | 94.708 ± 41.115 a | 0.000297496 |
| Cluster2 | | | | | |
| Apocholic acid | 109.368 ± 41.093 b | 42.055 ± 11.632 c | 48.098 ± 40.081 c | 241.535 ± 33.865 a | 3.70044E-12 |
| Glycohyodeoxycholic acid | 468.074 ± 297.961 b | 78.897 ± 27.142 b | 263.913 ± 189.22 b | 1116.575 ± 473.681 a | 9.09148E-07 |
| Glycoursodeoxycholic acid | 456.819 ± 298.86 b | 82.677 ± 30.369 b | 339.792 ± 213.033 b | 1114.673 ± 462.884 a | 1.50512E-06 |
| Tauro-beta-muricholic acid | 14365.485 ± 4844.476 b | 19107.118 ± 12766.025 b | 19263.034 ± 9740.054 b | 59287.332 ± 22794.325 a | 2.44358E-06 |
| Deoxycholic acid | 42348.514 ± 18868.453 b | 23721.507 ± 10773.245 b | 24886.09 ± 25987.26 b | 70839.658 ± 13952.552 a | 4.75882E-05 |
| Chenodeoxycholic acid | 11716.421 ± 4636.502 ab | 5441.164 ± 1950.224 b | 6985.006 ± 6785.774 b | 17202.143 ± 3219.852 a | 6.82254E-05 |
| Glycochenodeoxycholic acid | 1825.037 ± 1339.507 ab | 406.856 ± 400.061 c | 505.633 ± 380.276 bc | 2464.67 ± 1381.167 a | 0.000451646 |
| Glycodeoxycholic acid | 2200.537 ± 1809.966 ab | 457.77 ± 417.505 b | 1008.861 ± 834.201 ab | 2732.534 ± 1644.457 a | 0.005576963 |
| Glycocholic acid | 28307.493 ± 15761.139 a | 9760.253 ± 4663.385 b | 14403.698 ± 8296.517 ab | 24415.756 ± 11210.604 a | 0.006487127 |
| Cluster3 | | | | | |
| alpha-Muricholic acid | 21788.913 ± 10919.516 b | 38739.813 ± 17635.959 a | 7855.118 ± 7160.051 b | 47827.354 ± 9140.218 a | 1.50614E-06 |
| beta-Muricholic acid | 54166.919 ± 20322.162 bc | 100003.33 ± 33815.022 b | 46748.316 ± 38415.692 c | 148466.65 ± 33104.167 a | 2.82396E-06 |
| 7-Ketolithocholic acid | 312.783 ± 73.059 b | 922.892 ± 430.204 a | 154.768 ± 108.114 b | 678.59 ± 154.01 a | 2.91219E-06 |
| 3beta-Cholic Acid | 5554.914 ± 3364.582 bc | 10422.682 ± 5544.905 ab | 1807.423 ± 965.908 c | 10785.454 ± 2806.853 a | 5.14432E-05 |
| Hyocholic acid | 55 ± 19.757 bc | 292.82 ± 189.343 a | 45.539 ± 33.001 c | 189.771 ± 72.633 ab | 0.000146101 |
| 3-Dehydrocholic acid | 651.317 ± 151.007 b | 1457.82 ± 939.192 a | 298.19 ± 224.034 b | 1541.234 ± 453.337 a | 0.000153318 |
| Ursocholic acid | 1133.847 ± 745.03 b | 6978.914 ± 4695.907 a | 1138.924 ± 1186.411 b | 3995.757 ± 1152.757 ab | 0.000221231 |
| Cluster4 | | | | | |
| Lithocholic acid | 96.605 ± 70.26 b | 157.918 ± 89.805 b | 274.484 ± 182.992 b | 676.963 ± 155.575 a | 1.24604E-08 |
| Glycolithocholic acid | 7.391 ± 2.774 c | 4.211 ± 1.736 c | 17.64 ± 9.945 b | 29.572 ± 6.237 a | 2.82292E-08 |
| Tauroursodeoxycholic acid | 47111.849 ± 12379.99 a | 10925.725 ± 12384.95 b | 66056.864 ± 19095.601 a | 68987.257 ± 24437.914 a | 1.50946E-06 |
| Taurocholic acid | 12461.367 ± 1765.231 b | 13827.584 ± 2681.675 ab | 16692.185 ± 2198.517 a | 16274.246 ± 1741.044 a | 0.001661285 |
| Cluster5 | | | | | |
| 7-Dehydrocholic acid | 8109.66 ± 2374.422 c | 46354.823 ± 14279.519 a | 7715.136 ± 6864.738 c | 22202.357 ± 5272.012 b | 1.90291E-09 |
| Norcholic acid | 85.297 ± 33.917 b | 338.719 ± 184.781 a | 212.358 ± 99.347 ab | 122.966 ± 54.381 b | 0.000628742 |
| 12-Ketodeoxycholic acid | 1706.181 ± 708.288 a | 1738.488 ± 863.345 a | 1173.617 ± 759.751 a | 1604.805 ± 646.147 a | 0.42799203 |
| Cluster6 | | | | | |
| Taurochenodeoxycholic acid | 26151.195 ± 3355.136 b | 7574.375 ± 10737.667 c | 38797.409 ± 8236.237 a | 11281.449 ± 8382.919 c | 8.77793E-08 |
| Taurodeoxycholic acid | 23732.234 ± 5591.103 b | 8330.135 ± 11763.592 c | 43384.848 ± 15098.9 a | 10274.057 ± 6647.597 bc | 1.04972E-06 |
| Taurolithocholic acid | 2036.261 ± 1310.544 b | 528.434 ± 890.844 b | 7306.694 ± 4034.124 a | 1434.435 ± 1148.951 b | 8.45236E-06 |
| Taurohyodeoxycholic acid | 17241.825 ± 3564.84 b | 4226.872 ± 4353.32 c | 30470.285 ± 11717.71 a | 17337.78 ± 9129.577 b | 9.88666E-06 |
| Chenodeoxycholic acid 24-Acyl-beta-D-glucuronide | 120.546 ± 49.885 b | 56.209 ± 60.623 b | 350.257 ± 224.681 a | 39.269 ± 16.078 b | 5.85004E-05 |
| Isoallolithocholic acid | 9.509 ± 3.641 a | 16.403 ± 5.849 a | 21.043 ± 14.064 a | 12.356 ± 5.315 a | 0.064062821 |
| Cholic acid | 65938.287 ± 5694.143 a | 69652.69 ± 2935.437 a | 83773.468 ± 38679 a | 64357.162 ± 3484.246 a | 0.226500556 |

**Table S7.** Detail information on the 6 distinct clusters of gut microbiomes reflecting distinct effects of XA1416 and Orlistat on ileum contents metabolite profiles in high fat diet induced- gut microbiota disorders (including family level, genus level and species level) derived by using the fuzzy C-means algorithm incorporated with clustering in time-serial data method. Data are mean ± standard deviation (SD). Significant differences between means were determined by ANOVA. NC: normal diet. HFD: high-fat diet. Orlistat: administered with orlistat by gavage (10 mg/kg, n=8), XA1416: administered with XA1416 by gavage (2×10^8^ CFU/mouse, n=8).

| Name | NC | HFD | Orlistat | XA1416 | ANOVA p |
| --- | --- | --- | --- | --- | --- |
| Cluster1 | | | | | |
| f__Clostridia_unclassified | 0.097 ± 0.017 c | 0.418 ± 0.154 a | 0.044 ± 0.031 c | 0.252 ± 0.063 b | 4.13E-09 |
| f__Deferribacteraceae | 0.359 ± 0.343 a | 0.542 ± 0.84 a | 0.026 ± 0.011 a | 0.088 ± 0.032 a | 1.05E-01 |
| f__Enterobacteriaceae | 0.162 ± 0.017 b | 0.251 ± 0.029 a | 0.007 ± 0.007 c | 0.202 ± 0.055 b | 4.61294E-14 |
| f__Lachnospiraceae | 8.701 ± 1.921 b | 23.233 ± 6.837 a | 2.411 ± 1.362 c | 12.529 ± 4.808 b | 3.03E-09 |
| g__A2 | 0.302 ± 0.15 b | 1.763 ± 0.518 a | 0.528 ± 0.256 b | 0.718 ± 0.256 b | 3.83E-09 |
| g__Acetatifactor | 0.298 ± 0.086 b | 3.226 ± 1.805 a | 0.133 ± 0.085 b | 1.409 ± 0.891 b | 3.06624E-06 |
| g__Clostridia_unclassified | 0.097 ± 0.017 c | 0.418 ± 0.154 a | 0.044 ± 0.031 c | 0.252 ± 0.063 b | 4.13173E-09 |
| g__Escherichia.Shigella | 0.162 ± 0.017 b | 0.251 ± 0.029 a | 0.007 ± 0.007 c | 0.202 ± 0.055 b | 4.61294E-14 |
| g__Lachnoclostridium | 0.464 ± 0.121 c | 2.437 ± 0.719 a | 0.517 ± 0.289 bc | 1.13 ± 0.449 b | 2.50988E-09 |
| g__Lachnospiraceae_unclassified | 2.847 ± 0.739 c | 12.126 ± 3.484 a | 0.881 ± 0.737 c | 7.555 ± 2.969 b | 7.5654E-10 |
| g__Mucispirillum | 0.359 ± 0.343 a | 0.542 ± 0.84 a | 0.026 ± 0.011 a | 0.088 ± 0.032 a | 0.105080491 |
| g__Roseburia | 0.146 ± 0.026 b | 0.768 ± 0.485 a | 0.074 ± 0.04 b | 0.415 ± 0.362 ab | 0.000336078 |
| s__A2_unclassified | 0.302 ± 0.15 b | 1.763 ± 0.518 a | 0.528 ± 0.256 b | 0.718 ± 0.256 b | 3.83283E-09 |
| s__Acetatifactor_unclassified | 0.298 ± 0.086 b | 3.226 ± 1.805 a | 0.133 ± 0.085 b | 1.409 ± 0.891 b | 3.06624E-06 |
| s__Clostridia_unclassified | 0.097 ± 0.017 c | 0.418 ± 0.154 a | 0.044 ± 0.031 c | 0.252 ± 0.063 b | 4.13173E-09 |
| s__Escherichia.Shigella_unclassified | 0.162 ± 0.017 b | 0.251 ± 0.029 a | 0.007 ± 0.007 c | 0.202 ± 0.055 b | 4.61294E-14 |
| s__Lachnoclostridium_unclassified | 0.464 ± 0.121 c | 2.437 ± 0.719 a | 0.517 ± 0.289 bc | 1.13 ± 0.449 b | 2.50988E-09 |
| s__Lachnospiraceae_NK4A136_group_bacterium | 0.271 ± 0.169 b | 0.593 ± 0.309 a | 0.05 ± 0.04 b | 0.267 ± 0.117 b | 4.42395E-05 |
| s__Lachnospiraceae_unclassified | 2.847 ± 0.739 c | 12.126 ± 3.484 a | 0.881 ± 0.737 c | 7.555 ± 2.969 b | 7.5654E-10 |
| s__Mucispirillum_schaedleri | 0.359 ± 0.343 a | 0.542 ± 0.84 a | 0.026 ± 0.011 a | 0.088 ± 0.032 a | 0.105080491 |
| s__Roseburia_unclassified | 0.146 ± 0.026 b | 0.768 ± 0.485 a | 0.074 ± 0.04 b | 0.415 ± 0.362 ab | 0.000336078 |
| Cluster2 | | | | | |
| f__Akkermansiaceae | 4.085 ± 4.187 b | 0.012 ± 0.007 b | 18.176 ± 18.056 a | 0.009 ± 0.008 b | 0.001248568 |
| f__Bacteroidaceae | 1.79 ± 0.746 b | 3.727 ± 3.506 b | 13.575 ± 5.072 a | 6.686 ± 4.926 b | 1.18515E-05 |
| f__Eggerthellaceae | 0.032 ± 0.014 b | 0.09 ± 0.034 b | 0.926 ± 0.519 a | 0.064 ± 0.019 b | 1.52026E-07 |
| f__Erysipelatoclostridiaceae | 0.034 ± 0.037 b | 0.028 ± 0.01 b | 0.694 ± 0.534 a | 0.066 ± 0.092 b | 4.27793E-05 |
| f__Oscillospirales_unclassified | 0.086 ± 0.045 b | 0.17 ± 0.081 b | 0.685 ± 0.605 a | 0.166 ± 0.098 b | 0.002119384 |
| f__Prevotellaceae | 1.489 ± 1.002 b | 0.113 ± 0.099 b | 4.042 ± 2.125 a | 0.411 ± 0.575 b | 1.40964E-06 |
| g__Akkermansia | 4.085 ± 4.187 b | 0.012 ± 0.007 b | 18.176 ± 18.056 a | 0.009 ± 0.008 b | 0.001248568 |
| g__Alloprevotella | 1.009 ± 0.668 b | 0.099 ± 0.095 b | 4.042 ± 2.125 a | 0.387 ± 0.568 b | 4.25884E-07 |
| g__Bacteroides | 1.79 ± 0.746 b | 3.727 ± 3.506 b | 13.575 ± 5.072 a | 6.686 ± 4.926 b | 1.18515E-05 |
| g__Desulfovibrionaceae_unclassified | 0.018 ± 0.006 b | 0.111 ± 0.035 b | 0.351 ± 0.24 a | 0.097 ± 0.08 b | 0.000109118 |
| g__Enterorhabdus | 0.032 ± 0.014 b | 0.09 ± 0.034 b | 0.926 ± 0.519 a | 0.064 ± 0.019 b | 1.52026E-07 |
| g__Erysipelatoclostridium | 0.034 ± 0.037 b | 0.028 ± 0.01 b | 0.694 ± 0.534 a | 0.066 ± 0.092 b | 4.27793E-05 |
| g__Oscillospirales_unclassified | 0.086 ± 0.045 b | 0.17 ± 0.081 b | 0.685 ± 0.605 a | 0.166 ± 0.098 b | 0.002119384 |
| s__Akkermansia_muciniphila | 4.085 ± 4.187 b | 0.012 ± 0.007 b | 18.176 ± 18.056 a | 0.009 ± 0.008 b | 0.001248568 |
| s__Alloprevotella_unclassified | 1.009 ± 0.668 b | 0.099 ± 0.095 b | 4.042 ± 2.125 a | 0.387 ± 0.568 b | 4.25884E-07 |
| s__Bacteroides_unclassified | 0.796 ± 0.483 c | 2.663 ± 2.514 bc | 9.143 ± 3.643 a | 4.773 ± 3.47 b | 1.75137E-05 |
| s__Bacteroides_vulgatus | 0.108 ± 0.038 b | 0.298 ± 0.309 b | 3.405 ± 1.77 a | 0.558 ± 0.591 b | 1.93542E-07 |
| s__Desulfovibrionaceae_unclassified | 0.018 ± 0.006 b | 0.111 ± 0.035 b | 0.351 ± 0.24 a | 0.097 ± 0.08 b | 0.000109118 |
| s__Enterorhabdus_unclassified | 0.032 ± 0.014 b | 0.09 ± 0.034 b | 0.926 ± 0.519 a | 0.064 ± 0.019 b | 1.52026E-07 |
| s__Erysipelatoclostridium_unclassified | 0.034 ± 0.037 b | 0.028 ± 0.01 b | 0.694 ± 0.534 a | 0.066 ± 0.092 b | 4.27793E-05 |
| s__Oscillospirales_unclassified | 0.086 ± 0.045 b | 0.17 ± 0.081 b | 0.685 ± 0.605 a | 0.166 ± 0.098 b | 0.002119384 |
| Cluster3 | | | | | |
| f__Desulfovibrionaceae | 0.268 ± 0.097 b | 1.748 ± 1.049 a | 0.704 ± 0.5 ab | 1.451 ± 1.769 ab | 0.035577929 |
| f__Oscillospiraceae | 0.942 ± 0.198 c | 4.07 ± 1.088 a | 0.575 ± 0.34 c | 2.812 ± 1.244 b | 7.44476E-09 |
| f__Peptostreptococcaceae | 0.725 ± 0.131 b | 6.303 ± 2.101 a | 0.477 ± 0.364 b | 4.586 ± 2.397 a | 3.24199E-08 |
| f__Ruminococcaceae | 0.039 ± 0.012 c | 0.185 ± 0.036 a | 0.02 ± 0.015 c | 0.127 ± 0.06 b | 7.67926E-10 |
| f__Streptococcaceae | 0.153 ± 0.021 b | 1.054 ± 0.326 a | 0.376 ± 0.118 b | 0.863 ± 0.206 a | 1.5958E-09 |
| g__Bilophila | 0.25 ± 0.094 b | 1.637 ± 1.023 a | 0.353 ± 0.325 ab | 1.353 ± 1.692 ab | 0.019034614 |
| g__Colidextribacter | 0.382 ± 0.073 b | 1.603 ± 0.401 a | 0.317 ± 0.191 b | 1.169 ± 0.519 a | 2.52815E-08 |
| g__GCA_900066575 | 0.057 ± 0.018 bc | 0.151 ± 0.073 a | 0.021 ± 0.02 c | 0.111 ± 0.039 ab | 8.89061E-06 |
| g__Lactococcus | 0.153 ± 0.021 b | 1.054 ± 0.326 a | 0.376 ± 0.118 b | 0.863 ± 0.206 a | 1.5958E-09 |
| g__Oscillibacter | 0.076 ± 0.019 b | 0.209 ± 0.076 a | 0.054 ± 0.022 b | 0.164 ± 0.064 a | 3.02165E-06 |
| g__Oscillospiraceae_unclassified | 0.484 ± 0.124 c | 2.258 ± 0.655 a | 0.204 ± 0.136 c | 1.479 ± 0.737 b | 1.28258E-08 |
| g__Romboutsia | 0.725 ± 0.131 b | 6.303 ± 2.101 a | 0.477 ± 0.364 b | 4.586 ± 2.397 a | 3.24199E-08 |
| g__Ruminococcaceae_unclassified | 0.039 ± 0.012 c | 0.185 ± 0.036 a | 0.02 ± 0.015 c | 0.127 ± 0.06 b | 7.67926E-10 |
| g__Tuzzerella | 0.164 ± 0.029 bc | 0.448 ± 0.212 a | 0.148 ± 0.074 c | 0.335 ± 0.129 ab | 0.000146875 |
| s__Bilophila_unclassified | 0.25 ± 0.094 b | 1.637 ± 1.023 a | 0.353 ± 0.325 ab | 1.353 ± 1.692 ab | 0.019034614 |
| s__Colidextribacter_unclassified | 0.382 ± 0.073 b | 1.603 ± 0.401 a | 0.317 ± 0.191 b | 1.169 ± 0.519 a | 2.52815E-08 |
| s__GCA_900066575_unclassified | 0.057 ± 0.018 bc | 0.151 ± 0.073 a | 0.021 ± 0.02 c | 0.111 ± 0.039 ab | 8.89061E-06 |
| s__Lactococcus_unclassified | 0.153 ± 0.021 b | 1.054 ± 0.326 a | 0.376 ± 0.118 b | 0.863 ± 0.206 a | 1.5958E-09 |
| s__Oscillibacter_unclassified | 0.076 ± 0.019 b | 0.209 ± 0.076 a | 0.054 ± 0.022 b | 0.164 ± 0.064 a | 3.02165E-06 |
| s__Oscillospiraceae_unclassified | 0.484 ± 0.124 c | 2.258 ± 0.655 a | 0.204 ± 0.136 c | 1.479 ± 0.737 b | 1.28258E-08 |
| s__Romboutsia_ilealis | 0.484 ± 0.099 b | 4.292 ± 1.483 a | 0.321 ± 0.243 b | 3.144 ± 1.677 a | 5.56169E-08 |
| s__Romboutsia_unclassified | 0.241 ± 0.037 b | 2.011 ± 0.641 a | 0.156 ± 0.126 b | 1.442 ± 0.721 a | 1.33077E-08 |
| s__Ruminococcaceae_unclassified | 0.039 ± 0.012 c | 0.185 ± 0.036 a | 0.02 ± 0.015 c | 0.127 ± 0.06 b | 7.67927E-10 |
| s__Tuzzerella_unclassified | 0.164 ± 0.029 bc | 0.448 ± 0.212 a | 0.148 ± 0.074 c | 0.335 ± 0.129 ab | 0.000146875 |
| Cluster4 | | | | | |
| f__Clostridiaceae | 0.708 ± 1.199 a | 0.447 ± 0.357 a | 0.038 ± 0.028 a | 0.854 ± 0.487 a | 0.102420926 |
| f__Erysipelotrichaceae | 16.84 ± 11.282 b | 31.608 ± 9.813 ab | 35.324 ± 17.639 a | 39.798 ± 10.808 a | 0.007598564 |
| f__Marinifilaceae | 0.181 ± 0.063 b | 1.19 ± 0.697 a | 0.208 ± 0.137 b | 1.403 ± 0.423 a | 5.76367E-07 |
| f__Rikenellaceae | 1.325 ± 0.308 b | 7.897 ± 7.12 a | 1.729 ± 1.604 b | 9.523 ± 4.18 a | 0.000533632 |
| g__Alistipes | 0.806 ± 0.162 b | 2.883 ± 2.388 a | 1.537 ± 1.581 ab | 3.508 ± 0.683 a | 0.003906831 |
| g__Butyricimonas | 0.181 ± 0.063 b | 1.19 ± 0.697 a | 0.208 ± 0.137 b | 1.403 ± 0.423 a | 5.76367E-07 |
| g__Clostridium_sensu_stricto_1 | 0.708 ± 1.199 a | 0.447 ± 0.357 a | 0.038 ± 0.028 a | 0.854 ± 0.487 a | 0.102420926 |
| g__Faecalibaculum | 7.056 ± 4.185 b | 30.877 ± 9.876 a | 35.324 ± 17.639 a | 38.515 ± 10.568 a | 2.9058E-05 |
| g__Rikenellaceae_RC9_gut_group | 0.519 ± 0.215 b | 5.014 ± 4.832 a | 0.192 ± 0.078 b | 6.015 ± 3.998 a | 0.000874066 |
| s__Alistipes_unclassified | 0.806 ± 0.162 b | 2.883 ± 2.388 a | 1.537 ± 1.581 ab | 3.508 ± 0.683 a | 0.003906831 |
| s__Bacteroides_acidifaciens | 0.885 ± 0.272 a | 0.766 ± 0.698 a | 1.027 ± 0.664 a | 1.355 ± 0.964 a | 0.376790766 |
| s__Butyricimonas_unclassified | 0.117 ± 0.047 b | 0.742 ± 0.417 a | 0.132 ± 0.093 b | 0.903 ± 0.258 a | 2.27742E-07 |
| s__Butyricimonas_virosa | 0.063 ± 0.018 b | 0.448 ± 0.285 a | 0.076 ± 0.048 b | 0.5 ± 0.181 a | 4.84365E-06 |
| s__Clostridium_sensu_stricto_1_unclassified | 0.708 ± 1.199 a | 0.447 ± 0.357 a | 0.038 ± 0.028 a | 0.854 ± 0.487 a | 0.102420926 |
| s__Faecalibaculum_rodentium | 7.056 ± 4.185 b | 30.877 ± 9.876 a | 35.324 ± 17.639 a | 38.515 ± 10.568 a | 2.9058E-05 |
| s__Rikenellaceae_RC9_gut_group_unclassified | 0.519 ± 0.215 b | 5.014 ± 4.832 a | 0.192 ± 0.078 b | 6.015 ± 3.998 a | 0.000874066 |
| Cluster5 | | | | | |
| f__Bifidobacteriaceae | 0.593 ± 0.508 a | 0.029 ± 0.008 b | 0.059 ± 0.033 b | 0.04 ± 0.023 b | 0.000190982 |
| f__Lactobacillaceae | 12.075 ± 4.052 a | 4.305 ± 2.612 b | 7.525 ± 3.386 ab | 6.671 ± 4.259 b | 0.001922877 |
| f__Muribaculaceae | 28.099 ± 6.786 a | 2.452 ± 0.935 b | 6.703 ± 4.06 b | 3.35 ± 1.084 b | 2.54311E-13 |
| f__Sutterellaceae | 1.043 ± 0.56 a | 0.061 ± 0.012 b | 0.001 ± 0.002 b | 0.057 ± 0.011 b | 3.33302E-08 |
| g__Bifidobacterium | 0.593 ± 0.508 a | 0.029 ± 0.008 b | 0.059 ± 0.033 b | 0.04 ± 0.023 b | 0.000190982 |
| g__Dubosiella | 8.237 ± 11.162 a | 0.567 ± 0.256 b | 0 ± 0 b | 1.11 ± 0.518 ab | 0.0204708 |
| g__Lachnospiraceae_NK4A136_group | 4.423 ± 1.455 a | 2.315 ± 0.745 b | 0.108 ± 0.065 c | 0.857 ± 0.307 c | 1.83025E-10 |
| g__Lactobacillus | 12.075 ± 4.052 a | 4.305 ± 2.612 b | 7.525 ± 3.386 ab | 6.671 ± 4.259 b | 0.001922877 |
| g__Muribaculaceae_unclassified | 27.149 ± 6.705 a | 2.42 ± 0.922 b | 6.451 ± 3.973 b | 3.267 ± 1.033 b | 4.21736E-13 |
| g__Muribaculum | 0.95 ± 0.443 a | 0.032 ± 0.017 b | 0.252 ± 0.182 b | 0.082 ± 0.063 b | 5.12271E-08 |
| g__Parasutterella | 1.043 ± 0.56 a | 0.061 ± 0.012 b | 0.001 ± 0.002 b | 0.057 ± 0.011 b | 3.33302E-08 |
| g__Prevotellaceae_UCG_001 | 0.48 ± 0.404 a | 0.014 ± 0.009 b | 0 ± 0 b | 0.024 ± 0.012 b | 7.23517E-05 |
| g__Turicibacter | 1.548 ± 1.113 a | 0.164 ± 0.027 b | 0 ± 0 b | 0.172 ± 0.028 b | 1.27722E-05 |
| s__Bifidobacterium_unclassified | 0.593 ± 0.508 a | 0.029 ± 0.008 b | 0.059 ± 0.033 b | 0.04 ± 0.023 b | 0.000190982 |
| s__Dubosiella_unclassified | 8.237 ± 11.162 a | 0.567 ± 0.256 b | 0 ± 0 b | 1.11 ± 0.518 ab | 0.0204708 |
| s__Lachnospiraceae_NK4A136_group_unclassified | 4.152 ± 1.356 a | 1.722 ± 0.54 b | 0.058 ± 0.05 c | 0.59 ± 0.219 c | 3.23935E-11 |
| s__Lactobacillus_reuteri | 0.344 ± 0.155 a | 0.105 ± 0.075 b | 0.022 ± 0.031 b | 0.104 ± 0.081 b | 2.04707E-06 |
| s__Lactobacillus_unclassified | 11.732 ± 3.958 a | 4.201 ± 2.572 b | 7.503 ± 3.38 ab | 6.567 ± 4.214 b | 0.002399581 |
| s__Muribaculaceae_unclassified | 27.149 ± 6.705 a | 2.42 ± 0.922 b | 6.451 ± 3.973 b | 3.267 ± 1.033 b | 4.21736E-13 |
| s__Muribaculum_intestinale | 0.95 ± 0.443 a | 0.032 ± 0.017 b | 0.252 ± 0.182 b | 0.082 ± 0.063 b | 5.12271E-08 |
| s__Parasutterella_excrementihominis | 1.043 ± 0.56 a | 0.061 ± 0.012 b | 0.001 ± 0.002 b | 0.057 ± 0.011 b | 3.33302E-08 |
| s__Prevotellaceae_UCG_001_unclassified | 0.48 ± 0.404 a | 0.014 ± 0.009 b | 0 ± 0 b | 0.024 ± 0.012 b | 7.23516E-05 |
| s__Turicibacter_unclassified | 1.548 ± 1.113 a | 0.164 ± 0.027 b | 0 ± 0 b | 0.172 ± 0.028 b | 1.27722E-05 |

**Table S8.** The estimation of causal effects of bile acids associated with obesity phenotype as outcome.

| ID. exposure | ID. outcome | Exposure | Outcome | Method | Number of snp | Beta coefficient | SE | P_value |
| --- | --- | --- | --- | --- | --- | --- | --- | --- |
| met-a-346 | ukb-a-248 | Ursodeoxycholic acid | BMI | Inverse variance weighted (multiplicative random effects) | 26 | -0.024621697 | 0.011469061 | 0.031809792 |
| met-a-346 | PROT-a-1212 | Ursodeoxycholic acid | Appetite | Inverse variance weighted (multiplicative random effects) | 32 | -0.352175877 | 0.107537375 | 0.001056934 |
| met-a-346 | PROT-a-1443 | Ursodeoxycholic acid | Insulin-like growth factorl | Inverse variance weighted (multiplicative random effects) | 32 | 0.006835527 | 0.10758818 | 0.949341144 |
| met-a-346 | EBI-a-GCST005185 | Ursodeoxycholic acid | Fasting insulin | Inverse variance weighted (multiplicative random effects) | 19 | -14.05534609 | 2.95769686 | 2.0129E-06 |
| met-a-346 | ukb-e-21002_CSA | Ursodeoxycholic acid | Weight | Inverse variance weighted (multiplicative random effects) | 29 | -0.13627083 | 0.060607873 | 0.024550602 |
| met-a-346 | ieu-b-4761 | Ursodeoxycholic acid | Fasting glucose | Inverse variance weighted (multiplicative random effects) | 23 | -0.062418953 | 0.02005437 | 0.001855186 |
| GCST90616309 | ukb-a-248 | Chenodeoxycholic acid | BMI | Inverse variance weighted (multiplicative random effects) | 381 | -0.017590328 | 0.001973628 | 4.9811E-19 |
| GCST90616309 | PROT-a-1212 | Chenodeoxycholic acid | Appetite | Inverse variance weighted (multiplicative random effects) | 475 | -0.096908827 | 0.017959094 | 6.81101E-08 |
| GCST90616309 | PROT-a-1443 | Chenodeoxycholic acid | Insulin-like growth factorl | Inverse variance weighted (multiplicative random effects) | 480 | 0.060241079 | 0.017870976 | 0.00074926 |
| GCST90616309 | EBI-a-GCST005185 | Chenodeoxycholic acid | Fasting insulin | Inverse variance weighted (multiplicative random effects) | 59 | -7.499685059 | 0.532963232 | 5.67106E-45 |
| GCST90616309 | ukb-e-21002_CSA | Chenodeoxycholic acid | Weight | Inverse variance weighted (multiplicative random effects) | 467 | -0.030101686 | 0.010128163 | 0.00295792 |
| GCST90616309 | ieu-b-4761 | Chenodeoxycholic acid | Fasting glucose | Inverse variance weighted | 114 | -0.000843131 | 0.005216056 | 0.871588219 |
| GCST90200221 | ukb-a-248 | Glycoursodeoxycholic acid | BMI | Inverse variance weighted (multiplicative random effects) | 161 | -0.000948962 | 0.000647488 | 0.1427558 |
| GCST90200221 | PROT-a-1212 | Glycoursodeoxycholic acid | Appetite | Inverse variance weighted (multiplicative random effects) | 156 | -0.038550568 | 0.005591132 | 5.38842E-12 |
| GCST90200221 | PROT-a-1443 | Glycoursodeoxycholic acid | Insulin-like growth factorl | Inverse variance weighted (multiplicative random effects) | 159 | -0.030500137 | 0.005613886 | 5.54203E-08 |
| GCST90200221 | EBI-a-GCST005185 | Glycoursodeoxycholic acid | Fasting insulin | Inverse variance weighted (multiplicative random effects) | 18 | -2.237691008 | 0.460482273 | 1.17712E-06 |
| GCST90200221 | ukb-e-21002_CSA | Glycoursodeoxycholic acid | Weight | Inverse variance weighted (multiplicative random effects) | 136 | -0.017611528 | 0.003324326 | 1.17223E-07 |
| GCST90200221 | ieu-b-4761 | Glycoursodeoxycholic acid | Fasting glucose | Inverse variance weighted | 26 | 0.005123383 | 0.001870926 | 0.006173498 |
| GCST90616254 | ukb-a-248 | Tauroursodeoxycholic acid | BMI | Inverse variance weighted (multiplicative random effects) | 212 | -0.005849362 | 0.003529805 | 0.097492235 |
| GCST90616254 | PROT-a-1212 | Tauroursodeoxycholic acid | Appetite | Inverse variance weighted (multiplicative random effects) | 182 | -0.114600422 | 0.041347982 | 0.005578007 |
| GCST90616254 | PROT-a-1443 | Tauroursodeoxycholic acid | Insulin-like growth factorl | Inverse variance weighted (multiplicative random effects) | 182 | 0.064123545 | 0.041277743 | 0.120311995 |
| GCST90616254 | EBI-a-GCST005185 | Tauroursodeoxycholic acid | Fasting insulin | Inverse variance weighted (multiplicative random effects) | 10 | 10.53633914 | 1.836418242 | 9.61184E-09 |
| GCST90616254 | ukb-e-21002_CSA | Tauroursodeoxycholic acid | Weight | Inverse variance weighted (multiplicative random effects) | 163 | -0.008808925 | 0.005657101 | 0.119436144 |
| GCST90616254 | ieu-b-4761 | Tauroursodeoxycholic acid | Fasting glucose | Inverse variance weighted | 16 | -0.017082816 | 0.020463619 | 0.403836177 |

**Table S9.** Cochran Q statistic of bile acids to obesity phenotype.

| ID. exposure | ID. outcome | Exposure | Outcome | Method | Pleiotropy_egger_intercept | Pleiotropy_se | Pleiotropy_p.value | Q | Q_df | Q_p.value |
| --- | --- | --- | --- | --- | --- | --- | --- | --- | --- | --- |
| ukb-a-248 | met-a-346 | Ursodeoxycholic acid | BMI | MR Egger | 0.000970563 | 0.001991672 | 0.63045975 | 17.09440472 | 24 | 0.844607831 |
| ukb-a-248 | met-a-346 | Ursodeoxycholic acid | BMI | Inverse variance weighted | 0.000970563 | 0.001991672 | 0.63045975 | 17.33187635 | 25 | 0.869405844 |
| ieu-b-4761 | met-a-346 | Ursodeoxycholic acid | Fasting glucose | MR Egger | 0.002067153 | 0.002494203 | 0.416548221 | 6.370125126 | 21 | 0.999087385 |
| ieu-b-4761 | met-a-346 | Ursodeoxycholic acid | Fasting glucose | Inverse variance weighted | 0.002067153 | 0.002494203 | 0.416548221 | 7.057006134 | 22 | 0.99891342 |
| ukb-e-21002_CSA | met-a-346 | Ursodeoxycholic acid | Weight | MR Egger | 0.002756272 | 0.00737579 | 0.711551979 | 39.98354263 | 27 | 0.051418805 |
| ukb-e-21002_CSA | met-a-346 | Ursodeoxycholic acid | Weight | Inverse variance weighted | 0.002756272 | 0.00737579 | 0.711551979 | 40.19033982 | 28 | 0.063589676 |
| PROT-a-1443 | met-a-346 | Ursodeoxycholic acid | Insulin-like growth factorl | MR Egger | -0.00900707 | 0.015402644 | 0.563072076 | 7.359955869 | 30 | 0.999992337 |
| PROT-a-1443 | met-a-346 | Ursodeoxycholic acid | Insulin-like growth factorl | Inverse variance weighted | -0.00900707 | 0.015402644 | 0.563072076 | 7.701916837 | 31 | 0.999993662 |
| PROT-a-1212 | met-a-346 | Ursodeoxycholic acid | Appetite | MR Egger | 0.008059744 | 0.015527922 | 0.607535178 | 30.49004682 | 30 | 0.440773711 |
| PROT-a-1212 | met-a-346 | Ursodeoxycholic acid | Appetite | Inverse variance weighted | 0.008059744 | 0.015527922 | 0.607535178 | 30.76385892 | 31 | 0.478156377 |
| EBI-a-GCST005185 | met-a-346 | Ursodeoxycholic acid | Fasting insulin | MR Egger | -1.112553611 | 0.129798474 | 1.40914E-07 | 417286.3012 | 17 | 0 |
| EBI-a-GCST005185 | met-a-346 | Ursodeoxycholic acid | Fasting insulin | Inverse variance weighted | -1.112553611 | 0.129798474 | 1.40914E-07 | 2220669.133 | 18 | 0 |
| ukb-a-248 | GCST90616309 | Chenodeoxycholic acid | BMI | MR Egger | -0.001606802 | 0.000516795 | 0.00201794 | 224.0377984 | 379 | 1 |
| ukb-a-248 | GCST90616309 | Chenodeoxycholic acid | BMI | Inverse variance weighted | -0.001606802 | 0.000516795 | 0.00201794 | 233.7047294 | 380 | 1 |
| ieu-b-4761 | GCST90616309 | Chenodeoxycholic acid | Fasting glucose | MR Egger | -0.00398811 | 0.002220902 | 0.075236014 | 100.3195165 | 112 | 0.777680839 |
| ieu-b-4761 | GCST90616309 | Chenodeoxycholic acid | Fasting glucose | Inverse variance weighted | -0.00398811 | 0.002220902 | 0.075236014 | 103.5441132 | 113 | 0.726668836 |
| ukb-e-21002_CSA | GCST90616309 | Chenodeoxycholic acid | Weight | MR Egger | -0.006948611 | 0.00220062 | 0.001694373 | 326.5834779 | 465 | 0.999999797 |
| ukb-e-21002_CSA | GCST90616309 | Chenodeoxycholic acid | Weight | Inverse variance weighted | -0.006948611 | 0.00220062 | 0.001694373 | 336.5537231 | 466 | 0.999998631 |
| PROT-a-1443 | GCST90616309 | Chenodeoxycholic acid | Insulin-like growth factorl | MR Egger | 0.007313698 | 0.00500424 | 0.144535214 | 337.8533565 | 478 | 0.999999778 |
| PROT-a-1443 | GCST90616309 | Chenodeoxycholic acid | Insulin-like growth factorl | Inverse variance weighted | 0.007313698 | 0.00500424 | 0.144535214 | 339.9893401 | 479 | 0.999999708 |
| PROT-a-1212 | GCST90616309 | Chenodeoxycholic acid | Appetite | MR Egger | -0.002482366 | 0.005041033 | 0.62264246 | 315.909915 | 473 | 0.999999997 |
| PROT-a-1212 | GCST90616309 | Chenodeoxycholic acid | Appetite | Inverse variance weighted | -0.002482366 | 0.005041033 | 0.62264246 | 316.1524043 | 474 | 0.999999997 |
| EBI-a-GCST005185 | GCST90616309 | Chenodeoxycholic acid | Fasting insulin | MR Egger | 4.272070701 | 0.300466565 | 2.1378E-20 | 4416035.726 | 57 | 0 |
| EBI-a-GCST005185 | GCST90616309 | Chenodeoxycholic acid | Fasting insulin | Inverse variance weighted | 4.272070701 | 0.300466565 | 2.1378E-20 | 20077857.62 | 58 | 0 |
| ukb-a-248 | GCST90200221 | Glycoursodeoxycholic acid | BMI | MR Egger | -0.002212173 | 0.000551067 | 9.16279E-05 | 192.2182258 | 159 | 0.037238516 |
| ukb-a-248 | GCST90200221 | Glycoursodeoxycholic acid | BMI | Inverse variance weighted | -0.002212173 | 0.000551067 | 9.16279E-05 | 211.6999368 | 160 | 0.003869406 |
| ieu-b-4761 | GCST90200221 | Glycoursodeoxycholic acid | Fasting glucose | MR Egger | -0.007066746 | 0.001553307 | 0.000130588 | 5.921057147 | 24 | 0.999936889 |
| ieu-b-4761 | GCST90200221 | Glycoursodeoxycholic acid | Fasting glucose | Inverse variance weighted | -0.007066746 | 0.001553307 | 0.000130588 | 26.6188582 | 25 | 0.375189652 |
| ukb-e-21002_CSA | GCST90200221 | Glycoursodeoxycholic acid | Weight | MR Egger | -0.008699863 | 0.003122708 | 0.006110768 | 145.2495088 | 134 | 0.239071089 |
| ukb-e-21002_CSA | GCST90200221 | Glycoursodeoxycholic acid | Weight | Inverse variance weighted | -0.008699863 | 0.003122708 | 0.006110768 | 153.6629137 | 135 | 0.129777782 |
| PROT-a-1443 | GCST90200221 | Glycoursodeoxycholic acid | Insulin-like growth factorl | MR Egger | 0.008762313 | 0.004867467 | 0.073752202 | 50.23026588 | 157 | 1 |
| PROT-a-1443 | GCST90200221 | Glycoursodeoxycholic acid | Insulin-like growth factorl | Inverse variance weighted | 0.008762313 | 0.004867467 | 0.073752202 | 53.47091132 | 158 | 1 |
| PROT-a-1212 | GCST90200221 | Glycoursodeoxycholic acid | Appetite | MR Egger | 0.019734595 | 0.004958076 | 0.000105746 | 81.59696288 | 154 | 0.999999715 |
| PROT-a-1212 | GCST90200221 | Glycoursodeoxycholic acid | Appetite | Inverse variance weighted | 0.019734595 | 0.004958076 | 0.000105746 | 97.43969332 | 155 | 0.999913025 |
| EBI-a-GCST005185 | GCST90200221 | Glycoursodeoxycholic acid | Fasting insulin | MR Egger | -0.735976609 | 0.152159966 | 0.000182127 | 51424.20671 | 16 | 0 |
| EBI-a-GCST005185 | GCST90200221 | Glycoursodeoxycholic acid | Fasting insulin | Inverse variance weighted | -0.735976609 | 0.152159966 | 0.000182127 | 126616.7603 | 17 | 0 |
| ukb-a-248 | GCST90616254 | Tauroursodeoxycholic acid | BMI | MR Egger | -3.90349E-05 | 0.001318764 | 0.976414479 | 64.41103417 | 210 | 1 |
| ukb-a-248 | GCST90616254 | Tauroursodeoxycholic acid | BMI | Inverse variance weighted | -3.90349E-05 | 0.001318764 | 0.976414479 | 64.41191031 | 211 | 1 |
| ieu-b-4761 | GCST90616254 | Tauroursodeoxycholic acid | Fasting glucose | MR Egger | -0.003492955 | 0.00492871 | 0.490146813 | 5.823579988 | 14 | 0.970743953 |
| ieu-b-4761 | GCST90616254 | Tauroursodeoxycholic acid | Fasting glucose | Inverse variance weighted | -0.003492955 | 0.00492871 | 0.490146813 | 6.325829445 | 15 | 0.973739541 |
| ukb-e-21002_CSA | GCST90616254 | Tauroursodeoxycholic acid | Weight | MR Egger | -0.004698782 | 0.005803051 | 0.419302667 | 69.07668825 | 161 | 1 |
| ukb-e-21002_CSA | GCST90616254 | Tauroursodeoxycholic acid | Weight | Inverse variance weighted | -0.004698782 | 0.005803051 | 0.419302667 | 69.73231657 | 162 | 1 |
| PROT-a-1443 | GCST90616254 | Tauroursodeoxycholic acid | Insulin-like growth factorl | MR Egger | -0.001301532 | 0.013307911 | 0.922198842 | 92.91519958 | 180 | 0.99999999 |
| PROT-a-1443 | GCST90616254 | Tauroursodeoxycholic acid | Insulin-like growth factorl | Inverse variance weighted | -0.001301532 | 0.013307911 | 0.922198842 | 92.92476469 | 181 | 0.999999993 |
| PROT-a-1212 | GCST90616254 | Tauroursodeoxycholic acid | Appetite | MR Egger | -0.016490464 | 0.01499091 | 0.272787639 | 60.47880922 | 180 | 1 |
| PROT-a-1212 | GCST90616254 | Tauroursodeoxycholic acid | Appetite | Inverse variance weighted | -0.016490464 | 0.01499091 | 0.272787639 | 61.68887724 | 181 | 1 |
| EBI-a-GCST005185 | GCST90616254 | Tauroursodeoxycholic acid | Fasting insulin | MR Egger | 1.115452957 | 0.416649804 | 0.028047653 | 192180.9754 | 8 | 0 |
| EBI-a-GCST005185 | GCST90616254 | Tauroursodeoxycholic acid | Fasting insulin | Inverse variance weighted | 1.115452957 | 0.416649804 | 0.028047653 | 364360.1259 | 9 | 0 |

**Table S10.** The estimation of causal effects of obesity phenotype associated with bile acids as outcome.

| ID. exposure | ID. outcome | Exposure | Outcome | Method | Number of snp | Beta coefficient | SE | P_value |
| --- | --- | --- | --- | --- | --- | --- | --- | --- |
| ukb-a-248 | met-a-346 | BMI | Ursodeoxycholic acid | Inverse variance weighted (multiplicative random effects) | 36288 | -0.0225242 | 0.00244825 | 3.5755E-20 |
| PROT-a-1212 | met-a-346 | Appetite | Ursodeoxycholic acid | Inverse variance weighted (multiplicative random effects) | 24 | -0.0397286 | 0.00763339 | 1.9443E-07 |
| PROT-a-1443 | met-a-346 | Insulin-like growth factorl | Ursodeoxycholic acid | Inverse variance weighted (multiplicative random effects) | 40 | 0.01811588 | 0.00479033 | 0.00015572 |
| EBI-a-GCST005185 | met-a-346 | Fasting insulin | Ursodeoxycholic acid | Inverse variance weighted (multiplicative random effects) | 286 | -0.2315283 | 0.0353018 | 5.4337E-11 |
| ukb-e-21002_CSA | met-a-346 | Weight | Ursodeoxycholic acid | Inverse variance weighted (multiplicative random effects) | 31 | 0.02403999 | 0.01852632 | 0.19442045 |
| ieu-b-4761 | met-a-346 | Fasting glucose | Ursodeoxycholic acid | Inverse variance weighted (multiplicative random effects) | 376 | 0.01494817 | 0.01705132 | 0.38067251 |
| ukb-a-248 | GCST90616309 | BMI | Chenodeoxycholic acid | Inverse variance weighted (multiplicative random effects) | 32363 | -0.0421442 | 0.00524458 | 9.2997E-16 |
| PROT-a-1212 | GCST90616309 | Appetite | Chenodeoxycholic acid | Inverse variance weighted (multiplicative random effects) | 36 | 0.01031778 | 0.01908118 | 0.58869345 |
| PROT-a-1443 | GCST90616309 | Insulin-like growth factorl | Chenodeoxycholic acid | Inverse variance weighted (multiplicative random effects) | 73 | -0.0032139 | 0.01215451 | 0.79145554 |
| EBI-a-GCST005185 | GCST90616309 | Fasting insulin | Chenodeoxycholic acid | Inverse variance weighted (multiplicative random effects) | 98 | -0.0485915 | 0.08524988 | 0.56868552 |
| ukb-e-21002_CSA | GCST90616309 | Weight | Chenodeoxycholic acid | Inverse variance weighted (multiplicative random effects) | 44 | 0.00591327 | 0.02967973 | 0.84207813 |
| ieu-b-4761 | GCST90616309 | Fasting glucose | Chenodeoxycholic acid | Inverse variance weighted (multiplicative random effects) | 162 | -0.0577188 | 0.0528074 | 0.27439084 |
| ukb-a-248 | GCST90200221 | BMI | Glycoursodeoxycholic acid | Inverse variance weighted (multiplicative random effects) | 18877 | -4.6845811 | 0.22126579 | 1.74E-99 |
| PROT-a-1212 | GCST90200221 | Appetite | Glycoursodeoxycholic acid | Inverse variance weighted (multiplicative random effects) | 9 | -1.7102375 | 0.00382229 | 0 |
| PROT-a-1443 | GCST90200221 | Insulin-like growth factorl | Glycoursodeoxycholic acid | Inverse variance weighted (multiplicative random effects) | 74 | 3.75998317 | 0.26981415 | 3.8573E-44 |
| EBI-a-GCST005185 | GCST90200221 | Fasting insulin | Glycoursodeoxycholic acid | Inverse variance weighted (multiplicative random effects) | 54 | 0.15394566 | 1.0986746 | 0.88856563 |
| ukb-e-21002_CSA | GCST90200221 | Weight | Glycoursodeoxycholic acid | Inverse variance weighted (multiplicative random effects) | 25 | -2.4999436 | 0.56455608 | 9.5041E-06 |
| ieu-b-4761 | GCST90200221 | Fasting glucose | Glycoursodeoxycholic acid | Inverse variance weighted (multiplicative random effects) | 105 | -1.1422195 | 2.39844147 | 0.63390763 |
| ukb-a-248 | GCST90616254 | BMI | Tauroursodeoxycholic acid | Inverse variance weighted (multiplicative random effects) | 33281 | -0.056771 | 0.00393841 | 4.1822E-47 |
| PROT-a-1212 | GCST90616254 | Appetite | Tauroursodeoxycholic acid | Inverse variance weighted (multiplicative random effects) | 34 | 0.0348922 | 0.01497179 | 0.01977817 |
| PROT-a-1443 | GCST90616254 | Insulin-like growth factorl | Tauroursodeoxycholic acid | Inverse variance weighted (multiplicative random effects) | 70 | 0.01686978 | 0.00945061 | 0.0742539 |
| EBI-a-GCST005185 | GCST90616254 | Fasting insulin | Tauroursodeoxycholic acid | Inverse variance weighted (multiplicative random effects) | 107 | -0.0174289 | 0.06212407 | 0.77905567 |
| ukb-e-21002_CSA | GCST90616254 | Weight | Tauroursodeoxycholic acid | Inverse variance weighted (multiplicative random effects) | 46 | -0.0154769 | 0.02159638 | 0.47359307 |
| ieu-b-4761 | GCST90616254 | Fasting glucose | Tauroursodeoxycholic acid | Inverse variance weighted (multiplicative random effects) | 141 | 0.19849357 | 0.04276065 | 3.4511E-06 |

**Table S11.** Cochran Q statistic of obesity phenotype to bile acids.

| ID. exposure | ID. outcome | Exposure | Outcome | Method | Pleiotropy_egger_intercept | Pleiotropy_se | Pleiotropy_p.value | Q | Q_df | Q_p.value |
| --- | --- | --- | --- | --- | --- | --- | --- | --- | --- | --- |
| met-a-346 | ukb-a-248 | BMI | Ursodeoxycholic acid | MR Egger | -0.0005355 | 0.00012794 | 2.8522E-05 | 26177.7025 | 36286 | 1 |
| met-a-346 | ukb-a-248 | BMI | Ursodeoxycholic acid | Inverse variance weighted | -0.0005355 | 0.00012794 | 2.8522E-05 | 26195.2208 | 36287 | 1 |
| met-a-346 | ieu-b-4761 | Fasting glucose | Ursodeoxycholic acid | MR Egger | -0.0025545 | 0.00130862 | 0.05167632 | 221.02726 | 374 | 1 |
| met-a-346 | ieu-b-4761 | Fasting glucose | Ursodeoxycholic acid | Inverse variance weighted | -0.0025545 | 0.00130862 | 0.05167632 | 224.83782 | 375 | 1 |
| met-a-346 | ukb-e-21002_CSA | Weight | Ursodeoxycholic acid | MR Egger | -0.0121607 | 0.00466466 | 0.01427755 | 10.6299591 | 29 | 0.99927091 |
| met-a-346 | ukb-e-21002_CSA | Weight | Ursodeoxycholic acid | Inverse variance weighted | -0.0121607 | 0.00466466 | 0.01427755 | 17.4263744 | 30 | 0.96709992 |
| met-a-346 | PROT-a-1443 | Insulin-like growth factorl | Ursodeoxycholic acid | MR Egger | -0.0162306 | 0.00688146 | 0.02126877 | 37.1134584 | 67 | 0.99886693 |
| met-a-346 | PROT-a-1443 | Insulin-like growth factorl | Ursodeoxycholic acid | Inverse variance weighted | -0.0162306 | 0.00688146 | 0.02126877 | 42.6764703 | 68 | 0.99308563 |
| met-a-346 | PROT-a-1212 | Appetite | Ursodeoxycholic acid | MR Egger | -0.031699 | 0.01346316 | 0.02788606 | 4.60609274 | 22 | 0.99997016 |
| met-a-346 | PROT-a-1212 | Appetite | Ursodeoxycholic acid | Inverse variance weighted | -0.031699 | 0.01346316 | 0.02788606 | 10.1497515 | 23 | 0.99031201 |
| met-a-346 | EBI-a-GCST005185 | Fasting insulin | Ursodeoxycholic acid | MR Egger | 0.01749046 | 0.00186308 | 8.2008E-18 | 198.609071 | 219 | 0.83502051 |
| met-a-346 | EBI-a-GCST005185 | Fasting insulin | Ursodeoxycholic acid | Inverse variance weighted | 0.01749046 | 0.00186308 | 8.2008E-18 | 286.742626 | 220 | 0.00165019 |
| GCST90616309 | ukb-a-248 | BMI | Chenodeoxycholic acid | MR Egger | 0.10915273 | 0.01596473 | 8.3273E-12 | 1.5125E+11 | 18875 | 0 |
| GCST90616309 | ukb-a-248 | BMI | Chenodeoxycholic acid | Inverse variance weighted | 0.10915273 | 0.01596473 | 8.3273E-12 | 1.5163E+11 | 18876 | 0 |
| GCST90616309 | ieu-b-4761 | Fasting glucose | Chenodeoxycholic acid | MR Egger | 0.00119003 | 0.0041956 | 0.77705401 | 126.840974 | 160 | 0.97511081 |
| GCST90616309 | ieu-b-4761 | Fasting glucose | Chenodeoxycholic acid | Inverse variance weighted | 0.00119003 | 0.0041956 | 0.77705401 | 126.921425 | 161 | 0.97803228 |
| GCST90616309 | ukb-e-21002_CSA | Weight | Chenodeoxycholic acid | MR Egger | 1.07976494 | 1.45685553 | 0.46609622 | 887239.033 | 23 | 0 |
| GCST90616309 | ukb-e-21002_CSA | Weight | Chenodeoxycholic acid | Inverse variance weighted | 1.07976494 | 1.45685553 | 0.46609622 | 908429.389 | 24 | 0 |
| GCST90616309 | PROT-a-1443 | Insulin-like growth factorl | Chenodeoxycholic acid | MR Egger | -0.000681 | 0.00556192 | 0.90289272 | 29.1816651 | 71 | 0.9999974 |
| GCST90616309 | PROT-a-1443 | Insulin-like growth factorl | Chenodeoxycholic acid | Inverse variance weighted | -0.000681 | 0.00556192 | 0.90289272 | 29.1966578 | 72 | 0.99999835 |
| GCST90616309 | PROT-a-1212 | Appetite | Chenodeoxycholic acid | MR Egger | -0.0139036 | 0.01143191 | 0.23228078 | 18.6719037 | 34 | 0.98471409 |
| GCST90616309 | PROT-a-1212 | Appetite | Chenodeoxycholic acid | Inverse variance weighted | -0.0139036 | 0.01143191 | 0.23228078 | 20.1510742 | 35 | 0.97893314 |
| GCST90616309 | EBI-a-GCST005185 | Fasting insulin | Chenodeoxycholic acid | MR Egger | 0.01117324 | 0.00515449 | 0.03265666 | 40.7454522 | 96 | 0.99999987 |
| GCST90616309 | EBI-a-GCST005185 | Fasting insulin | Chenodeoxycholic acid | Inverse variance weighted | 0.01117324 | 0.00515449 | 0.03265666 | 45.44425 | 97 | 0.99999825 |
| GCST90200221 | ukb-a-248 | BMI | Glycoursodeoxycholic acid | MR Egger | 0.10915273 | 0.01596473 | 8.3273E-12 | 1.5125E+11 | 18875 | 0 |
| GCST90200221 | ukb-a-248 | BMI | Glycoursodeoxycholic acid | Inverse variance weighted | 0.10915273 | 0.01596473 | 8.3273E-12 | 1.5163E+11 | 18876 | 0 |
| GCST90200221 | ieu-b-4761 | Fasting glucose | Glycoursodeoxycholic acid | MR Egger | -0.5792285 | 0.29761027 | 0.05434739 | 8755725.3 | 103 | 0 |
| GCST90200221 | ieu-b-4761 | Fasting glucose | Glycoursodeoxycholic acid | Inverse variance weighted | -0.5792285 | 0.29761027 | 0.05434739 | 9077727.54 | 104 | 0 |
| GCST90200221 | ukb-e-21002_CSA | Weight | Glycoursodeoxycholic acid | MR Egger | 1.07976494 | 1.45685553 | 0.46609622 | 887239.033 | 23 | 0 |
| GCST90200221 | ukb-e-21002_CSA | Weight | Glycoursodeoxycholic acid | Inverse variance weighted | 1.07976494 | 1.45685553 | 0.46609622 | 908429.389 | 24 | 0 |
| GCST90200221 | PROT-a-1443 | Insulin-like growth factorl | Glycoursodeoxycholic acid | MR Egger | 0.8596183 | 0.34841541 | 0.01599571 | 589474.131 | 72 | 0 |
| GCST90200221 | PROT-a-1443 | Insulin-like growth factorl | Glycoursodeoxycholic acid | Inverse variance weighted | 0.8596183 | 0.34841541 | 0.01599571 | 639310.783 | 73 | 0 |
| GCST90200221 | PROT-a-1212 | Appetite | Glycoursodeoxycholic acid | MR Egger | -0.2892666 | 0.56334742 | 0.62342205 | 500508.789 | 7 | 0 |
| GCST90200221 | PROT-a-1212 | Appetite | Glycoursodeoxycholic acid | Inverse variance weighted | -0.2892666 | 0.56334742 | 0.62342205 | 519360.791 | 8 | 0 |
| GCST90200221 | EBI-a-GCST005185 | Fasting insulin | Glycoursodeoxycholic acid | MR Egger | -0.2615237 | 0.11099285 | 0.02226571 | 298161.388 | 52 | 0 |
| GCST90200221 | EBI-a-GCST005185 | Fasting insulin | Glycoursodeoxycholic acid | Inverse variance weighted | -0.2615237 | 0.11099285 | 0.02226571 | 329994.57 | 53 | 0 |
| GCST90616254 | ukb-a-248 | BMI | Tauroursodeoxycholic acid | MR Egger | 0.10915273 | 0.01596473 | 8.3273E-12 | 1.5125E+11 | 18875 | 0 |
| GCST90616254 | ukb-a-248 | BMI | Tauroursodeoxycholic acid | Inverse variance weighted | 0.10915273 | 0.01596473 | 8.3273E-12 | 1.5163E+11 | 18876 | 0 |
| GCST90616254 | ieu-b-4761 | Fasting glucose | Tauroursodeoxycholic acid | MR Egger | 0.00901629 | 0.00303428 | 0.00349338 | 120.435821 | 139 | 0.87008052 |
| GCST90616254 | ieu-b-4761 | Fasting glucose | Tauroursodeoxycholic acid | Inverse variance weighted | 0.00901629 | 0.00303428 | 0.00349338 | 129.265515 | 140 | 0.73189815 |
| GCST90616254 | ukb-e-21002_CSA | Weight | Tauroursodeoxycholic acid | MR Egger | 1.07976494 | 1.45685553 | 0.46609622 | 887239.033 | 23 | 0 |
| GCST90616254 | ukb-e-21002_CSA | Weight | Tauroursodeoxycholic acid | Inverse variance weighted | 1.07976494 | 1.45685553 | 0.46609622 | 908429.389 | 24 | 0 |
| GCST90616254 | PROT-a-1443 | Insulin-like growth factorl | Tauroursodeoxycholic acid | MR Egger | 0.02088536 | 0.00446027 | 1.4003E-05 | 41.1674434 | 68 | 0.99587138 |
| GCST90616254 | PROT-a-1443 | Insulin-like growth factorl | Tauroursodeoxycholic acid | Inverse variance weighted | 0.02088536 | 0.00446027 | 1.4003E-05 | 63.0935887 | 69 | 0.67748991 |
| GCST90616254 | PROT-a-1212 | Appetite | Tauroursodeoxycholic acid | MR Egger | -0.0117649 | 0.00771782 | 0.13723586 | 14.9462582 | 32 | 0.99554153 |
| GCST90616254 | PROT-a-1212 | Appetite | Tauroursodeoxycholic acid | Inverse variance weighted | -0.0117649 | 0.00771782 | 0.13723586 | 17.2700061 | 33 | 0.98894782 |
| GCST90616254 | EBI-a-GCST005185 | Fasting insulin | Tauroursodeoxycholic acid | MR Egger | 0.01579959 | 0.00367564 | 3.8582E-05 | 34.7556216 | 105 | 1 |
| GCST90616254 | EBI-a-GCST005185 | Fasting insulin | Tauroursodeoxycholic acid | Inverse variance weighted | 0.01579959 | 0.00367564 | 3.8582E-05 | 53.2323195 | 106 | 0.99999573 |

**Table S12.** Detail information on the 5 distinct clusters of liver bile acid metabolome reflecting distinct effects of XA1416 and Orlistat on liver contents metabolite profiles in dietary-induced obesity mice derived by using the fuzzy C-means algorithm incorporated with clustering in time-serial data method. Data are mean ± standard deviation (SD). Significant differences between means were determined by ANOVA. NC: normal diet. HFD: high-fat diet. Orlistat: administered with orlistat by gavage (10 mg/kg, n=8), XA1416: administered with XA1416 by gavage (2×10^8^ CFU/mouse, n=8).

| Name | HFD | | NC | | Orlistat | | XA1416 | | ANOVA p | |
| --- | --- | --- | --- | --- | --- | --- | --- | --- | --- | --- |
| Cluster1 | | | | | | | | | | |
| 12-KetoDCA | | 5.769 ± 11.907 b | | 38.035 ± 18.673 a | | 6.713 ± 7.973 b | | 4.701 ± 6.984 b | | 9.83775E-06 |
| 7-DHCA | | 147.463 ± 251.677 ab | | 1799.166 ± 2412.598 a | | 129.403 ± 164.206 b | | 139.452 ± 196.844 ab | | 0.023062342 |
| 7-KetoLCA | | 1.221 ± 2.381 b | | 12.125 ± 13.62 a | | 0.475 ± 1.343 b | | 0.714 ± 2.02 b | | 0.005458876 |
| 7,12-DiketoLCA | | 0 ± 0 a | | 0.788 ± 2.23 a | | 0 ± 0 a | | 0 ± 0 a | | 0.407360288 |
| CA | | 2386.376 ± 1000.928 b | | 7059.072 ± 4376.515 a | | 3107.209 ± 1505.764 ab | | 4561.426 ± 4261.793 ab | | 0.032883306 |
| CDCA | | 223.223 ± 98.982 a | | 292.779 ± 222.414 a | | 232.967 ± 34.038 a | | 259.942 ± 92.52 a | | 0.718654714 |
| GCA | | 109.609 ± 99.506 b | | 487.489 ± 343.081 a | | 258.579 ± 191.419 ab | | 168.223 ± 132.887 b | | 0.007668186 |
| GCDCA | | 2.153 ± 1.619 b | | 10.253 ± 8.051 a | | 5.632 ± 4.978 ab | | 4.742 ± 3.596 ab | | 0.029159315 |
| GDCA | | 4.077 ± 1.749 b | | 11.649 ± 2.941 a | | 7.68 ± 7.027 ab | | 6.02 ± 3.003 ab | | 0.008602273 |
| HCA | | 8.124 ± 2.518 b | | 23.244 ± 12.025 a | | 13.294 ± 3.645 b | | 11.751 ± 6.615 b | | 0.00187462 |
| NorCA | | 1.265 ± 2.531 a | | 12.395 ± 14.356 a | | 7.604 ± 6.362 a | | 2.649 ± 6.445 a | | 0.058693129 |
| UCA | | 30.568 ± 22.064 a | | 121.137 ± 122.419 a | | 63.685 ± 43.778 a | | 41.305 ± 26.414 a | | 0.053142947 |
| β-MCA | | 4877.226 ± 3138.332 b | | 11113.537 ± 3874.572 a | | 7661.108 ± 3160.339 ab | | 6123.896 ± 3469.467 b | | 0.00687111 |
| Cluster2 | | | | | | | | | | |
| DCA | 246.179 ± 302.136 a | | 167.807 ± 110.987 a | | 441.639 ± 924.101 a | | 177.691 ± 117.107 a | | 0.664047433 | |
| GUDCA | 3.341 ± 0.767 a | | 7.084 ± 6.186 a | | 7.001 ± 4.465 a | | 5.92 ± 4.435 a | | 0.312838966 | |
| MDCA | 481.202 ± 216.743 a | | 745.025 ± 549.305 a | | 838.516 ± 380.161 a | | 652.215 ± 327.795 a | | 0.314996951 | |
| T-α-MCA | 758.171 ± 337.259 b | | 3296.983 ± 2191.422 ab | | 3483.819 ± 2653.151 a | | 1515.372 ± 1849.11 ab | | 0.022442327 | |
| T-ω-MCA | 645.402 ± 369.421 b | | 1871.346 ± 717.074 ab | | 2656.263 ± 1591.75 a | | 1043.227 ± 1030.688 b | | 0.002648995 | |
| TDCA | 2312.356 ± 1004.452 a | | 5416.926 ± 3397.672 a | | 6366.257 ± 4928.774 a | | 3256.636 ± 1555.493 a | | 0.054376978 | |
| THDCA | 173.122 ± 85.8 a | | 779.633 ± 896.848 a | | 1209.547 ± 1212.421 a | | 448.298 ± 523.637 a | | 0.080340139 | |
| ω-MCA | 3101.288 ± 1209.678 b | | 5269.542 ± 1829.153 ab | | 6472.458 ± 1967.778 a | | 4920.179 ± 2157.447 ab | | 0.009085651 | |
| Cluster3 | | | | | | | | | | |
| CDCA-24G | 36.545 ± 18.612 a | | 225.853 ± 260.376 a | | 171.404 ± 213.213 a | | 82.078 ± 77.338 a | | 0.143527947 | |
| GHDCA | 1.62 ± 1.508 a | | 3.654 ± 3.308 a | | 3.59 ± 2.984 a | | 2.458 ± 1.783 a | | 0.325687562 | |
| T-β-MCA | 1282.487 ± 1744.803 b | | 4943.08 ± 2906.768 a | | 4108.481 ± 3374.891 ab | | 1667.196 ± 2082.385 ab | | 0.020762706 | |
| TCA | 8051.06 ± 1762.303 b | | 10953.736 ± 1304.832 a | | 10597.855 ± 1891.254 a | | 8802.392 ± 2030.281 ab | | 0.006674899 | |
| TCDCA | 953.083 ± 483.035 a | | 4121.789 ± 3991.074 a | | 3650.055 ± 3294.798 a | | 2068.646 ± 1737.011 a | | 0.102915689 | |
| TLCA | 53.646 ± 21.816 a | | 129.665 ± 117.899 a | | 114.614 ± 95.541 a | | 87.031 ± 60.632 a | | 0.288794814 | |
| TUDCA | 714.359 ± 443.373 a | | 4073.66 ± 3600.836 a | | 3849.699 ± 3249.993 a | | 1814.109 ± 2013.939 a | | 0.045599687 | |
| α-MCA | 1946.919 ± 854.11 b | | 4885.156 ± 2766.266 a | | 3874.411 ± 1657.567 ab | | 3002.763 ± 1775.802 ab | | 0.028159305 | |
| Cluster4 | | | | | | | | | | |
| IsoalloLCA | 10.373 ± 3.692 a | | 9.048 ± 4.325 a | | 6.415 ± 2.894 a | | 9.475 ± 2.074 a | | 0.128785337 | |
| NorDCA | 11.664 ± 1.695 a | | 10.843 ± 3.161 ab | | 7.813 ± 2.434 b | | 9.64 ± 2.702 ab | | 0.030431233 | |
| Cluster5 | | | | | | | | | | |
| 3-DHCA | 10.615 ± 6.15 a | | 20.454 ± 12.443 a | | 15.156 ± 8.138 a | | 22.986 ± 19.616 a | | 0.23166692 | |
| 3β-CA | 50.268 ± 31.983 a | | 39.394 ± 25.407 a | | 37.212 ± 27.81 a | | 106.934 ± 144.964 a | | 0.244117905 | |
| 3β-DCA | 18.43 ± 12.315 a | | 7.4 ± 5.166 a | | 10.827 ± 5.498 a | | 19.823 ± 11.429 a | | 0.032426703 | |
| 3β-UDCA | 36.915 ± 27.524 a | | 20.807 ± 29.544 a | | 30.024 ± 16.367 a | | 48.26 ± 59.456 a | | 0.510427738 | |
| Apocholic.acid | 1.101 ± 1.015 a | | 0.794 ± 1.519 a | | 1.05 ± 0.974 a | | 1.834 ± 1.118 a | | 0.344640465 | |
| DHCA | 0 ± 0 a | | 0 ± 0 a | | 0 ± 0 a | | 0.465 ± 1.316 a | | 0.407359279 | |
| HDCA | 414.9 ± 207.637 a | | 396.291 ± 353.891 a | | 510.447 ± 221.232 a | | 529.398 ± 187.547 a | | 0.641247151 | |
| LCA | 7.148 ± 3.566 ab | | 3.91 ± 2.368 bc | | 3.543 ± 0.723 c | | 9.486 ± 2.297 a | | 8.14505E-05 | |
| UDCA | 488.899 ± 242.584 a | | 684.524 ± 369.395 a | | 555.323 ± 159.135 a | | 778.809 ± 387.004 a | | 0.247763883 | |

**

**

**Figure S1.** *In-vitro* measurement of BSH activity among the selected microbes represented by (A) total BSH activity, (B) BSH activity towards taurine conjugated bile salts and (C) glycine conjugated bile salts. Numbers underneath the microbial ID indicates the bile salt concentration in ug/L. (D) The mRNA expression of *L. salivarius* XA1416 in mouse feces.


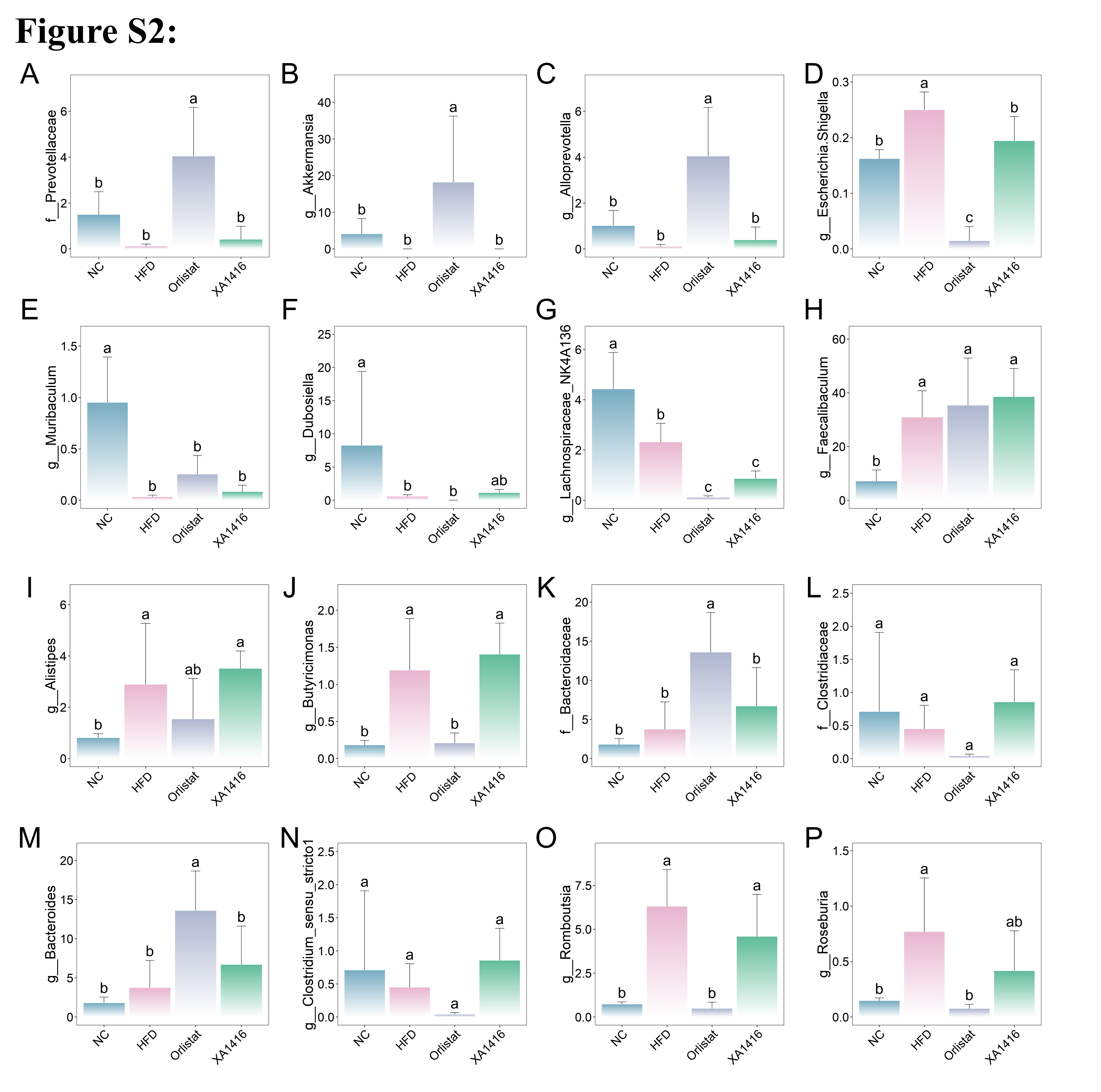


**Figure S2.** Relative abundances of (A) p_Prevotellaceae, (B) *g_Akkermansia*, (C) *g_Alloprevotella*, (D) *g_Escherichia Shigella*, (E) *g_Muribaculum*, (F) *g_Dubosiella*, (G) *g_Lachnospiraceae_NK4A136*, (H) *g_Eaecalibaculum*, (I) *g_Alistipes*, (J) *g_Butyricimonas*, (K) f_Bacyeroidaeae, (L) f_Clostridiaceae, (M) *g_Bacteroides*, (N) *g_Clostridium_sensu_stricto1*, (O) *g_Romboutsia*, (P) *g_Roseburia*. Differences between multiple groups were assessed by ANOVA, followed by the Tukey’s posthoc test. Significance was indicated by different letters (*p* < 0.05).





**Figure S3.** Analysis of molecular docking. (A-B) Molecular binding conformation of the GLP-1R–UDCA complex. (C-D) RMSD of the complex and the ligand. (E-F) Radius of gyration of the complex. (G-H) Solvent accessible surface area of the complex. (I-J) Number of secondary structures of the complex. (K-L) RMSF of the protein. (M-N) Hbonds of the complex. (O-P) Free Energy Contribution of the complex.
